# Author Correction: Identifying species likely threatened by international trade on the IUCN Red List can inform CITES trade measures

**DOI:** 10.1038/s41559-023-02228-0

**Published:** 2023-10-02

**Authors:** Daniel W. S. Challender, Patricia J. Cremona, Kelly Malsch, Janine E. Robinson, Alyson T. Pavitt, Janet Scott, Rachel Hoffmann, Ackbar Joolia, Thomasina E. E. Oldfield, Richard K. B. Jenkins, Dalia A. Conde, Craig Hilton-Taylor, Michael Hoffmann

**Affiliations:** 1 Interdisciplinary Centre for Conservation Science (ICCS), Department of Biology and Oxford Martin School, University of Oxford, Oxford, UK; 2 IUCN Science & Data Centre: Biodiversity Assessment & Knowledge Team, The David Attenborough Building, Cambridge, UK; 3 UN Environment Programme World Conservation Monitoring Centre (UNEP-WCMC), Cambridge, UK; 4 Durrell Institute of Conservation and Ecology (DICE), School of Anthropology and Conservation, University of Kent, Canterbury, UK; 5 Joint Nature Conservation Committee (JNCC), Peterborough, UK; 6 Sustainable Use and Livelihoods Specialist Group, Species Survival Commission/Commission on Environmental, Economic and Social Policy, International Union for Conservation of Nature (IUCN), Gland, Switzerland; 7 International Institute for Environment and Development (IIED), London, UK; 8 TRAFFIC, The David Attenborough Building, Cambridge, UK; 9 Independent Consultant, Cambridge, UK; 10 Species360 Conservation Science Alliance, Bloomington, MN USA; 11 Interdisciplinary Centre on Population Dynamics, Department of Biology, University of Southern Denmark, Odense, Denmark; 12 Conservation and Policy, Zoological Society of London, London, UK

**Keywords:** Biodiversity, Biodiversity, Zoology

Correction to: *Nature Ecology & Evolution* 10.1038/s41559-023-02115-8, published online 6 July 2023.

This paper was originally published under a standard Springer Nature license (© The Author(s), under exclusive licence to Springer Nature Limited 2023). It is now available as an open-access paper under a Creative Commons Attribution 4.0 International license, © The Author(s). The error has been corrected in the HTML and PDF versions of the article.

