# Supplementary material for: Identifying species likely threatened by international trade on the IUCN Red List can inform CITES trade measures
Source: Nat Ecol Evol. 2023 Jul 6;7(8):1211–20. doi: 10.1038/s41559-023-02115-8 (PMC10545538; doi:10.1038/s41559-023-02115-8)
Supplement: Supplementary file 1 — Supplementary Context, Methods, Tables 1–6, Results, Discussion and References. [file 41559_2023_2115_MOESM1_ESM.pdf]

nature ecology & evolution

Article

https://doi.org/10.1038/s41559-023-02115-8

Identifying species likely threatened by international trade on the IUCN Red List can inform CITES trade measures

In the format provided by the authors and unedited

Supplementary Information

Challender et al. **– Identifying species likely threatened by international trade on the IUCN Red List can inform CITES trade measures**

1. Supplementary Context **2**

2. Supplementary Methods **4**

2.1 The IUCN Red List of Threatened Species 4

2.2 Queries of the IUCN Red List 7

2.3 Limitations to IUCN Red List data 10

2.4 Species categorisation 12

2.5 Estimates of the proportion of species Likely threatened by international trade 18

2.6 The IUCN Red List Categories and Criteria and the CITES listing criteria 20

2.7 Aligning taxonomies between the IUCN Red List and CITES 23

2.8 Retrospective automated coding 24

3. Supplementary Tables and Figures **30**

Supplementary Table 1 30

Supplementary Table 2 80

Supplementary Table 3 81

Supplementary Table 4 82

Supplementary Table 5 83

Supplementary Table 6 85

4. Supplementary Results **86**

4.1 Proportion of species Likely threatened by international trade 86

4.2 Proportion of species Likely threatened by international trade – intentional use 86

4.3 Retrospective coding results 87

5. Supplementary Discussion **89**

5.1 Application of results 89

5.2 Feasibility of recommendations 94

5.3 Implications for the IUCN Red List 96

6. Supplementary References **99**

1. Supplementary Context

Research on the use and trade of wildlife has increased in the last decade[5], but there are prominent knowledge gaps regarding the prevalence of use and trade related threats at different scales, including local, national (i.e., domestic) and international. Existing research has quantified overall threats to biodiversity using the Red List[1] and more recently there have been attempts to quantify the threat to species from trade. For instance, one study[46] investigated the threat to species from trade using data from the Red List and CITES but assumed that all species included in the CITES Appendices are traded and conflated species being *"in trade"* with *"threatened by trade"*, undermining the results[47]. Another study[48] characterised the threat to species from trade using the Red List but similarly assumed that species that are traded are, by default, threatened by trade, which is not the case[47].

As part of a study examining time lags between Red List assessments being published and the subsequent inclusion of species in CITES, a further study[49] estimated the number of species for which the authors could "link international trade as a factor in their endangerment", and identified which of these species are, and are not, included in the CITES Appendices. However, this study focused on intentional use of species only, thereby overlooking unintentional uses (e.g., bycatch) despite these uses being known to negatively impact many taxa[50]; overlooked important coded information in assessments relating to use and trade, including the purpose of use (e.g., "Food-Human") and scale of use (e.g., "subsistence"); and overlooked whether the particular use or trade comprising the threat is international or not. Additionally, the methods used to link international trade as a factor in the endangerment of species in this study were not systematic and therefore are not repeatable. A further study[51] used a 2017 version of the Red List to crudely compare numbers of species on the Red List and those included in CITES and suggested that all threatened species on the Red List should

be included in CITES. However, this overlooks important differences between the Red List and CITES[47], including consideration of the threat from harvest for trade. A more recent study[34] carefully analysed IUCN Red List assessment data to understand whether biological resource use (BRU) is sustainable or unsustainable, but these authors did not consider the geographic scale at which this use takes place. Finally, a further study[52] examined species in international trade that are "at-risk" on the IUCN Red List and which of these species are included and excluded from CITES but the authors presented little information on the methods used and did not demonstrate the application of a systematic approach. Hence, despite these research efforts, there has been no robust attempt to systematically quantify the prevalence of threat to species from international trade using the Red List and nor is there an established mechanism by which to identify species threatened by international trade on the Red List to inform international policymaking. We provide the first systematic assessment of species' threat from international trade across all taxonomic groups, develop such a mechanism, situate the results in the context of all threats to species from BRU, consider threats to species from international trade as well as use and trade at the local and domestic level, and evaluate the feasibility of our proposed policy recommendations.

2. Supplementary Methods

2.1 The IUCN Red List of Threatened Species

The Red List is recognized internationally as the most comprehensive and objective approach for assessing the conservation status of animals, plants and fungi species globally. It is designed to determine the relative extinction risk for species and highlight those facing a high risk of global extinction. It provides information on the taxonomy, conservation status, threats, distribution, and use and trade of species that have been globally evaluated using the Red List Categories and Criteria[53].

The Red List Categories and Criteria are designed to be an easily used and widely understood system of classification for species at high risk of extinction. The criteria can be applied to any taxonomic unit at or below the species level, though to wild populations only. There are eight possible categories: Data Deficient (DD), Least Concern (LC), Near Threatened (NT), Vulnerable (VU), Endangered (EN), Critically Endangered (CR), Extinct in the Wild (EW) and Extinct (EX). In January 2020, a total of 116,177 species had been assessed for the Red List[9]. A small proportion of assessments (~1%) were assessed using older criteria[see 53] and have not yet been updated. For instance, some species remain categorized as Low Risk/near threatened (LR/nt) which aligns with the Near Threatened (NT) category, and some species remain categorized as Low Risk/conservation dependent (LR/cd), which most closely aligns with Near Threatened (NT) in the most recent criteria[53].

The categories Critically Endangered, Endangered, and Vulnerable are described as globally "threatened", meaning species in these categories are threatened with extinction (the specific details of which are contained in the individual assessments). The listing of a species in a higher extinction risk category implies a higher expectation of extinction, and, over specified

time frames, more taxa listed in a higher category are expected to go extinct than those in a lower category, without effective conservation action.

Species subject to assessments for the Red List are evaluated against the Categories and Criteria[53], which were derived from a wide-ranging review of the risks and threats species face and the diverse life histories species exhibit. The criteria relate to population size reduction, measured over the longer of 10 years or 3 generations (A); restricted geographic range, in the form of either extent of occurrence and/or area or occupancy, combined with other factors (B); small population size and decline (C); a very small or restricted population (D), and quantitative analysis (E)[53].

Threats to species within the Red List are structured hierarchically, and may include, for example, agriculture, invasive species, pollution, and BRU (biological resource use, e.g., hunting of terrestrial animals or collection of terrestrial plants) following a standard classification scheme[38]. In completing assessments, assessors are required to indicate the threats that triggered the listing of the taxon and “Major Threats”, for species listed as EX, EW, CR, EN, VU and NT (but not LC or DD), which should be coded according to the IUCN standardized Threats Classification Scheme[38]. Coding of the timing of the threat (past, on-going or future) and the stresses (how the threat impacts the species) is recommended whereas coding of the scope (is whole or part of the population affected?) and severity (overall decline caused by the threat) is optional (see Supplementary Methods 2.4), but for groups of species in the IUCN Red List Strategic Plan[18], assessors strive to follow the recommended supporting information requirements[54]. This information is useful for demonstrating the means by which threats impact taxa, and for distinguishing past, present and future threats and can be used to calculate a threat impact score[55]. If assessors decide to

record minor threats for species as well, they must record scope and severity for all threat records for the species to enable major and minor threats to distinguished. There is no specific threat code with which to record that harvest for international trade is a threat to species on the Red List (see Methods).

Use is captured as a threat under the Threats Classification Scheme[38] and within the "use and trade module" within individual species assessments, using the Use and Trade Classification Scheme[39]. For this module, it is recommended that assessors code species in terms of whether they are used locally (i.e., at a subsistence level), nationally (i.e., at a domestic level), and/or internationally. Assessors are also asked to code the purpose of use (see Supplementary Methods 2.2). Unlike threats, it is recommended, but not mandatory, for assessors to record information on use and trade in assessments. Once drafted, assessments are peer-reviewed by at least one reviewer to ensure information and data have been interpreted appropriately. Assessments are also directed throughout the process by guidelines[56] and documentation standards[54].

2.2 Queries of the IUCN Red List

Our first query extracted data from the rationale, threats, and use and trade sections of assessments for species selected based on specific criteria including keywords and text strings, and 11 threat codes. The 53 keywords and text strings used are:

"trade"	"international" and ("use")
"transboundary" and ("use")	"trans-boundary" and ("use")
"intercontinental" and ("use")	"inter-continental" and ("use")
"border" and ("use")	"pet" and ("use")
"aquari" and ("use")	"horticultur" and ("use")
"timber" and ("use")	"commercial" and ("use")
"world" and ("use")	"ornamental" and ("use")
"cage bird" and ("use")	"cagebird" and ("use")
"cage-bird" and ("use")	"curio" and ("use")
"medicin" and ("use")	"fisher" and ("use")
"regional" and ("use")	"pharmaceutical" and ("use")
"unsustainabl" and ("use")	"international" and ("utili")
"transboundary" and ("utili")	"trans-boundary" and ("utili")
"intercontinental" and ("utili")	"inter-continental" and ("utili")
"border" and ("utili")	"pet" and ("utili")
"aquari" and ("utili")	"horticultur" and ("utili")
"timber" and ("utili")	"commercial" and ("utili")
"world" and ("utili")	"ornamental" and ("utili")
"cage bird" and ("utili")	"cage-bird" and ("utili")
"cagebird" and ("utili")	"curio" and ("utili")
"medicin" and ("utili")	"fisher" and ("utili")
"regional" and ("utili")	"pharmaceutical" and ("utili")
"unsustainabl" and ("utili")	"export"
"collect"	"enthusiast"
"harvest"	"exploit"
"demand"	"market"
"CITES"	

We included a space in the search string preceding "use", to avoid including assessments containing other words which contain that string of three letters (though this means that occurrences of this word at the start of a paragraph will not have been captured).

The 11 threat codes used are:

5.1.1 – Biological resource use → Hunting & collecting terrestrial animals → Intentional use (species being assessed is the target)

5.1.4 – Biological resource use → Hunting & collecting terrestrial animals → Motivation Unknown/Unrecorded

5.2.1 – Biological resource use → Gathering terrestrial plants → Intentional use (species being assessed is the target)

5.2.4 – Biological resource use → Gathering terrestrial plants → Motivation Unknown/Unrecorded

5.3.1 – Biological resource use → Logging & wood harvesting → Intentional use: subsistence/small scale (species being assessed is the target) [harvest]

5.3.2 – Biological resource use → Logging & wood harvesting → Intentional use: large scale (species being assessed is the target) [harvest]

5.3.5 – Biological resource use → Logging & wood harvesting → Motivation Unknown/Unrecorded

5.4.1 – Biological resource use → Fishing & harvesting aquatic resources → Intentional use: subsistence/small scale (species being assessed is the target) [harvest]

5.4.2 – Biological resource use → Fishing & harvesting aquatic resources → Intentional use: large scale (species being assessed is the target) [harvest]

5.4.4 – Biological resource use → Fishing & harvesting aquatic resources → Unintentional effects: large scale (species being assessed is not the target) [harvest]

5.4.6 – Biological resource use → Fishing & harvesting aquatic resources → Motivation Unknown/Unrecorded

Our second query extracted data on the end uses for which species were coded in the end use table in assessments. The 18 pre-defined purposes of use on the Red List are:

1. Food – human
2. Food – animal
3. Medicine – human & veterinary
4. Poisons
5. Manufacturing chemicals
6. Other chemicals
7. Fuels
8. Fibre
9. Construction or structural materials
10. Wearing apparel, accessories
11. Other households goods
12. Handicrafts, jewellery, etc.
13. Pets/display animals, horticulture
14. Research

15. Sport hunting/specimen collecting
16. Establishing ex-situ production
17. Other (free text)
18. Unknown

2.3 Limitations to IUCN Red List data

Every effort is made to keep the Red List updated. However, there are limitations to these data relating to our analyses.

***Species coverage* -** Not all taxonomic groups have been comprehensively assessed for the Red List meaning that the Red List itself contains biases in its species coverage. However, it comprises the global standard for categorizing species' conservation status, threats, and extinction risk. The Red List has a target of assessing a further 129,000 species by 2030[18] meaning that even with these biases, it comprises the most comprehensive database of threats facing species globally and is ideally placed to inform international policy instruments including CITES.

***Assessments that need updating* -** Every effort is made to keep all Red List assessments updated, including reassessments of species having a target of every four to 10 years, but inevitably some assessments become outdated and are not updated in a timely fashion. As a result, some species have potentially been included in our analyses despite their conservation status having improved and potentially any threat from international trade may have diminished. Equally, species may have been omitted from our analyses when their conservation status has actually deteriorated and the threat from international trade grown, where this is not currently reflected in the Red List. Recent use of the Red List to explore sustainable and unsustainable use of species accounting for these outdated assessments suggests that this will be unlikely to affect our results substantially[34]. However, see Supplementary Discussion 5.1 for how this could be addressed when conveying future iterations of the results to the CITES Parties.

Potential for errors - IUCN seeks to ensure that all Red List assessments are completed to the highest quality, follow documentation standards[53], and undergo peer-review, but it is possible that the Red List contains errors. This has particular relevance in the context of our analyses as information and coding standards for the Red List (e.g., on the local, national, and international use of species) has changed over time meaning species could have been included or omitted from our list of candidate species erroneously. This also applies to the field "no use/trade information for this species" because it is known that assessors may use this field when they are unsure if use or trade involving a particular species occurs, when it ought to be used when it is known or highly likely that the species is used and/or traded but further information on use and/or trade is not available[39].

Language – For the purposes of this research we queried the Red List in English. However, the Red List also accepts assessments in French, Portuguese and Spanish. At the end of 2020 there were a small number of assessments on the Red List in these languages: French (54 asssessments), Portuguese (1,005 assessments) and Spanish (1,721 assessments). As such, a further limitation to this study is that it is possible that there are assessments in these languages in which species have been determined to be threatened by international trade, but which have not been included in our dataset. Future analyses using the methods presented here would benefit from conducting queries of the Red List in these additional languages.

2.4 Species categorisation

Automated coding

We automatically coded species to assign them to a category - Likely, Unlikely or Insufficient information - based on the “use and trade” and “is international trade a significant driver of threat” fields, and the relevance of use-related threat codes (Extended Data Fig. 4). This was undertaken in R (Version 4.0.3[41]) and used to identify species that could clearly be assigned a category (e.g., Likely) based on one or two data fields only. The three bases for automated coding were:

1. International trade is considered a significant driver of threat to species by Red List assessors (coded Likely): these assessments had the “is international trade a significant driver of threat” field marked “yes”.

2. Animals or fungi where the only use-related threats were associated with plants (coded Unlikely): these animal or fungi species were selected in the initial queries (Supplementary Methods 2.2) due to plant-related threat codes only (i.e., threat codes within 5.2 and 5.3 [e.g., animal/fungi species impacted by logging]). They also did not include any of the text strings listed in Supplementary Methods 2.2 other than those referring to “timber” or “horticultur” (i.e., not direct threats to animals or fungi) in the rationale, threats, or use and trade sections of assessments.

3. Red List assessors did not consider use/trade to be occurring and/or a threat (coded Unlikely) or noted that there was a lack of information on use and/or trade (coded Insufficient information). For these species text strings in the “use and trade” field indicated that either:

(a) use and/or trade were not occurring or unlikely to be a threat (328 text strings). Text string searches comprised the following 13 strings:

"not targeted specifically"	"none known"
"no known human use"	"no use* know*"
"no* know* use"	"no report**** use"
"no know* record**** use"	"no know* report**** use"
"no known removal trade"	"not found commercial pet trade"
"no way use* trad*"	"trade isnt apparent"
"trade unrecorded"	

as well as 315 text strings based on prefixing 9 key trade strings ("trad***", "harvest***", "export*****", "exploit*****", "utilis*****", "trapp****", "collect****", "use" and "international trad") with each of the following 35 prefix strings:

"no"	"not known"
"not"	"not found"
"no* current**"	"current** no*"
"no evidence"	"no evidence current"
"no* direct**"	"no existing"
"neither"	"appears not"
"no information suggests"	"not subject"
"not thought use"	"not thought"
"no* document**"	"no* apparent"
"no known specific"	"no* known current**"
"no* know******"	"no documentation"
"no* report**"	"no* record**"
"no record****"	"no report****"
"no* re****ed***"	"no report**** know*"
"never re****ed"	"not believed"
"no* thought"	"doesnt appear"
"doesnt appear use*"	"unlikely"
"no* significant** use*"	

(e.g., "no trade***", "not known trade***", "no harvest***")

or (b) there was insufficient information about use and/or trade (55 text strings):

"no use* information"	"no trad*** information"
"no use*trade information"	"no* known use"
"no* known whether use"	"no information use*"
"no information trade"	"no information regarding use*"
"no information regarding trade"	"no information regarding**** trade"
"no information found use*"	"no information found trade"
"no information known about use"	"no information suggest* trade"
"no specific use*trade information"	"no specific trade information"
"no information**** available"	"no information about ****use"
"no current information use*"	"no current information trade"

“no data particular use*”	“no data particular trad*”
“no known information use”	“no known information trade”
“no known information about use”	“no known information about trade”
“more information needed”	“more information required”
“no available data”	“no information whether harvest”
“information use*trade not available”	“information isnt available”
“no specific data available”	“no* available use*trade”
“use*trad*** information not available”	“trade status unknown”
“trade*use unknown”	“use*trade unknown”
“unknown use*trade”	“unknown whether use”
“unknown whether trade”	“use* unknown”
“trad*** unknown”	“unknown trade”
“information** unknown”	“trade information unknown”
“use* specifically unknown”	“trad*** specifically unknown”
“information regarding trade*use isnt known”	“information** missing”
“trade unrecord**”	“no report** information”
“no report** use* information”	“no record** information use”
“no* re****** information trade”	

To maximise opportunities for string matching within the free text of the “use and trade” field, characters were first standardised to lower case, all characters other than letters and spaces were removed, common abbreviations were standardised, and 34 stopwords (“a”, “also”, “an”, “and”, “any”, “are”, “as”, “be”, “been”, “being”, “by”, “for”, “from”, “has”, “have”, “if”, “in”, “is”, “it”, “of”, “or”, “still”, “that”, “the”, “there”, “this”, “to”, “were”, “where”, “which”, “with”, “would”, “species”, “wild”) were removed.

Species that could not be automatically coded based on these parameters (i.e., because more detailed or nuanced information and interpretation was necessary) were manually coded.

Manual coding

This entailed reading all the available information for each assessment in our MS Excel database—rationale, threats, and use and trade text fields, threat codes, scale of use codes, purpose of use codes, “no use/trade information on this species”, and “is international trade a significant driver of threat”—and carefully categorizing species aided by a decision tree

(Extended Data Fig. 5). For some species this was simple because a relevant threat code (e.g., 5.1.1) had been applied in the assessment and the accompanying narrative in the rationale, threats and/or use and trade text field stated that international trade was a threat to the species, in which case the species was coded as Likely to be threatened by international trade. Similarly, for some assessments no relevant threat code was present and the accompanying narrative stated that use and/or trade were not a threat to the species; these species were coded Unlikely to be threatened by international trade. For other species, it was less clear and careful interpretation of the available information was needed in order to determine that the species is used and/or traded, that use and/or trade is a threat to the species, and that the use and/or trade comprising the threat to the species is to some extent international (Extended Data Fig. 5).

A note on the timing of threats. Where major threats to species are coded in assessments, it is recommended that information on the timing of the threats and stresses be included whereas coding of the scope and severity of the threats is optional (Supplementary Methods 2.4). As the purpose of this study is to determine categorically whether there is evidence that species are threatened by international trade, or not, using available information in assessments—and recognising that information on the severity and scope of threats is often not included in assessments[34]—we considered the temporal qualification of threats but not information on scope, severity or stresses of coded threats. Specifically, we considered threats to be current unless they were qualified as “past (unlikely to return)”, which we interpreted as no longer being present; and “future” which we interpreted to be a potential future threat (Extended Data Fig. 5). In other words, we considered threats regardless of whether or how scope or severity (and stresses) had been coded.

Examples of coded species are below.

Sun bear *Helarctos malaynus* (Likely) – The species has relevant threat codes applied (5.1.1, 5.3.1 and 5.3.2) and the supporting text indicates that the species is used and traded and that this is a threat to the species. Some of this use and trade is international which is documented in the narrative text, international end use is indicated by the scale of use, and the international end uses “Food-human” and “Medicine – human & veterinary” also correspond to the narrative text regarding threats.

***Paphiopedilum dianthum* (Likely)** – This orchid species has a relevant threat code applied (5.2.1) and the narrative text indicates that collection for international trade is a threat. International use is also indicated by the international end use field being coded “yes” and the purpose of international use “Pets/display animals, horticulture” corresponds with the narrative on threat.

Aurelio’s rock lizard *Iberolacerta aurelio* (Insufficient information) – This species has a relevant threat code applied (5.1.1) and the scale of use field indicates that the species is used internationally, which the end use field indicates is for research purposes. However, the threat from intentional use, described as “collection”, is qualified as being “possible” in the threats field.

***Dypsis ambanjae* (Insufficient information) –** This palm species is in international trade which is indicated in the use and trade narrative text, and by the scale of use international field being coded “yes”, and the end use field indicates that the international end use is for “Pets/display animals, horticulture”. However, there is no evidence that use and trade is a

threat to the species anywhere in the supporting narrative and no relevant threat codes have been applied.

***Contomastix vittate* (Unlikely) –** This reptile species has a relevant threat code applied (5.1.1) but the narrative text indicates that the species is used only locally for religious customs. The scale of use fields indicate that use only occurs at the subsistence level.

***Elaphoglossum sprucei* (Unlikely)** – This species of fern has no relevant threat codes applied, there is no evidence that use or trade takes place from the narrative text, there is no information on the scale of use, and no end uses have been documented. This species was included in the candidate list of species because the assessment rationale refers to collection of the type specimen.

2.5 Estimates of the proportion of species Likely threatened by international trade

Within our dataset there are 6,583 species that were coded Insufficient information when applying our criteria, i.e., there is insufficient information in assessments to determine whether these species are, or are not, threatened by international trade. Theoretically, a fraction of these species, or all of them, may be threatened by international trade. To account for this uncertainty, we follow previous studies[44,45] that use Red List data and present three values for species Likely threatened by international trade as a proportion of (i) globally threatened and Near Threatened species and (ii) species with coded BRU threats in our dataset: lower bound, mid-point, and upper bound.

Proportion of globally threatened and Near Threatened species

- Lower bound: percentage of species coded Likely (number of species coded Likely divided by the total number of globally threatened and Near Threatened species). This assumes that none of the species coded Insufficient information are Likely threatened by international trade. This should be interpreted as a minimum estimate.

- Mid-point: percentage of species coded Likely among those for which the likelihood of being threatened by international trade could be determined (number of species coded Likely divided by the number of species coded Likely and Unlikely). This assumes that species coded Insufficient information have the same fraction of species coded Likely as among species coded Likely and Unlikely. This demonstrates that the true value lies somewhere between the lower and upper bound.

- Upper bound: proportion of species coded Likely or Insufficient information (number of species coded Likely or Insufficient information divided by the total number of globally

threatened and Near Threatened species). This assumes that all of the species coded Insufficient information are Likely threatened by international trade. This is the most pessimistic estimate.

Proportion of species with coded BRU threats

- Lower bound: percentage of species with coded BRU threats and coded Likely (number of species with coded BRU threats and coded Likely divided by the total number of species with coded BRU threats in our dataset). This assumes that none of the species with coded BRU threats in our dataset and coded Insufficient information are Likely threatened by international trade. This should be interpreted as a minimum estimate.

- Mid-point: percentage of species with coded BRU threats and coded Likely among those for which the likelihood of being threatened by international trade could be determined (number of species with coded BRU threats and coded Likely divided by the number of species with coded BRU threats and coded Likely and Unlikely). This assumes that species with coded BRU threats and coded Insufficient information have the same fraction of species coded Likely as among species with coded BRU threats and coded Likely and Unlikely. This demonstrates that the true value lies somewhere between the lower and upper bound.

- Upper bound: percentage of species with coded BRU threats and coded Likely or Insufficient information (number of species with coded BRU threats and coded Likely or Insufficient information divided by the number of species with coded BRU threats). This assumes that all of the species with coded BRU threats and coded Insufficient information are Likely threatened by international trade. This is the most pessimistic estimate.

2.6 The IUCN Red List Categories and Criteria and the CITES listing criteria

There are similarities and differences between the IUCN Red List Categories and Criteria[53] and the CITES listing criteria[7] with both sets of criteria having a shared history dating back to the early 1990s. However, the finalization of the two sets of original criteria was independent and both the CITES Parties and IUCN adopted new criteria independently in 1994.

In 2001, IUCN revised its own objective and quantitative criteria (introducing changed and new explicit thresholds), which increased the divergence from the CITES listing criteria. The Red List Categories and Criteria are applicable to a wide range of taxa so that all wild species can be classified into one of a number of categories of extinction risk using available information (Supplementary Methods 2.1). Critically, while the Red List captures information on threats and use and trade, there is no direct link between (i) threats and category thresholds and (ii) end uses, scale of use, trade volumes and dynamics, and the impact of harvest for trade (at all scales), beyond information in the relevant text fields (Methods, Supplementary Methods 2.1). This differs to CITES.

The CITES listing criteria have been updated since 1994, with the latest version of the criteria adopted at CITES CoP17 in 2016[7]. In contrast to the Red List Categories and Criteria, the CITES listing criteria take into account both biological and trade considerations explicitly. Within proposals to amend the CITES Appendices, Parties must show that species are, to a greater or lesser extent, not only threatened, but that international trade is occurring, and that the species are negatively affected (actually or potentially) by international trade. There are different criteria that must be met for species to be listed in the various Appendices, with a higher threshold required to meet the Appendix I criteria[7].

An important aspect of the CITES listing criteria is the precautionary approach. Within the listing criteria[7] it is stated: “by virtue of the precautionary approach and in case of uncertainty regarding the status of a species or the impact of trade on the conservation of a species, the Parties shall act in the best interest of the conservation of the species concerned and, when considering proposals to amend Appendix I or II, adopt measures that are proportionate to the anticipated risks to the species”. This provides Parties with discretion to handle imperfect or incomplete data in a way that they consider to be most beneficial to the species. While Red List assessments may be completed in a precautionary manner[56], the implications are notably different; species could be assessed as meeting a higher threat category, or in the real absence of data, be assessed as DD. Assessors are also required to state their risk tolerance.

In addition, the CITES listing criteria provide a broad framework for meeting the biological and trade criteria, rather than requiring specific numerical thresholds to be met, as is the case for the Red List. For instance, Annex 5 of the listing criteria[7] states that “it is impossible to give numerical values that are applicable to all taxa because of differences in their biology”. This contrasts with the Red List, which requires that set numerical thresholds and associated subcriteria be met in order for a species to be listed in a particular threat category.

The CITES listing criteria are also designed to fit the framework of the Convention and be sufficiently practicable and feasible for governments to bring proposals to the table to amend the Appendices. Thus, there is great flexibility and discretion by the Parties regarding interpretation of some aspects of the criteria to ensure that the absence of perfect data does not prevent trade measures being adopted for species. There is arguably less flexibility to assessing species for the Red List where assessors must follow and adhere to set guidelines and documentation standards prior to assessments being peer reviewed.

Regarding CITES, there is also the added layer of the listing process itself, which means that proposals do not automatically get adopted even if all the criteria are deemed by the Parties to be met; they must be agreed and adopted by the CoP, and this decision may be made for one or more of biological, economic, and/or political reasons, among others[57]. In some cases, the Parties may decide that a species would benefit from greater trade regulation, but in others, they may decide that it may not, or that there are risks to revising trade measures for a species and thus come to the decision not to adopt a particular proposal. This will depend on the species, the geography of trade, and interactions among social, economic, biological and governance factors[2] (see also Supplementary Discussion 5.1).

2.7 Aligning taxonomies between the IUCN Red List and CITES

There was a need to align taxonomies within this study because those used by the Red List and CITES differ. Taxonomy on the Red List is the responsibility of the IUCN Species Survival Commission (SSC) Red List Authorities (RLAs), which complete assessments for their focal species. Specifically, RLAs are “responsible for determining and agreeing with the IUCN Red List Unit the taxonomic treatment and nomenclature used on the IUCN Red List for the species in that group” following the Rules of Procedure for assessments[58]. In the case of CITES, the species nomenclature is set by the Parties according to adopted standard nomenclatural references for species and groups as outlined in CITES Resolution Conf. 12.11 (Rev. CoP18) on *Standard nomenclature*[59].

2.8 Retrospective automated coding

Retrospective coding of Actinopterygii and Amphibia species

For the mechanism presented in this article to be repeatable in the future as new or updated Red List assessments are published (e.g., to explore temporal trends in species Likely threatened by international trade, or to identify species that may be newly/no longer considered Likely threatened by international trade), ongoing manual assessment is not practical. This is due to the sheer number of species on the Red List, which is increasing, and the coding time that would be needed. As such, we developed code in R (Version 4.0.3)[41] to recreate the manual coding process detailed in Extended Data Fig. 5. This was tested against two animal classes, Actinopterygii and Amphibia, containing 1,187 and 329 assessments that were manually coded respectively. We then tested the automated coding against the initial coding for the Actinopterygii and Amphibia species in our dataset (3,623 species). For both of these tests we used Fleiss' Kappa in SPSS v.28 to test the reliability of coding and whether agreement was higher than would have been expected by chance.

Species were assessed following the steps of the decision tree (Extended Data Fig. 5) using combinations of the following categorical data fields: threat code (Supplementary Methods 2.2), purpose of use (Supplementary Methods 2.2), and scale of use (i.e., subsistence, national or international), and keyword string searches in three free-text fields: rationale, threats, and use and trade. To maximise opportunities for string matching within these free-text fields, characters were first standardised to lower case, all characters other than letters and spaces were removed, common abbreviations were standardised and 48 stopwords ("a", "about", "also", "an", "and", "any", "are", "as", "be", "been", "being", "but", "by", "can", "for", "from", "has", "have", "if", "in", "includ***", "into", "is", "it", "its, "much", "of", "on", "or", "so", "still", "such", "than", "that", "the", "there", "therefore", "these", "this", "to",

“were”, “where”, “which”, “with”, “within”, “would”, “species”, “wild”) were removed. Code, including full lists of all keyword strings, is available via GitHub (link).

Species were assessed following the sequential steps below, which align with those of the decision tree (Extended Data Fig. 5). Once species had been classified as Likely, Unlikely or Insufficient Information, they were not assessed in any subsequent steps.

Step 1. Use and/or trade was considered to take place if one or more of the following criteria applied to the species:

i. Use and trade were reported at any scale of use (i.e., subsistence, national and/or international fields were ticked).

ii. Species had at least one relevant biological resource use threat code applied (relevant threat codes with 5.1 and 5.4 for animals, and 5.2 and 5.3 for plants, see Supplementary Methods 2.2 for threat codes included in these analyses).

iii. Relevant trade keyword strings indicating that use and/or trade occurs (n=180 strings) were present in the free text of the rationale, threats, and/or use and trade fields.

Where there was NO evidence of use and/or trade taking place, species were assessed under the criteria of **step 2a.**

Where there WAS evidence of use and/or trade based on the step 1 rules, species were assessed under the criteria of **step 2b.**

Step 2a. Use and/or trade was considered a potential future threat if relevant keyword strings indicating that use/trade was a potential future threat (n=181 strings) were present in the free text of the rationale, threats, and/or use and trade fields.

Where there was NO evidence of use and/or trade as a potential future threat, species assessed under step 2a were classified as **Unlikely**.
Where there WAS evidence of use and/or trade being a potential future threat, species were classified as **Insufficient information**.

Step 2b. Use and/or trade was not considered international if one or more of the following criteria applied to the species:

i. Use and trade were reported as occurring at the local and/or national level but NOT at the international level (based on subsistence, national and international use fields).
ii. Relevant keyword strings indicating that there was no international trade in the species (n=558 strings) were present in the free text of the rationale, threats, and/or use and trade fields.

Where there was NO evidence that use and/or trade was not international, species were then assessed under the criteria of **step 3.**
Where there WAS evidence that use and/or trade was not international, species assessed under step 2b were classified as **Unlikely**.

Step 3. Use and/or trade was not considered a threat if relevant keyword strings indicating that use and trade were not a threat to the species (n=218 strings) were present in the free text of the rationale, threats, and/or use and trade fields.

Where there was NO evidence that use and/or trade was not a threat, species were then assessed under the criteria of **step 4.**
Where there WAS evidence that use and/or trade was not a threat, species assessed under step 3 were classified as **Unlikely**.

Step 4. Use and/or trade was considered a threat if one or more of the following criteria applied to the species:

i. Species had at least one relevant biological resource use threat code applied (relevant threat codes with 5.1 and 5.4 for animals, and 5.2 and 5.3 for plants, see Supplementary Methods 2.2 for threat codes included in these analyses), excluding those with timings of "future" or "past, unlikely to return".
ii. Relevant keyword strings indicating use and/or trade was a threat (n=2724 strings) were present in the free text of the rationale, threats, and/or use and trade fields.

Where there was NO evidence that use and/or trade was a threat, species were classified as **Insufficient information**.
Where there WAS evidence that use and/or trade was a threat, species assessed under step 4 were then assessed under the criteria of **step 5**.

Step 5. The use and/or trade comprising the threat in step 4 was considered to some extent international if one or more of the following criteria applied to the species:

i. Relevant keyword strings indicating that trade was international (n=138 strings) were present in the free text of the rationale, threats, and/or use and trade fields.

ii. Specific end uses (“Food – human”, “Handicrafts, jewellery, etc.”, “Medicine – human & veterinary”, “Pets/display animals, horticulture”, “Sport hunting/specimen collecting”) were considered to occur at the international scale (i.e., international field was ticked) along with end-use specific keyword strings indicating that use and/or trade was a threat (food: n=547 strings; handicrafts: n=360 strings; medicine: n=181 strings; pets: n=1445 strings; sport/collecting: n=90 strings) in the **threats, rationale, and/or use and trade** fields. Since this is end-use specific, this criterion was considered to override the Insufficient information classification under step 4.

iii. Specific end uses (“Food – human”, “Handicrafts, jewellery, etc.”, “Medicine – human & veterinary”, “Pets/display animals, horticulture”, “Sport hunting/specimen collecting”) were considered to occur at the international scale (i.e., international field was ticked) along with end-use specific keyword strings indicating that use and/or trade occurred (food: n=40 strings; handicrafts: n=24 strings; medicine: n=12 strings; pets: n=102 strings; sport/collecting: n=6 strings) in **threats**

See Supplementary Table 3 and Supplementary Results 4.3 for results.

Classification of new and updated Red List assessments

This study used data from Red List version 2020-1 but since its publication there have been five updates to the Red List, the most recent version (2021-3) was published on 9 December 2021. To demonstrate the utility of automatically coding species with new assessments and to

identify whether any new Actinopterygii or Amphibia species are Likely to be threatened by international trade on subsequent versions of the Red List, the methods described in Supplementary Methods 2.2–2.4 and above (this section) were re-run for Actinopterygii and Amphibia (1,947 reassessed or newly assessed species) based on data downloaded from Red List version 2021-2 on 6 September 2021. See Supplementary Tables 3–5 and Supplementary Results 4.3 for the results.

Classification of broader taxonomic groups

The advanced automated coding process outlined above was developed using two animal classes (Actinopterygii and Amphibia) to test the degree to which the manual coding could be replicated using a systematic set of hard coded rules and keyword strings. This is necessary for this process to be repeated (e.g., on new or updated assessments) in the future. Consequently, a number of the more specific keyword strings used have been tailored to these taxonomic groups (e.g., fishing-related keyword strings relating to Actinopterygii harvesting). To test the broader utility of the automated approach and current keyword strings, the process was subsequently run for all animals (kingdom Animalia) in our dataset. We used Fleiss' Kappa in SPSS v.28 to test the reliability of coding between manual and automated coding and whether agreement was higher than would have been expected by chance. See Supplementary Table 6 and Supplementary Results 4.3 for the results.

3. Supplementary Tables and Figures

Supplementary Table 1 | Species categorised as Likely threatened by international trade and corresponding Red List category and CITES Appendix. Taxonomic information is based on the nomenaclature used by the IUCN Red List. Species not listed in the CITES Appendices are indicated with NC (non-CITES).

**Class**	**Family**	**Species**	**Red List category**	**CITES Appendix**
ACTINOPTERYGII	ACIPENSERIDAE	*Acipenser baerii*	EN	II
ACTINOPTERYGII	ACIPENSERIDAE	*Acipenser dabryanus*	CR	II
ACTINOPTERYGII	ACIPENSERIDAE	*Acipenser gueldenstaedtii*	CR	II
ACTINOPTERYGII	ACIPENSERIDAE	*Acipenser mikadoi*	CR	II
ACTINOPTERYGII	ACIPENSERIDAE	*Acipenser nudiventris*	CR	II
ACTINOPTERYGII	ACIPENSERIDAE	*Acipenser persicus*	CR	II
ACTINOPTERYGII	ACIPENSERIDAE	*Acipenser ruthenus*	VU	II
ACTINOPTERYGII	ACIPENSERIDAE	*Acipenser schrenckii*	CR	II
ACTINOPTERYGII	ACIPENSERIDAE	*Acipenser sinensis*	CR	II
ACTINOPTERYGII	ACIPENSERIDAE	*Acipenser stellatus*	CR	II
ACTINOPTERYGII	ACIPENSERIDAE	*Huso dauricus*	CR	II
ACTINOPTERYGII	ACIPENSERIDAE	*Huso huso*	CR	II
ACTINOPTERYGII	ANGUILLIDAE	*Anguilla anguilla*	CR	II
ACTINOPTERYGII	ANGUILLIDAE	*Anguilla australis*	NT	NC
ACTINOPTERYGII	ANGUILLIDAE	*Anguilla bengalensis*	NT	NC
ACTINOPTERYGII	ANGUILLIDAE	*Anguilla bicolor*	NT	NC
ACTINOPTERYGII	ANGUILLIDAE	*Anguilla dieffenbachii*	EN	NC
ACTINOPTERYGII	ANGUILLIDAE	*Anguilla japonica*	EN	NC
ACTINOPTERYGII	ANGUILLIDAE	*Anguilla luzonensis*	VU	NC
ACTINOPTERYGII	ANGUILLIDAE	*Anguilla mossambica*	NT	NC
ACTINOPTERYGII	ANGUILLIDAE	*Anguilla rostrata*	EN	NC
ACTINOPTERYGII	ATHERINOPSIDAE	*Chirostoma lucius*	EN	NC
ACTINOPTERYGII	ATHERINOPSIDAE	*Chirostoma sphyraena*	EN	NC
ACTINOPTERYGII	MELANOTAENIIDAE	*Melanotaenia boesemani*	EN	NC
ACTINOPTERYGII	TELMATHERINIDAE	*Marosatherina ladigesi*	VU	NC
ACTINOPTERYGII	SYNODONTIDAE	*Harpadon nehereus*	NT	NC
ACTINOPTERYGII	ADRIANICHTHYIDAE	*Oryzias woworae*	EN	NC
ACTINOPTERYGII	CHARACIDAE	*Astyanax jordani*	EN	NC
ACTINOPTERYGII	LEBIASINIDAE	*Nannostomus mortenthaleri*	CR	NC
ACTINOPTERYGII	CLUPEIDAE	*Clupeonella engrauliformis*	EN	NC
ACTINOPTERYGII	CLUPEIDAE	*Clupeonella grimmi*	EN	NC
ACTINOPTERYGII	CLUPEIDAE	*Clupeonella muhlisi*	EN	NC
ACTINOPTERYGII	CLUPEIDAE	*Sardinella lemuru*	NT	NC
ACTINOPTERYGII	CLUPEIDAE	*Tenualosa macrura*	NT	NC
ACTINOPTERYGII	CLUPEIDAE	*Tenualosa toli*	VU	NC

Class	Family	Species	Red List category	CITES Appendix
ACTINOPTERYGII	BALITORIDAE	*Gastromyzon aequabilis*	NT	NC
ACTINOPTERYGII	BALITORIDAE	*Gastromyzon auronigrus*	NT	NC
ACTINOPTERYGII	BALITORIDAE	*Gastromyzon borneensis*	NT	NC
ACTINOPTERYGII	BALITORIDAE	*Gastromyzon ctenocephalus*	NT	NC
ACTINOPTERYGII	BALITORIDAE	*Gastromyzon extrorsus*	NT	NC
ACTINOPTERYGII	BALITORIDAE	*Gastromyzon farragus*	VU	NC
ACTINOPTERYGII	BALITORIDAE	*Gastromyzon ingeri*	NT	NC
ACTINOPTERYGII	BALITORIDAE	*Gastromyzon ocellatus*	NT	NC
ACTINOPTERYGII	BALITORIDAE	*Gastromyzon pariclavis*	NT	NC
ACTINOPTERYGII	BALITORIDAE	*Gastromyzon scitulus*	VU	NC
ACTINOPTERYGII	BALITORIDAE	*Gastromyzon spectabilis*	NT	NC
ACTINOPTERYGII	BALITORIDAE	*Gastromyzon umbrus*	NT	NC
ACTINOPTERYGII	BALITORIDAE	*Gastromyzon zebrinus*	NT	NC
ACTINOPTERYGII	BALITORIDAE	*Longischistura striatus*	EN	NC
ACTINOPTERYGII	BALITORIDAE	*Nemacheilus troglocataractus*	CR	NC
ACTINOPTERYGII	BALITORIDAE	*Sewellia albisuera*	CR	NC
ACTINOPTERYGII	BALITORIDAE	*Sewellia marmorata*	EN	NC
ACTINOPTERYGII	BALITORIDAE	*Travancoria elongata*	EN	NC
ACTINOPTERYGII	COBITIDAE	*Leptobotia elongata*	VU	NC
ACTINOPTERYGII	COBITIDAE	*Syncrossus beauforti*	NT	NC
ACTINOPTERYGII	COBITIDAE	*Syncrossus berdmorei*	NT	NC
ACTINOPTERYGII	COBITIDAE	*Yasuhikotakia nigrolineata*	VU	NC
ACTINOPTERYGII	CYPRINIDAE	*Alburnus sarmaticus*	EN	NC
ACTINOPTERYGII	CYPRINIDAE	*Alburnus schischkovi*	EN	NC
ACTINOPTERYGII	CYPRINIDAE	*Barbodes dunckeri*	EN	NC
ACTINOPTERYGII	CYPRINIDAE	*Cirrhinus cirrhosus*	VU	NC
ACTINOPTERYGII	CYPRINIDAE	*Devario auropurpureus*	EN	NC
ACTINOPTERYGII	CYPRINIDAE	*Devario pathirana*	EN	NC
ACTINOPTERYGII	CYPRINIDAE	*Garra ceylonensis*	NT	NC
ACTINOPTERYGII	CYPRINIDAE	*Hypselobarbus curmuca*	EN	NC
ACTINOPTERYGII	CYPRINIDAE	*Leptobarbus melanopterus*	NT	NC
ACTINOPTERYGII	CYPRINIDAE	*Microrasbora rubescens*	EN	NC
ACTINOPTERYGII	CYPRINIDAE	*Paedocypris micromegethes*	VU	NC
ACTINOPTERYGII	CYPRINIDAE	*Paedocypris progenetica*	NT	NC
ACTINOPTERYGII	CYPRINIDAE	*Pectenocypris micromysticetus*	NT	NC
ACTINOPTERYGII	CYPRINIDAE	*Pethia cumingii*	EN	NC
ACTINOPTERYGII	CYPRINIDAE	*Pethia nigrofasciata*	VU	NC
ACTINOPTERYGII	CYPRINIDAE	*Pethia reval*	EN	NC
ACTINOPTERYGII	CYPRINIDAE	*Probarbus jullieni*	CR	I
ACTINOPTERYGII	CYPRINIDAE	*Probarbus labeamajor*	EN	NC
ACTINOPTERYGII	CYPRINIDAE	*Puntius titteya*	VU	NC
ACTINOPTERYGII	CYPRINIDAE	*Rasbora patrickyapi*	NT	NC
ACTINOPTERYGII	CYPRINIDAE	*Rasboroides pallidus*	EN	NC
ACTINOPTERYGII	CYPRINIDAE	*Rasboroides vaterifloris*	EN	NC

Class	Family	Species	Red List category	CITES Appendix
ACTINOPTERYGII	CYPRINIDAE	*Rhodeus atremius*	NT	NC
ACTINOPTERYGII	CYPRINIDAE	*Sahyadria chalakkudiensis*	EN	NC
ACTINOPTERYGII	CYPRINIDAE	*Sahyadria denisonii*	EN	NC
ACTINOPTERYGII	CYPRINIDAE	*Sundadanio atomus*	EN	NC
ACTINOPTERYGII	CYPRINIDAE	*Sundadanio axelrodi*	VU	NC
ACTINOPTERYGII	CYPRINIDAE	*Sundadanio echinus*	VU	NC
ACTINOPTERYGII	CYPRINIDAE	*Sundadanio gargula*	VU	NC
ACTINOPTERYGII	CYPRINIDAE	*Sundadanio goblinus*	EN	NC
ACTINOPTERYGII	CYPRINIDAE	*Sundadanio margarition*	NT	NC
ACTINOPTERYGII	CYPRINIDAE	*Sundadanio rubellus*	NT	NC
ACTINOPTERYGII	CYPRINIDAE	*Systomus pleurotaenia*	VU	NC
ACTINOPTERYGII	APLOCHEILIDAE	*Aplocheilus werneri*	EN	NC
ACTINOPTERYGII	CYPRINODONTIDAE	*Orestias pentlandii*	VU	NC
ACTINOPTERYGII	NOTHOBRANCHIIDAE	*Aphyosemion bivittatum*	VU	NC
ACTINOPTERYGII	NOTHOBRANCHIIDAE	*Epiplatys longiventralis*	VU	NC
ACTINOPTERYGII	NOTHOBRANCHIIDAE	*Epiplatys ruhkopfi*	CR	NC
ACTINOPTERYGII	NOTHOBRANCHIIDAE	*Fundulopanchax arnoldi*	EN	NC
ACTINOPTERYGII	NOTHOBRANCHIIDAE	*Scriptaphyosemion bertholdi*	EN	NC
ACTINOPTERYGII	NOTHOBRANCHIIDAE	*Scriptaphyosemion liberiense*	NT	NC
ACTINOPTERYGII	RIVULIDAE	*Anablepsoides speciosus*	CR	NC
ACTINOPTERYGII	RIVULIDAE	*Austrolebias viarius*	EN	NC
ACTINOPTERYGII	MEGALOPIDAE	*Megalops atlanticus*	VU	NC
ACTINOPTERYGII	MACROURIDAE	*Coryphaenoides rupestris*	CR	NC
ACTINOPTERYGII	MERLUCCIIDAE	*Merluccius bilinearis*	NT	NC
ACTINOPTERYGII	MERLUCCIIDAE	*Merluccius senegalensis*	EN	NC
ACTINOPTERYGII	INDOSTOMIDAE	*Indostomus crocodilus*	VU	NC
ACTINOPTERYGII	LOPHIIDAE	*Lophius vomerinus*	NT	NC
ACTINOPTERYGII	NOTOPTERIDAE	*Chitala blanci*	NT	NC
ACTINOPTERYGII	OSTEOGLOSSIDAE	*Scleropages formosus*	EN	I
ACTINOPTERYGII	AMBASSIDAE	*Parambassis lala*	NT	NC
ACTINOPTERYGII	APOGONIDAE	*Pterapogon kauderni*	EN	NC
ACTINOPTERYGII	BLENNIIDAE	*Ecsenius tricolor*	VU	NC
ACTINOPTERYGII	CARANGIDAE	*Trachurus japonicus*	NT	NC
ACTINOPTERYGII	CARANGIDAE	*Trachurus trachurus*	VU	NC
ACTINOPTERYGII	CHAETODONTIDAE	*Chaetodon trifascialis*	NT	NC
ACTINOPTERYGII	CHANNIDAE	*Channa bleheri*	NT	NC
ACTINOPTERYGII	CICHLIDAE	*Aulonocara baenschi*	CR	NC
ACTINOPTERYGII	CICHLIDAE	*Aulonocara ethelwynnae*	NT	NC
ACTINOPTERYGII	CICHLIDAE	*Aulonocara kandeense*	CR	NC
ACTINOPTERYGII	CICHLIDAE	*Aulonocara maylandi*	CR	NC
ACTINOPTERYGII	CICHLIDAE	*Chindongo demasoni*	VU	NC
ACTINOPTERYGII	CICHLIDAE	*Chindongo saulosi*	CR	NC
ACTINOPTERYGII	CICHLIDAE	*Copadichromis azureus*	NT	NC
ACTINOPTERYGII	CICHLIDAE	*Etroplus canarensis*	EN	NC
ACTINOPTERYGII	CICHLIDAE	*Iodotropheus sprengerae*	NT	NC

Class	Family	Species	Red List category	CITES Appendix
ACTINOPTERYGII	CICHLIDAE	*Labidochromis joanjohnsonae*	NT	NC
ACTINOPTERYGII	CICHLIDAE	*Melanochromis chipokae*	CR	NC
ACTINOPTERYGII	CICHLIDAE	*Melanochromis lepidiadaptes*	CR	NC
ACTINOPTERYGII	CICHLIDAE	*Melanochromis mossambiquensis*	NT	NC
ACTINOPTERYGII	CICHLIDAE	*Metriaclima benetos*	NT	NC
ACTINOPTERYGII	CICHLIDAE	*Metriaclima chrysomallos*	NT	NC
ACTINOPTERYGII	CICHLIDAE	*Metriaclima cyneusmarginatum*	NT	NC
ACTINOPTERYGII	CICHLIDAE	*Metriaclima glaucos*	NT	NC
ACTINOPTERYGII	CICHLIDAE	*Metriaclima greshakei*	NT	NC
ACTINOPTERYGII	CICHLIDAE	*Metriaclima koningsi*	CR	NC
ACTINOPTERYGII	CICHLIDAE	*Metriaclima usisyae*	CR	NC
ACTINOPTERYGII	CICHLIDAE	*Metriaclima xanstomachus*	NT	NC
ACTINOPTERYGII	CICHLIDAE	*Nimbochromis fuscotaeniatus*	VU	NC
ACTINOPTERYGII	CICHLIDAE	*Petrotilapia xanthos*	NT	NC
ACTINOPTERYGII	CICHLIDAE	*Placidochromis phenochilus*	EN	NC
ACTINOPTERYGII	CICHLIDAE	*Pseudotropheus cyaneorhabdos*	CR	NC
ACTINOPTERYGII	CICHLIDAE	*Pseudotropheus galanos*	NT	NC
ACTINOPTERYGII	CICHLIDAE	*Pseudotropheus interruptus*	NT	NC
ACTINOPTERYGII	CICHLIDAE	*Pungu maclareni*	CR	NC
ACTINOPTERYGII	CICHLIDAE	*Rhamphochromis longiceps*	VU	NC
ACTINOPTERYGII	CICHLIDAE	*Serranochromis robustus*	CR	NC
ACTINOPTERYGII	CICHLIDAE	*Trematocranus microstoma*	EN	NC
ACTINOPTERYGII	DATNIOIDIDAE	*Datnioides pulcher*	CR	NC
ACTINOPTERYGII	DATNIOIDIDAE	*Datnioides undecimradiatus*	VU	NC
ACTINOPTERYGII	EPINEPHELIDAE	*Epinephelus aeneus*	NT	NC
ACTINOPTERYGII	EPINEPHELIDAE	*Epinephelus akaara*	EN	NC
ACTINOPTERYGII	EPINEPHELIDAE	*Epinephelus albomarginatus*	VU	NC
ACTINOPTERYGII	EPINEPHELIDAE	*Epinephelus bruneus*	VU	NC
ACTINOPTERYGII	EPINEPHELIDAE	*Epinephelus fuscoguttatus*	VU	NC
ACTINOPTERYGII	EPINEPHELIDAE	*Epinephelus marginatus*	VU	NC
ACTINOPTERYGII	EPINEPHELIDAE	*Epinephelus morio*	VU	NC
ACTINOPTERYGII	EPINEPHELIDAE	*Epinephelus polyphekadion*	VU	NC
ACTINOPTERYGII	EPINEPHELIDAE	*Epinephelus striatus*	CR	NC
ACTINOPTERYGII	EPINEPHELIDAE	*Hyporthodus ergastularius*	NT	NC
ACTINOPTERYGII	EPINEPHELIDAE	*Hyporthodus niveatus*	VU	NC
ACTINOPTERYGII	EPINEPHELIDAE	*Mycteroperca bonaci*	NT	NC
ACTINOPTERYGII	EPINEPHELIDAE	*Mycteroperca interstitialis*	VU	NC
ACTINOPTERYGII	EPINEPHELIDAE	*Mycteroperca microlepis*	VU	NC
ACTINOPTERYGII	EPINEPHELIDAE	*Mycteroperca olfax*	VU	NC
ACTINOPTERYGII	EPINEPHELIDAE	*Mycteroperca venenosa*	NT	NC
ACTINOPTERYGII	EPINEPHELIDAE	*Plectropomus areolatus*	VU	NC
ACTINOPTERYGII	GOBIIDAE	*Mugilogobius hitam*	NT	NC
ACTINOPTERYGII	GOBIIDAE	*Mugilogobius sarasinorum*	EN	NC

Class	Family	Species	Red List category	CITES Appendix
ACTINOPTERYGII	HAEMULIDAE	*Brachydeuterus auritus*	NT	NC
ACTINOPTERYGII	ISTIOPHORIDAE	*Kajikia audax*	NT	NC
ACTINOPTERYGII	ISTIOPHORIDAE	*Makaira nigricans*	VU	NC
ACTINOPTERYGII	LABRIDAE	*Cheilinus undulatus*	EN	II
ACTINOPTERYGII	LABRIDAE	*Lachnolaimus maximus*	VU	NC
ACTINOPTERYGII	LATIDAE	*Lates japonicus*	VU	NC
ACTINOPTERYGII	LUTJANIDAE	*Lutjanus analis*	NT	NC
ACTINOPTERYGII	LUTJANIDAE	*Lutjanus campechanus*	VU	NC
ACTINOPTERYGII	LUTJANIDAE	*Lutjanus cyanopterus*	VU	NC
ACTINOPTERYGII	LUTJANIDAE	*Lutjanus synagris*	NT	NC
ACTINOPTERYGII	LUTJANIDAE	*Rhomboplites aurorubens*	VU	NC
ACTINOPTERYGII	MALACANTHIDAE	*Lopholatilus chamaeleonticeps*	EN	NC
ACTINOPTERYGII	MULLIDAE	*Pseudupeneus prayensis*	VU	NC
ACTINOPTERYGII	NEMIPTERIDAE	*Nemipterus virgatus*	VU	NC
ACTINOPTERYGII	OSPHRONEMIDAE	*Belontia signata*	VU	NC
ACTINOPTERYGII	OSPHRONEMIDAE	*Betta albimarginata*	EN	NC
ACTINOPTERYGII	OSPHRONEMIDAE	*Betta channoides*	EN	NC
ACTINOPTERYGII	OSPHRONEMIDAE	*Betta coccina*	VU	NC
ACTINOPTERYGII	OSPHRONEMIDAE	*Betta dennisyongi*	VU	NC
ACTINOPTERYGII	OSPHRONEMIDAE	*Betta dimidiata*	VU	NC
ACTINOPTERYGII	OSPHRONEMIDAE	*Betta macrostoma*	VU	NC
ACTINOPTERYGII	OSPHRONEMIDAE	*Betta simorum*	VU	NC
ACTINOPTERYGII	OSPHRONEMIDAE	*Betta uberis*	VU	NC
ACTINOPTERYGII	OSPHRONEMIDAE	*Malpulutta kretseri*	EN	NC
ACTINOPTERYGII	OSPHRONEMIDAE	*Osphronemus laticlavius*	EN	NC
ACTINOPTERYGII	OSPHRONEMIDAE	*Parosphromenus ornaticauda*	CR	NC
ACTINOPTERYGII	OSPHRONEMIDAE	*Parosphromenus tweediei*	EN	NC
ACTINOPTERYGII	OSPHRONEMIDAE	*Sphaerichthys vaillanti*	EN	NC
ACTINOPTERYGII	OSPHRONEMIDAE	*Trichopodus leerii*	NT	NC
ACTINOPTERYGII	POMACANTHIDAE	*Holacanthus limbaughi*	NT	NC
ACTINOPTERYGII	POMATOMIDAE	*Pomatomus saltatrix*	VU	NC
ACTINOPTERYGII	SCIAENIDAE	*Argyrosomus inodorus*	VU	NC
ACTINOPTERYGII	SCIAENIDAE	*Argyrosomus japonicus*	EN	NC
ACTINOPTERYGII	SCIAENIDAE	*Atractoscion aequidens*	NT	NC
ACTINOPTERYGII	SCIAENIDAE	*Boesemania microlepis*	NT	NC
ACTINOPTERYGII	SCIAENIDAE	*Larimichthys crocea*	CR	NC
ACTINOPTERYGII	SCIAENIDAE	*Pentheroscion mbizi*	NT	NC
ACTINOPTERYGII	SCIAENIDAE	*Protonibea diacanthus*	NT	NC
ACTINOPTERYGII	SCIAENIDAE	*Pseudotolithus senegalensis*	EN	NC
ACTINOPTERYGII	SCIAENIDAE	*Pseudotolithus senegallus*	VU	NC
ACTINOPTERYGII	SCIAENIDAE	*Totoaba macdonaldi*	CR	I
ACTINOPTERYGII	SCOMBRIDAE	*Scomberomorus commerson*	NT	NC
ACTINOPTERYGII	SCOMBRIDAE	*Scomberomorus munroi*	NT	NC
ACTINOPTERYGII	SCOMBRIDAE	*Thunnus alalunga*	NT	NC

Class	Family	Species	Red List category	CITES Appendix
ACTINOPTERYGII	SCOMBRIDAE	*Thunnus albacares*	NT	NC
ACTINOPTERYGII	SCOMBRIDAE	*Thunnus maccoyii*	CR	NC
ACTINOPTERYGII	SCOMBRIDAE	*Thunnus obesus*	VU	NC
ACTINOPTERYGII	SCOMBRIDAE	*Thunnus orientalis*	VU	NC
ACTINOPTERYGII	SCOMBRIDAE	*Thunnus thynnus*	EN	NC
ACTINOPTERYGII	SERRANIDAE	*Hypoplectrus liberte*	CR	NC
ACTINOPTERYGII	SIGANIDAE	*Siganus niger*	VU	NC
ACTINOPTERYGII	SIGANIDAE	*Siganus uspi*	NT	NC
ACTINOPTERYGII	SPARIDAE	*Chrysoblephus anglicus*	NT	NC
ACTINOPTERYGII	SPARIDAE	*Dentex dentex*	VU	NC
ACTINOPTERYGII	SPARIDAE	*Pagellus bogaraveo*	NT	NC
ACTINOPTERYGII	SALMONIDAE	*Coregonus maraena*	VU	NC
ACTINOPTERYGII	SALMONIDAE	*Hucho taimen*	VU	NC
ACTINOPTERYGII	ARIIDAE	*Sciades parkeri*	VU	NC
ACTINOPTERYGII	CALLICHTHYIDAE	*Corydoras panda*	NT	NC
ACTINOPTERYGII	CLARIIDAE	*Clarias magur*	EN	NC
ACTINOPTERYGII	ICTALURIDAE	*Prietella phreatophila*	VU	NC
ACTINOPTERYGII	LORICARIIDAE	*Otocinclus cocama*	EN	NC
ACTINOPTERYGII	LORICARIIDAE	*Panaqolus albivermis*	EN	NC
ACTINOPTERYGII	LORICARIIDAE	*Panaque cochliodon*	NT	NC
ACTINOPTERYGII	PANGASIIDAE	*Pangasianodon hypophthalmus*	EN	NC
ACTINOPTERYGII	PANGASIIDAE	*Pangasius krempfi*	VU	NC
ACTINOPTERYGII	PANGASIIDAE	*Pangasius sanitwongsei*	CR	NC
ACTINOPTERYGII	SILURIDAE	*Kryptopterus minor*	NT	NC
ACTINOPTERYGII	SILURIDAE	*Wallago attu*	VU	NC
ACTINOPTERYGII	SISORIDAE	*Bagarius bagarius*	NT	NC
ACTINOPTERYGII	SISORIDAE	*Bagarius yarrelli*	NT	NC
ACTINOPTERYGII	SISORIDAE	*Glyptothorax housei*	EN	NC
ACTINOPTERYGII	SYNGNATHIDAE	*Hippocampus algiricus*	VU	II
ACTINOPTERYGII	SYNGNATHIDAE	*Hippocampus barbouri*	VU	II
ACTINOPTERYGII	SYNGNATHIDAE	*Hippocampus comes*	VU	II
ACTINOPTERYGII	SYNGNATHIDAE	*Hippocampus erectus*	VU	II
ACTINOPTERYGII	SYNGNATHIDAE	*Hippocampus histrix*	VU	II
ACTINOPTERYGII	SYNGNATHIDAE	*Hippocampus ingens*	VU	II
ACTINOPTERYGII	SYNGNATHIDAE	*Hippocampus kelloggi*	VU	II
ACTINOPTERYGII	SYNGNATHIDAE	*Hippocampus kuda*	VU	II
ACTINOPTERYGII	SYNGNATHIDAE	*Hippocampus patagonicus*	VU	II
ACTINOPTERYGII	SYNGNATHIDAE	*Hippocampus reidi*	NT	II
ACTINOPTERYGII	SYNGNATHIDAE	*Hippocampus spinosissimus*	VU	II
ACTINOPTERYGII	SYNGNATHIDAE	*Hippocampus trimaculatus*	VU	II
ACTINOPTERYGII	SYNGNATHIDAE	*Microphis deocata*	NT	NC
ACTINOPTERYGII	BALISTIDAE	*Balistes punctatus*	VU	NC
ACTINOPTERYGII	MONACANTHIDAE	*Oxymonacanthus longirostris*	VU	NC
ACTINOPTERYGII	TETRAODONTIDAE	*Carinotetraodon travancoricus*	VU	NC

Class	Family	Species	Red List category	CITES Appendix
ACTINOPTERYGII	TETRAODONTIDAE	*Takifugu chinensis*	CR	NC
ACTINOPTERYGII	TETRAODONTIDAE	*Takifugu flavidus*	NT	NC
ACTINOPTERYGII	TETRAODONTIDAE	*Takifugu ocellatus*	NT	NC
ACTINOPTERYGII	TETRAODONTIDAE	*Takifugu rubripes*	NT	NC
ACTINOPTERYGII	TETRAODONTIDAE	*Takifugu vermicularis*	NT	NC
AMPHIBIA	BUFONIDAE	*Atelopus carbonerensis*	CR	NC
AMPHIBIA	BUFONIDAE	*Atelopus flavescens*	VU	NC
AMPHIBIA	BUFONIDAE	*Atelopus spurrelli*	NT	NC
AMPHIBIA	BUFONIDAE	*Atelopus varius*	CR	NC
AMPHIBIA	BUFONIDAE	*Atelopus zeteki*	CR	I
AMPHIBIA	BUFONIDAE	*Leptophryne cruentata*	CR	NC
AMPHIBIA	BUFONIDAE	*Rhaebo blombergi*	NT	NC
AMPHIBIA	CALYPTOCEPHALELLIDAE	*Calyptocephalella gayi*	VU	III
AMPHIBIA	CERATOPHRYIDAE	*Ceratophrys ornata*	NT	NC
AMPHIBIA	CERATOPHRYIDAE	*Ceratophrys stolzmanni*	VU	NC
AMPHIBIA	CONRAUIDAE	*Conraua goliath*	EN	NC
AMPHIBIA	DENDROBATIDAE	*Ameerega cainarachi*	EN	II
AMPHIBIA	DENDROBATIDAE	*Ameerega pongoensis*	VU	II
AMPHIBIA	DENDROBATIDAE	*Ameerega rubriventris*	EN	II
AMPHIBIA	DENDROBATIDAE	*Ameerega silverstonei*	EN	II
AMPHIBIA	DENDROBATIDAE	*Andinobates bombetes*	VU	II
AMPHIBIA	DENDROBATIDAE	*Andinobates dorisswansonae*	VU	II
AMPHIBIA	DENDROBATIDAE	*Andinobates opisthomelas*	VU	II
AMPHIBIA	DENDROBATIDAE	*Andinobates victimatus*	EN	II
AMPHIBIA	DENDROBATIDAE	*Epipedobates anthonyi*	NT	II
AMPHIBIA	DENDROBATIDAE	*Epipedobates tricolor*	VU	II
AMPHIBIA	DENDROBATIDAE	*Excidobates mysteriosus*	EN	II
AMPHIBIA	DENDROBATIDAE	*Minyobates steyermarki*	CR	II
AMPHIBIA	DENDROBATIDAE	*Oophaga anchicayensis*	EN	II
AMPHIBIA	DENDROBATIDAE	*Oophaga andresi*	EN	II
AMPHIBIA	DENDROBATIDAE	*Oophaga granulifera*	VU	II
AMPHIBIA	DENDROBATIDAE	*Oophaga histrionica*	CR	II
AMPHIBIA	DENDROBATIDAE	*Oophaga lehmanni*	CR	II
AMPHIBIA	DENDROBATIDAE	*Oophaga solanensis*	VU	II
AMPHIBIA	DENDROBATIDAE	*Oophaga speciosa*	EN	II
AMPHIBIA	DENDROBATIDAE	*Oophaga sylvatica*	NT	II
AMPHIBIA	DENDROBATIDAE	*Ranitomeya benedicta*	VU	II
AMPHIBIA	DENDROBATIDAE	*Ranitomeya fantastica*	VU	II
AMPHIBIA	DENDROBATIDAE	*Ranitomeya summersi*	EN	II
AMPHIBIA	DICROGLOSSIDAE	*Limnonectes blythii*	NT	NC
AMPHIBIA	DICROGLOSSIDAE	*Limnonectes magnus*	NT	NC
AMPHIBIA	DICROGLOSSIDAE	*Limnonectes microtympanum*	EN	NC
AMPHIBIA	HYLIDAE	*Osteopilus vastus*	VU	NC
AMPHIBIA	HYLIDAE	*Phytotriades auratus*	CR	NC
AMPHIBIA	HYPEROLIIDAE	*Kassina arboricola*	VU	NC

**Class**	**Family**	**Species**	**Red List category**	**CITES Appendix**
AMPHIBIA	LEPTODACTYLIDAE	*Leptodactylus fallax*	CR	NC
AMPHIBIA	LEPTODACTYLIDAE	*Leptodactylus laticeps*	NT	NC
AMPHIBIA	MANTELLIDAE	*Mantella cowanii*	EN	II
AMPHIBIA	MANTELLIDAE	*Mantella crocea*	VU	II
AMPHIBIA	MANTELLIDAE	*Mantella viridis*	EN	II
AMPHIBIA	MICROHYLIDAE	*Scaphiophryne gottlebei*	EN	II
AMPHIBIA	PHYLLOMEDUSIDAE	*Agalychnis annae*	EN	NC
AMPHIBIA	RANIDAE	*Amolops loloensis*	VU	NC
AMPHIBIA	RANIDAE	*Odorrana ishikawae*	EN	NC
AMPHIBIA	RANIDAE	*Odorrana splendida*	EN	NC
AMPHIBIA	RANIDAE	*Pelophylax caralitanus*	NT	NC
AMPHIBIA	RANIDAE	*Pelophylax epeiroticus*	VU	NC
AMPHIBIA	RANIDAE	*Pelophylax shqipericus*	EN	NC
AMPHIBIA	RHACOPHORIDAE	*Gracixalus lumarius*	EN	NC
AMPHIBIA	RHACOPHORIDAE	*Rhacophorus calcaneus*	EN	NC
AMPHIBIA	RHACOPHORIDAE	*Rhacophorus helenae*	EN	NC
AMPHIBIA	RHACOPHORIDAE	*Theloderma palliatum*	EN	NC
AMPHIBIA	HYNOBIIDAE	*Hynobius boulengeri*	EN	NC
AMPHIBIA	HYNOBIIDAE	*Hynobius tokyoensis*	VU	NC
AMPHIBIA	HYNOBIIDAE	*Pachyhynobius shangchengensis*	VU	NC
AMPHIBIA	PLETHODONTIDAE	*Plethodon petraeus*	VU	NC
AMPHIBIA	PLETHODONTIDAE	*Speleomantes ambrosii*	NT	NC
AMPHIBIA	PLETHODONTIDAE	*Speleomantes flavus*	VU	NC
AMPHIBIA	PLETHODONTIDAE	*Speleomantes genei*	VU	NC
AMPHIBIA	PLETHODONTIDAE	*Speleomantes imperialis*	NT	NC
AMPHIBIA	PLETHODONTIDAE	*Speleomantes italicus*	NT	NC
AMPHIBIA	PLETHODONTIDAE	*Speleomantes sarrabusensis*	VU	NC
AMPHIBIA	PLETHODONTIDAE	*Speleomantes strinatii*	NT	NC
AMPHIBIA	PLETHODONTIDAE	*Speleomantes supramontis*	EN	NC
AMPHIBIA	PROTEIDAE	*Proteus anguinus*	VU	NC
AMPHIBIA	SALAMANDRIDAE	*Cynops ensicauda*	EN	NC
AMPHIBIA	SALAMANDRIDAE	*Echinotriton andersoni*	EN	III
AMPHIBIA	SALAMANDRIDAE	*Laotriton laoensis*	EN	NC
AMPHIBIA	SALAMANDRIDAE	*Liangshantriton taliangensis*	NT	NC
AMPHIBIA	SALAMANDRIDAE	*Lyciasalamandra antalyana*	EN	NC
AMPHIBIA	SALAMANDRIDAE	*Lyciasalamandra atifi*	EN	NC
AMPHIBIA	SALAMANDRIDAE	*Lyciasalamandra fazilae*	EN	NC
AMPHIBIA	SALAMANDRIDAE	*Lyciasalamandra flavimembris*	EN	NC
AMPHIBIA	SALAMANDRIDAE	*Mertensiella caucasica*	VU	NC
AMPHIBIA	SALAMANDRIDAE	*Neurergus kaiseri*	VU	I
AMPHIBIA	SALAMANDRIDAE	*Neurergus microspilotus*	CR	NC
AMPHIBIA	SALAMANDRIDAE	*Notophthalmus perstriatus*	NT	NC
AMPHIBIA	SALAMANDRIDAE	*Ommatotriton ophryticus*	NT	NC
AMPHIBIA	SALAMANDRIDAE	*Pachytriton archospotus*	NT	NC

Class	Family	Species	Red List category	CITES Appendix
AMPHIBIA	SALAMANDRIDAE	*Pachytriton feii*	VU	NC
AMPHIBIA	SALAMANDRIDAE	*Pachytriton wuguanfui*	EN	NC
AMPHIBIA	SALAMANDRIDAE	*Paramesotriton aurantius*	VU	II
AMPHIBIA	SALAMANDRIDAE	*Paramesotriton caudopunctatus*	NT	II
AMPHIBIA	SALAMANDRIDAE	*Paramesotriton fuzhongensis*	VU	II
AMPHIBIA	SALAMANDRIDAE	*Paramesotriton hongkongensis*	NT	II
AMPHIBIA	SALAMANDRIDAE	*Salamandra algira*	VU	III
AMPHIBIA	SALAMANDRIDAE	*Tylototriton kweichowensis*	VU	II
AMPHIBIA	SALAMANDRIDAE	*Tylototriton notialis*	VU	II
AMPHIBIA	SALAMANDRIDAE	*Tylototriton shanjing*	NT	II
AMPHIBIA	SALAMANDRIDAE	*Tylototriton shanorum*	VU	II
AMPHIBIA	SALAMANDRIDAE	*Tylototriton vietnamensis*	EN	II
AMPHIBIA	SALAMANDRIDAE	*Tylototriton wenxianensis*	VU	II
ANTHOZOA	TUBIPORIDAE	*Tubipora musica*	NT	II
ANTHOZOA	HELIOPORIDAE	*Heliopora coerulea*	VU	II
ANTHOZOA	ACROPORIDAE	*Acropora abrolhosensis*	VU	II
ANTHOZOA	ACROPORIDAE	*Acropora aculeus*	VU	II
ANTHOZOA	ACROPORIDAE	*Acropora acuminata*	VU	II
ANTHOZOA	ACROPORIDAE	*Acropora aspera*	VU	II
ANTHOZOA	ACROPORIDAE	*Acropora austera*	NT	II
ANTHOZOA	ACROPORIDAE	*Acropora carduus*	NT	II
ANTHOZOA	ACROPORIDAE	*Acropora caroliniana*	VU	II
ANTHOZOA	ACROPORIDAE	*Acropora desalwii*	VU	II
ANTHOZOA	ACROPORIDAE	*Acropora digitifera*	NT	II
ANTHOZOA	ACROPORIDAE	*Acropora divaricata*	NT	II
ANTHOZOA	ACROPORIDAE	*Acropora echinata*	VU	II
ANTHOZOA	ACROPORIDAE	*Acropora elegans*	VU	II
ANTHOZOA	ACROPORIDAE	*Acropora florida*	NT	II
ANTHOZOA	ACROPORIDAE	*Acropora formosa*	NT	II
ANTHOZOA	ACROPORIDAE	*Acropora granulosa*	NT	II
ANTHOZOA	ACROPORIDAE	*Acropora horrida*	VU	II
ANTHOZOA	ACROPORIDAE	*Acropora humilis*	NT	II
ANTHOZOA	ACROPORIDAE	*Acropora jacquelineae*	VU	II
ANTHOZOA	ACROPORIDAE	*Acropora kimbeensis*	VU	II
ANTHOZOA	ACROPORIDAE	*Acropora loisetteae*	VU	II
ANTHOZOA	ACROPORIDAE	*Acropora lokani*	VU	II
ANTHOZOA	ACROPORIDAE	*Acropora loripes*	NT	II
ANTHOZOA	ACROPORIDAE	*Acropora lutkeni*	NT	II
ANTHOZOA	ACROPORIDAE	*Acropora microclados*	VU	II
ANTHOZOA	ACROPORIDAE	*Acropora millepora*	NT	II
ANTHOZOA	ACROPORIDAE	*Acropora multiacuta*	VU	II
ANTHOZOA	ACROPORIDAE	*Acropora nana*	NT	II
ANTHOZOA	ACROPORIDAE	*Acropora nasuta*	NT	II
ANTHOZOA	ACROPORIDAE	*Acropora paniculata*	VU	II

Class	Family	Species	Red List category	CITES Appendix
ANTHOZOA	ACROPORIDAE	*Acropora pichoni*	NT	II
ANTHOZOA	ACROPORIDAE	*Acropora polystoma*	VU	II
ANTHOZOA	ACROPORIDAE	*Acropora secale*	NT	II
ANTHOZOA	ACROPORIDAE	*Acropora selago*	NT	II
ANTHOZOA	ACROPORIDAE	*Acropora simplex*	VU	II
ANTHOZOA	ACROPORIDAE	*Acropora suharsonoi*	EN	II
ANTHOZOA	ACROPORIDAE	*Acropora tenella*	VU	II
ANTHOZOA	ACROPORIDAE	*Acropora tenuis*	NT	II
ANTHOZOA	ACROPORIDAE	*Acropora turaki*	VU	II
ANTHOZOA	ACROPORIDAE	*Acropora vaughani*	VU	II
ANTHOZOA	ACROPORIDAE	*Acropora verweyi*	VU	II
ANTHOZOA	ACROPORIDAE	*Acropora willisae*	VU	II
ANTHOZOA	ACROPORIDAE	*Montipora altasepta*	VU	II
ANTHOZOA	ACROPORIDAE	*Montipora angulata*	VU	II
ANTHOZOA	ACROPORIDAE	*Montipora australiensis*	VU	II
ANTHOZOA	ACROPORIDAE	*Montipora cactus*	VU	II
ANTHOZOA	ACROPORIDAE	*Montipora calcarea*	VU	II
ANTHOZOA	ACROPORIDAE	*Montipora caliculata*	VU	II
ANTHOZOA	ACROPORIDAE	*Montipora capitata*	NT	II
ANTHOZOA	ACROPORIDAE	*Montipora capricornis*	VU	II
ANTHOZOA	ACROPORIDAE	*Montipora cebuensis*	VU	II
ANTHOZOA	ACROPORIDAE	*Montipora cocosensis*	VU	II
ANTHOZOA	ACROPORIDAE	*Montipora confusa*	NT	II
ANTHOZOA	ACROPORIDAE	*Montipora corbettensis*	VU	II
ANTHOZOA	ACROPORIDAE	*Montipora crassituberculata*	VU	II
ANTHOZOA	ACROPORIDAE	*Montipora delicatula*	VU	II
ANTHOZOA	ACROPORIDAE	*Montipora florida*	VU	II
ANTHOZOA	ACROPORIDAE	*Montipora foliosa*	NT	II
ANTHOZOA	ACROPORIDAE	*Montipora foveolata*	NT	II
ANTHOZOA	ACROPORIDAE	*Montipora friabilis*	VU	II
ANTHOZOA	ACROPORIDAE	*Montipora gaimardi*	VU	II
ANTHOZOA	ACROPORIDAE	*Montipora hodgsoni*	VU	II
ANTHOZOA	ACROPORIDAE	*Montipora mactanensis*	VU	II
ANTHOZOA	ACROPORIDAE	*Montipora nodosa*	NT	II
ANTHOZOA	ACROPORIDAE	*Montipora palawanensis*	NT	II
ANTHOZOA	ACROPORIDAE	*Montipora peltiformis*	NT	II
ANTHOZOA	ACROPORIDAE	*Montipora porites*	NT	II
ANTHOZOA	ACROPORIDAE	*Montipora samarensis*	VU	II
ANTHOZOA	ACROPORIDAE	*Montipora saudii*	NT	II
ANTHOZOA	ACROPORIDAE	*Montipora setosa*	EN	II
ANTHOZOA	ACROPORIDAE	*Montipora undata*	NT	II
ANTHOZOA	ACROPORIDAE	*Montipora vietnamensis*	VU	II
ANTHOZOA	AGARICIIDAE	*Pachyseris rugosa*	VU	II
ANTHOZOA	AGARICIIDAE	*Pavona cactus*	VU	II
ANTHOZOA	AGARICIIDAE	*Pavona decussata*	VU	II

**Class**	**Family**	**Species**	**Red List category**	**CITES Appendix**
ANTHOZOA	DENDROPHYLLIIDAE	*Turbinaria mesenterina*	VU	II
ANTHOZOA	DENDROPHYLLIIDAE	*Turbinaria patula*	VU	II
ANTHOZOA	DENDROPHYLLIIDAE	*Turbinaria peltata*	VU	II
ANTHOZOA	DENDROPHYLLIIDAE	*Turbinaria reniformis*	VU	II
ANTHOZOA	EUPHYLLIDAE	*Catalaphyllia jardinei*	VU	II
ANTHOZOA	EUPHYLLIDAE	*Euphyllia ancora*	VU	II
ANTHOZOA	EUPHYLLIDAE	*Euphyllia cristata*	VU	II
ANTHOZOA	EUPHYLLIDAE	*Euphyllia divisa*	NT	II
ANTHOZOA	EUPHYLLIDAE	*Euphyllia glabrescens*	NT	II
ANTHOZOA	EUPHYLLIDAE	*Euphyllia paraancora*	VU	II
ANTHOZOA	EUPHYLLIDAE	*Euphyllia paradivisa*	VU	II
ANTHOZOA	EUPHYLLIDAE	*Euphyllia yaeyamaensis*	NT	II
ANTHOZOA	EUPHYLLIDAE	*Nemenzophyllia turbida*	VU	II
ANTHOZOA	EUPHYLLIDAE	*Physogyra lichtensteini*	VU	II
ANTHOZOA	EUPHYLLIDAE	*Plerogyra discus*	VU	II
ANTHOZOA	EUPHYLLIDAE	*Plerogyra simplex*	NT	II
ANTHOZOA	EUPHYLLIDAE	*Plerogyra sinuosa*	NT	II
ANTHOZOA	FAVIIDAE	*Australogyra zelli*	VU	II
ANTHOZOA	FAVIIDAE	*Caulastrea curvata*	VU	II
ANTHOZOA	FAVIIDAE	*Caulastrea echinulata*	VU	II
ANTHOZOA	FAVIIDAE	*Caulastrea tumida*	NT	II
ANTHOZOA	FAVIIDAE	*Diploastrea heliopora*	NT	II
ANTHOZOA	FAVIIDAE	*Favia helianthoides*	NT	II
ANTHOZOA	FAVIIDAE	*Favia lacuna*	NT	II
ANTHOZOA	FAVIIDAE	*Favia laxa*	NT	II
ANTHOZOA	FAVIIDAE	*Favia lizardensis*	NT	II
ANTHOZOA	FAVIIDAE	*Favia maritima*	NT	II
ANTHOZOA	FAVIIDAE	*Favia marshae*	NT	II
ANTHOZOA	FAVIIDAE	*Favia matthaii*	NT	II
ANTHOZOA	FAVIIDAE	*Favia maxima*	NT	II
ANTHOZOA	FAVIIDAE	*Favia rosaria*	VU	II
ANTHOZOA	FAVIIDAE	*Favia rotundata*	NT	II
ANTHOZOA	FAVIIDAE	*Favia stelligera*	NT	II
ANTHOZOA	FAVIIDAE	*Favia veroni*	NT	II
ANTHOZOA	FAVIIDAE	*Favia vietnamensis*	NT	II
ANTHOZOA	FAVIIDAE	*Favites abdita*	NT	II
ANTHOZOA	FAVIIDAE	*Favites acuticollis*	NT	II
ANTHOZOA	FAVIIDAE	*Favites bestae*	NT	II
ANTHOZOA	FAVIIDAE	*Favites chinensis*	NT	II
ANTHOZOA	FAVIIDAE	*Favites complanata*	NT	II
ANTHOZOA	FAVIIDAE	*Favites flexuosa*	NT	II
ANTHOZOA	FAVIIDAE	*Favites halicora*	NT	II
ANTHOZOA	FAVIIDAE	*Favites micropentagona*	NT	II
ANTHOZOA	FAVIIDAE	*Favites paraflexuosa*	NT	II
ANTHOZOA	FAVIIDAE	*Favites russelli*	NT	II

Class	Family	Species	Red List category	CITES Appendix
ANTHOZOA	FAVIIDAE	*Favites spinosa*	VU	II
ANTHOZOA	FAVIIDAE	*Favites stylifera*	NT	II
ANTHOZOA	FAVIIDAE	*Favites vasta*	NT	II
ANTHOZOA	FAVIIDAE	*Goniastrea columella*	NT	II
ANTHOZOA	FAVIIDAE	*Goniastrea deformis*	VU	II
ANTHOZOA	FAVIIDAE	*Goniastrea favulus*	NT	II
ANTHOZOA	FAVIIDAE	*Goniastrea minuta*	NT	II
ANTHOZOA	FAVIIDAE	*Goniastrea palauensis*	NT	II
ANTHOZOA	FAVIIDAE	*Goniastrea ramosa*	VU	II
ANTHOZOA	FAVIIDAE	*Leptoria phrygia*	NT	II
ANTHOZOA	FAVIIDAE	*Montastrea annuligera*	NT	II
ANTHOZOA	FAVIIDAE	*Montastrea colemani*	NT	II
ANTHOZOA	FAVIIDAE	*Montastrea magnistellata*	NT	II
ANTHOZOA	FAVIIDAE	*Montastrea valenciennesi*	NT	II
ANTHOZOA	FUNGIIDAE	*Fungia fungites*	NT	II
ANTHOZOA	FUNGIIDAE	*Heliofungia actiniformis*	VU	II
ANTHOZOA	MERULINIDAE	*Hydnophora exesa*	NT	II
ANTHOZOA	MERULINIDAE	*Hydnophora microconos*	NT	II
ANTHOZOA	MUSSIDAE	*Acanthastrea lordhowensis*	NT	II
ANTHOZOA	MUSSIDAE	*Blastomussa wellsi*	NT	II
ANTHOZOA	MUSSIDAE	*Cynarina lacrymalis*	NT	II
ANTHOZOA	MUSSIDAE	*Lobophyllia diminuta*	VU	II
ANTHOZOA	MUSSIDAE	*Lobophyllia serratus*	EN	II
ANTHOZOA	MUSSIDAE	*Scolymia vitiensis*	NT	II
ANTHOZOA	OCULINIDAE	*Galaxea astreata*	VU	II
ANTHOZOA	OCULINIDAE	*Galaxea fascicularis*	NT	II
ANTHOZOA	PECTINIIDAE	*Pectinia lactuca*	VU	II
ANTHOZOA	PECTINIIDAE	*Pectinia paeonia*	NT	II
ANTHOZOA	POCILLOPORIDAE	*Pocillopora danae*	VU	II
ANTHOZOA	POCILLOPORIDAE	*Pocillopora eydouxi*	NT	II
ANTHOZOA	POCILLOPORIDAE	*Seriatopora caliendrum*	NT	II
ANTHOZOA	POCILLOPORIDAE	*Stylophora pistillata*	NT	II
ANTHOZOA	PORITIDAE	*Alveopora allingi*	VU	II
ANTHOZOA	PORITIDAE	*Alveopora catalai*	NT	II
ANTHOZOA	PORITIDAE	*Alveopora daedalea*	VU	II
ANTHOZOA	PORITIDAE	*Alveopora excelsa*	EN	II
ANTHOZOA	PORITIDAE	*Alveopora fenestrata*	VU	II
ANTHOZOA	PORITIDAE	*Alveopora gigas*	VU	II
ANTHOZOA	PORITIDAE	*Alveopora japonica*	VU	II
ANTHOZOA	PORITIDAE	*Alveopora marionensis*	VU	II
ANTHOZOA	PORITIDAE	*Alveopora minuta*	EN	II
ANTHOZOA	PORITIDAE	*Alveopora spongiosa*	NT	II
ANTHOZOA	PORITIDAE	*Alveopora verrilliana*	VU	II
ANTHOZOA	PORITIDAE	*Alveopora viridis*	NT	II
ANTHOZOA	PORITIDAE	*Goniopora albiconus*	VU	II

Class	Family	Species	Red List category	CITES Appendix
ANTHOZOA	PORITIDAE	*Goniopora burgosi*	VU	II
ANTHOZOA	PORITIDAE	*Goniopora cellulosa*	VU	II
ANTHOZOA	PORITIDAE	*Goniopora columna*	NT	II
ANTHOZOA	PORITIDAE	*Goniopora lobata*	NT	II
ANTHOZOA	PORITIDAE	*Goniopora minor*	NT	II
ANTHOZOA	PORITIDAE	*Goniopora stokesi*	NT	II
ANTHOZOA	PORITIDAE	*Porites annae*	NT	II
ANTHOZOA	PORITIDAE	*Porites aranetai*	VU	II
ANTHOZOA	PORITIDAE	*Porites attenuata*	VU	II
ANTHOZOA	PORITIDAE	*Porites cocosensis*	VU	II
ANTHOZOA	PORITIDAE	*Porites cumulatus*	VU	II
ANTHOZOA	PORITIDAE	*Porites cylindrica*	NT	II
ANTHOZOA	PORITIDAE	*Porites deformis*	NT	II
ANTHOZOA	PORITIDAE	*Porites densa*	NT	II
ANTHOZOA	PORITIDAE	*Porites desilveri*	EN	II
ANTHOZOA	PORITIDAE	*Porites echinulata*	NT	II
ANTHOZOA	PORITIDAE	*Porites eridani*	EN	II
ANTHOZOA	PORITIDAE	*Porites harrisoni*	NT	II
ANTHOZOA	PORITIDAE	*Porites horizontalata*	VU	II
ANTHOZOA	PORITIDAE	*Porites lobata*	NT	II
ANTHOZOA	PORITIDAE	*Porites murrayensis*	NT	II
ANTHOZOA	PORITIDAE	*Porites napopora*	VU	II
ANTHOZOA	PORITIDAE	*Porites negrosensis*	NT	II
ANTHOZOA	PORITIDAE	*Porites nigrescens*	VU	II
ANTHOZOA	PORITIDAE	*Porites okinawensis*	VU	II
ANTHOZOA	PORITIDAE	*Porites ornata*	EN	II
ANTHOZOA	PORITIDAE	*Porites rugosa*	VU	II
ANTHOZOA	PORITIDAE	*Porites sillimaniana*	VU	II
ANTHOZOA	PORITIDAE	*Porites somaliensis*	NT	II
ANTHOZOA	PORITIDAE	*Porites stephensoni*	NT	II
ANTHOZOA	PORITIDAE	*Porites tuberculosa*	VU	II
ANTHOZOA	SIDERASTREIDAE	*Psammocora contigua*	NT	II
ANTHOZOA	SIDERASTREIDAE	*Psammocora stellata*	VU	II
ANTHOZOA	TRACHYPHYLLIIDAE	*Trachyphyllia geoffroyi*	NT	II
ARACHNIDA	THERAPHOSIDAE	*Brachypelma auratum*	NT	II
ARACHNIDA	THERAPHOSIDAE	*Brachypelma baumgarteni*	EN	II
ARACHNIDA	THERAPHOSIDAE	*Brachypelma boehmei*	EN	II
ARACHNIDA	THERAPHOSIDAE	*Brachypelma hamorii*	VU	II
ARACHNIDA	THERAPHOSIDAE	*Brachypelma klaasi*	NT	II
ARACHNIDA	THERAPHOSIDAE	*Brachypelma schroederi*	EN	II
ARACHNIDA	THERAPHOSIDAE	*Brachypelma smithi*	NT	II
ARACHNIDA	THERAPHOSIDAE	*Brachypelma verdezi*	NT	II
ARACHNIDA	THERAPHOSIDAE	*Grammostola vachoni*	VU	NC
ARACHNIDA	THERAPHOSIDAE	*Haploclastus kayi*	EN	NC
ARACHNIDA	THERAPHOSIDAE	*Poecilotheria formosa*	EN	II

**Class**	**Family**	**Species**	**Red List category**	**CITES Appendix**
ARACHNIDA	THERAPHOSIDAE	*Poecilotheria hanumavilasumica*	CR	II
ARACHNIDA	THERAPHOSIDAE	*Poecilotheria metallica*	CR	II
ARACHNIDA	THERAPHOSIDAE	*Poecilotheria miranda*	EN	II
ARACHNIDA	THERAPHOSIDAE	*Poecilotheria rufilata*	EN	II
ARACHNIDA	THERAPHOSIDAE	*Poecilotheria striata*	VU	II
ARACHNIDA	THERAPHOSIDAE	*Thrigmopoeus insignis*	VU	NC
ARACHNIDA	THERAPHOSIDAE	*Thrigmopoeus truculentus*	NT	NC
AVES	ACCIPITRIDAE	*Accipiter gundlachi*	EN	II
AVES	ACCIPITRIDAE	*Aquila heliaca*	VU	I
AVES	ACCIPITRIDAE	*Aquila nipalensis*	EN	II
AVES	ACCIPITRIDAE	*Buteogallus coronatus*	EN	II
AVES	ACCIPITRIDAE	*Circaetus beaudouini*	VU	II
AVES	ACCIPITRIDAE	*Gyps africanus*	CR	II
AVES	ACCIPITRIDAE	*Gyps rueppelli*	CR	II
AVES	ACCIPITRIDAE	*Haliaeetus sanfordi*	VU	II
AVES	ACCIPITRIDAE	*Haliaeetus vociferoides*	CR	II
AVES	ACCIPITRIDAE	*Spizaetus ornatus*	NT	II
AVES	ACCIPITRIDAE	*Terathopius ecaudatus*	NT	II
AVES	ACCIPITRIDAE	*Trigonoceps occipitalis*	CR	II
AVES	SAGITTARIIDAE	*Sagittarius serpentarius*	VU	II
AVES	ANATIDAE	*Asarcornis scutulata*	EN	I
AVES	ANATIDAE	*Aythya baeri*	CR	NC
AVES	ANATIDAE	*Marmaronetta angustirostris*	VU	NC
AVES	ANATIDAE	*Melanitta americana*	NT	NC
AVES	ANATIDAE	*Neochen jubata*	NT	NC
AVES	ANATIDAE	*Oxyura leucocephala*	EN	II
AVES	ANATIDAE	*Oxyura maccoa*	VU	NC
AVES	BUCEROTIDAE	*Anthracoceros marchei*	VU	II
AVES	BUCEROTIDAE	*Berenicornis comatus*	EN	II
AVES	BUCEROTIDAE	*Buceros bicornis*	VU	I
AVES	BUCEROTIDAE	*Bucorvus leadbeateri*	VU	NC
AVES	BUCEROTIDAE	*Bycanistes cylindricus*	VU	NC
AVES	BUCEROTIDAE	*Rhinoplax vigil*	CR	I
AVES	BUCEROTIDAE	*Rhyticeros subruficollis*	VU	I
AVES	SCOLOPACIDAE	*Numenius arquata*	NT	NC
AVES	CICONIIDAE	*Ciconia boyciana*	EN	I
AVES	CICONIIDAE	*Ciconia stormi*	EN	NC
AVES	CICONIIDAE	*Leptoptilos javanicus*	VU	NC
AVES	COLUMBIDAE	*Gallicolumba crinigera*	VU	NC
AVES	COLUMBIDAE	*Gallicolumba luzonica*	NT	II
AVES	COLUMBIDAE	*Goura cristata*	VU	II
AVES	COLUMBIDAE	*Patagioenas caribaea*	VU	NC
AVES	COLUMBIDAE	*Patagioenas oenops*	VU	NC
AVES	COLUMBIDAE	*Ramphiculus jambu*	NT	NC

Class	Family	Species	Red List category	CITES Appendix
AVES	COLUMBIDAE	*Ramphiculus marchei*	VU	NC
AVES	CUCULIDAE	*Carpococcyx renauldi*	VU	NC
AVES	FALCONIDAE	*Falco cherrug*	EN	II
AVES	FALCONIDAE	*Falco jugger*	NT	I
AVES	FALCONIDAE	*Falco vespertinus*	NT	II
AVES	CRACIDAE	*Crax pinima*	CR	NC
AVES	CRACIDAE	*Crax rubra*	VU	III
AVES	CRACIDAE	*Mitu tomentosum*	NT	NC
AVES	CRACIDAE	*Penelope pileata*	VU	NC
AVES	CRACIDAE	*Penelopina nigra*	VU	III
AVES	MEGAPODIIDAE	*Macrocephalon maleo*	EN	I
AVES	MEGAPODIIDAE	*Megapodius pritchardii*	VU	NC
AVES	PHASIANIDAE	*Arborophila atrogularis*	NT	NC
AVES	PHASIANIDAE	*Arborophila charltonii*	VU	NC
AVES	PHASIANIDAE	*Argusianus argus*	NT	II
AVES	PHASIANIDAE	*Caloperdix oculeus*	NT	NC
AVES	PHASIANIDAE	*Lophophorus lhuysii*	VU	I
AVES	PHASIANIDAE	*Lophura edwardsi*	CR	I
AVES	PHASIANIDAE	*Lophura inornata*	NT	NC
AVES	PHASIANIDAE	*Meleagris ocellata*	NT	III
AVES	PHASIANIDAE	*Polyplectron malacense*	VU	II
AVES	PHASIANIDAE	*Polyplectron schleiermacheri*	EN	II
AVES	PHASIANIDAE	*Rheinardia ocellata*	EN	I
AVES	PHASIANIDAE	*Rollulus rouloul*	NT	NC
AVES	GRUIDAE	*Anthropoides paradiseus*	VU	II
AVES	GRUIDAE	*Balearica regulorum*	EN	II
AVES	GRUIDAE	*Bugeranus carunculatus*	VU	II
AVES	GRUIDAE	*Grus japonensis*	EN	I
AVES	GRUIDAE	*Grus monacha*	VU	I
AVES	GRUIDAE	*Grus nigricollis*	VU	I
AVES	MUSOPHAGIDAE	*Tauraco fischeri*	NT	II
AVES	OTIDIDAE	*Ardeotis kori*	NT	II
AVES	OTIDIDAE	*Ardeotis nigriceps*	CR	I
AVES	OTIDIDAE	*Chlamydotis macqueenii*	VU	I
AVES	OTIDIDAE	*Chlamydotis undulata*	VU	I
AVES	OTIDIDAE	*Neotis denhami*	NT	II
AVES	OTIDIDAE	*Neotis nuba*	NT	II
AVES	OTIDIDAE	*Otis tarda*	VU	II
AVES	BOMBYCILLIDAE	*Bombycilla japonica*	NT	NC
AVES	CHLOROPSEIDAE	*Chloropsis cochinchinensis*	EN	NC
AVES	CHLOROPSEIDAE	*Chloropsis media*	EN	NC
AVES	CHLOROPSEIDAE	*Chloropsis sonnerati*	EN	NC
AVES	CHLOROPSEIDAE	*Chloropsis venusta*	NT	NC
AVES	CORVIDAE	*Corvus pectoralis*	VU	NC
AVES	COTINGIDAE	*Cotinga maculata*	CR	I

Class	Family	Species	Red List category	CITES Appendix
AVES	COTINGIDAE	*Procnias nudicollis*	VU	NC
AVES	COTINGIDAE	*Xipholena atropurpurea*	VU	I
AVES	EMBERIZIDAE	*Emberiza aureola*	CR	NC
AVES	EMBERIZIDAE	*Emberiza rustica*	VU	NC
AVES	EMBERIZIDAE	*Emberiza sulphurata*	VU	NC
AVES	ESTRILDIDAE	*Amandava formosa*	VU	II
AVES	ESTRILDIDAE	*Lonchura fuscata*	NT	NC
AVES	ESTRILDIDAE	*Lonchura oryzivora*	EN	II
AVES	ESTRILDIDAE	*Lonchura stygia*	NT	NC
AVES	FRINGILLIDAE	*Fringilla polatzeki*	EN	NC
AVES	FRINGILLIDAE	*Fringilla teydea*	NT	NC
AVES	FRINGILLIDAE	*Spinus cucullatus*	EN	I
AVES	FRINGILLIDAE	*Spinus yarrellii*	VU	II
AVES	PARADISAEIDAE	*Paradisornis rudolphi*	VU	II
AVES	PARIDAE	*Machlolophus holsti*	NT	NC
AVES	PICATHARTIDAE	*Picathartes oreas*	VU	I
AVES	PITTIDAE	*Hydrornis gurneyi*	CR	I
AVES	PLATYLOPHIDAE	*Platylophus galericulatus*	NT	NC
AVES	PYCNONOTIDAE	*Alophoixus bres*	NT	NC
AVES	PYCNONOTIDAE	*Pycnonotus bimaculatus*	NT	NC
AVES	PYCNONOTIDAE	*Pycnonotus snouckaerti*	VU	NC
AVES	PYCNONOTIDAE	*Pycnonotus zeylanicus*	CR	II
AVES	PYCNONOTIDAE	*Rubigula dispar*	VU	NC
AVES	STURNIDAE	*Acridotheres cinereus*	VU	NC
AVES	STURNIDAE	*Acridotheres javanicus*	VU	NC
AVES	STURNIDAE	*Acridotheres melanopterus*	CR	NC
AVES	STURNIDAE	*Acridotheres tertius*	CR	NC
AVES	STURNIDAE	*Acridotheres tricolor*	CR	NC
AVES	STURNIDAE	*Gracula venerata*	EN	NC
AVES	STURNIDAE	*Leucopsar rothschildi*	CR	I
AVES	THRAUPIDAE	*Pyrrhulagra nigra*	NT	NC
AVES	THRAUPIDAE	*Tangara fastuosa*	VU	II
AVES	TURDIDAE	*Geokichla dohertyi*	NT	NC
AVES	ZOSTEROPIDAE	*Zosterops flavus*	EN	NC
AVES	ZOSTEROPIDAE	*Zosterops melanurus*	VU	NC
AVES	BALAENICIPITIDAE	*Balaeniceps rex*	VU	II
AVES	PELECANIDAE	*Pelecanus crispus*	NT	I
AVES	THRESKIORNITHIDAE	*Geronticus eremita*	EN	I
AVES	THRESKIORNITHIDAE	*Lophotibis cristata*	NT	NC
AVES	PHOENICOPTERIDAE	*Phoenicopterus chilensis*	NT	II
AVES	MEGALAIMIDAE	*Psilopogon javensis*	NT	NC
AVES	PICIDAE	*Picus sharpei*	NT	NC
AVES	RAMPHASTIDAE	*Andigena laminirostris*	NT	NC
AVES	RAMPHASTIDAE	*Pteroglossus bailloni*	NT	III
AVES	SEMNORNITHIDAE	*Semnornis ramphastinus*	NT	III

Class	Family	Species	Red List category	CITES Appendix
AVES	CACATUIDAE	*Cacatua alba*	EN	II
AVES	CACATUIDAE	*Cacatua goffiniana*	NT	I
AVES	CACATUIDAE	*Cacatua haematuropygia*	CR	I
AVES	CACATUIDAE	*Cacatua sulphurea*	CR	I
AVES	CACATUIDAE	*Zanda baudinii*	EN	II
AVES	CACATUIDAE	*Zanda latirostris*	EN	II
AVES	PSITTACIDAE	*Agapornis fischeri*	NT	II
AVES	PSITTACIDAE	*Agapornis lilianae*	NT	II
AVES	PSITTACIDAE	*Alipiopsitta xanthops*	NT	II
AVES	PSITTACIDAE	*Amazona aestiva*	NT	II
AVES	PSITTACIDAE	*Amazona arausiaca*	VU	I
AVES	PSITTACIDAE	*Amazona auropalliata*	EN	I
AVES	PSITTACIDAE	*Amazona barbadensis*	VU	I
AVES	PSITTACIDAE	*Amazona bodini*	NT	II
AVES	PSITTACIDAE	*Amazona brasiliensis*	NT	I
AVES	PSITTACIDAE	*Amazona dufresniana*	NT	II
AVES	PSITTACIDAE	*Amazona farinosa*	NT	II
AVES	PSITTACIDAE	*Amazona festiva*	NT	II
AVES	PSITTACIDAE	*Amazona finschi*	EN	I
AVES	PSITTACIDAE	*Amazona guatemalae*	NT	II
AVES	PSITTACIDAE	*Amazona imperialis*	CR	I
AVES	PSITTACIDAE	*Amazona versicolor*	VU	I
AVES	PSITTACIDAE	*Amazona viridigenalis*	EN	I
AVES	PSITTACIDAE	*Anodorhynchus hyacinthinus*	VU	I
AVES	PSITTACIDAE	*Anodorhynchus leari*	EN	I
AVES	PSITTACIDAE	*Ara glaucogularis*	CR	I
AVES	PSITTACIDAE	*Ara rubrogenys*	CR	I
AVES	PSITTACIDAE	*Aratinga solstitialis*	EN	II
AVES	PSITTACIDAE	*Brotogeris pyrrhoptera*	EN	II
AVES	PSITTACIDAE	*Eclectus cornelia*	EN	II
AVES	PSITTACIDAE	*Eos cyanogenia*	VU	II
AVES	PSITTACIDAE	*Eos histrio*	EN	I
AVES	PSITTACIDAE	*Eos semilarvata*	NT	II
AVES	PSITTACIDAE	*Forpus cyanopygius*	NT	II
AVES	PSITTACIDAE	*Lathamus discolor*	CR	II
AVES	PSITTACIDAE	*Lorius garrulus*	VU	II
AVES	PSITTACIDAE	*Ognorhynchus icterotis*	EN	I
AVES	PSITTACIDAE	*Primolius couloni*	VU	I
AVES	PSITTACIDAE	*Prioniturus luconensis*	EN	II
AVES	PSITTACIDAE	*Prioniturus mindorensis*	VU	II
AVES	PSITTACIDAE	*Prioniturus montanus*	NT	II
AVES	PSITTACIDAE	*Prosopeia splendens*	VU	II
AVES	PSITTACIDAE	*Psittacara chloropterus*	VU	II
AVES	PSITTACIDAE	*Psittacara erythrogenys*	NT	II
AVES	PSITTACIDAE	*Psittacara euops*	VU	II

**Class**	**Family**	**Species**	**Red List category**	**CITES Appendix**
AVES	PSITTACIDAE	*Psittacara frontatus*	NT	II
AVES	PSITTACIDAE	*Psittacara wagleri*	NT	II
AVES	PSITTACIDAE	*Psittacula alexandri*	NT	II
AVES	PSITTACIDAE	*Psittacula caniceps*	NT	II
AVES	PSITTACIDAE	*Psittacula eupatria*	NT	II
AVES	PSITTACIDAE	*Psittacula finschii*	NT	II
AVES	PSITTACIDAE	*Psittacula longicauda*	VU	II
AVES	PSITTACIDAE	*Psittacula roseata*	NT	II
AVES	PSITTACIDAE	*Psittacus erithacus*	EN	I
AVES	PSITTACIDAE	*Psittacus timneh*	EN	I
AVES	PSITTACIDAE	*Psittinus cyanurus*	NT	II
AVES	PSITTACIDAE	*Psittrichas fulgidus*	VU	II
AVES	PSITTACIDAE	*Pyrilia pyrilia*	NT	II
AVES	PSITTACIDAE	*Pyrrhura chapmani*	VU	II
AVES	PSITTACIDAE	*Pyrrhura cruentata*	VU	I
AVES	PSITTACIDAE	*Pyrrhura perlata*	VU	II
AVES	PSITTACIDAE	*Rhynchopsitta pachyrhyncha*	EN	I
AVES	PSITTACIDAE	*Rhynchopsitta terrisi*	EN	I
AVES	PSITTACIDAE	*Tanygnathus lucionensis*	NT	II
AVES	PSITTACIDAE	*Trichoglossus forsteni*	VU	II
AVES	PSITTACIDAE	*Trichoglossus johnstoniae*	NT	II
AVES	PSITTACIDAE	*Trichoglossus rubiginosus*	NT	II
AVES	PSITTACIDAE	*Trichoglossus weberi*	NT	II
AVES	PSITTACIDAE	*Triclaria malachitacea*	NT	II
AVES	SPHENISCIDAE	*Eudyptes chrysocome*	VU	NC
AVES	STRIGIDAE	*Bubo blakistoni*	EN	II
AVES	ANHINGIDAE	*Anhinga melanogaster*	NT	NC
AVES	FREGATIDAE	*Fregata andrewsi*	CR	I
AVES	PHALACROCORACIDAE	*Phalacrocorax nigrogularis*	VU	NC
AVES	TROGONIDAE	*Apalharpactes reinwardtii*	VU	NC
BIVALVIA	MARGARITIFERIDAE	*Margaritifera margaritifera*	EN	NC
BIVALVIA	UNIONIDAE	*Lamprotula crassa*	CR	NC
CEPHALOPODA	OPISTHOTEUTHIDAE	*Opisthoteuthis massyae*	VU	NC
CHONDRICHTHYES	CARCHARHINIDAE	*Carcharhinus albimarginatus*	VU	NC
CHONDRICHTHYES	CARCHARHINIDAE	*Carcharhinus amblyrhynchoides*	NT	NC
CHONDRICHTHYES	CARCHARHINIDAE	*Carcharhinus brachyurus*	NT	NC
CHONDRICHTHYES	CARCHARHINIDAE	*Carcharhinus brevipinna*	NT	NC
CHONDRICHTHYES	CARCHARHINIDAE	*Carcharhinus dussumieri*	EN	NC
CHONDRICHTHYES	CARCHARHINIDAE	*Carcharhinus falciformis*	VU	II
CHONDRICHTHYES	CARCHARHINIDAE	*Carcharhinus leiodon*	EN	NC
CHONDRICHTHYES	CARCHARHINIDAE	*Carcharhinus limbatus*	NT	NC
CHONDRICHTHYES	CARCHARHINIDAE	*Carcharhinus longimanus*	CR	II
CHONDRICHTHYES	CARCHARHINIDAE	*Carcharhinus obscurus*	EN	NC
CHONDRICHTHYES	CARCHARHINIDAE	*Carcharhinus perezi*	NT	NC

Class	Family	Species	Red List category	CITES Appendix
CHONDRICHTHYES	CARCHARHINIDAE	*Carcharhinus plumbeus*	VU	NC
CHONDRICHTHYES	CARCHARHINIDAE	*Carcharhinus sorrah*	NT	NC
CHONDRICHTHYES	CARCHARHINIDAE	*Carcharhinus tjutjot*	VU	NC
CHONDRICHTHYES	CARCHARHINIDAE	*Galeocerdo cuvier*	NT	NC
CHONDRICHTHYES	CARCHARHINIDAE	*Glyphis gangeticus*	CR	NC
CHONDRICHTHYES	CARCHARHINIDAE	*Lamiopsis temminckii*	EN	NC
CHONDRICHTHYES	CARCHARHINIDAE	*Negaprion brevirostris*	NT	NC
CHONDRICHTHYES	CARCHARHINIDAE	*Prionace glauca*	NT	NC
CHONDRICHTHYES	SCYLIORHINIDAE	*Scyliorhinus capensis*	NT	NC
CHONDRICHTHYES	SPHYRNIDAE	*Eusphyra blochii*	EN	NC
CHONDRICHTHYES	SPHYRNIDAE	*Sphyrna lewini*	CR	II
CHONDRICHTHYES	SPHYRNIDAE	*Sphyrna mokarran*	CR	II
CHONDRICHTHYES	SPHYRNIDAE	*Sphyrna zygaena*	VU	II
CHONDRICHTHYES	TRIAKIDAE	*Mustelus mosis*	NT	NC
CHONDRICHTHYES	TRIAKIDAE	*Mustelus mustelus*	VU	NC
CHONDRICHTHYES	TRIAKIDAE	*Triakis megalopterus*	NT	NC
CHONDRICHTHYES	HEXANCHIDAE	*Hexanchus griseus*	NT	NC
CHONDRICHTHYES	ALOPIIDAE	*Alopias pelagicus*	EN	II
CHONDRICHTHYES	ALOPIIDAE	*Alopias superciliosus*	VU	II
CHONDRICHTHYES	ALOPIIDAE	*Alopias vulpinus*	VU	II
CHONDRICHTHYES	CETORHINIDAE	*Cetorhinus maximus*	EN	II
CHONDRICHTHYES	LAMNIDAE	*Carcharodon carcharias*	VU	II
CHONDRICHTHYES	LAMNIDAE	*Isurus oxyrinchus*	EN	II
CHONDRICHTHYES	LAMNIDAE	*Isurus paucus*	EN	II
CHONDRICHTHYES	LAMNIDAE	*Lamna nasus*	VU	II
CHONDRICHTHYES	ODONTASPIDIDAE	*Carcharias taurus*	VU	NC
CHONDRICHTHYES	AETOBATIDAE	*Aetobatus flagellum*	EN	NC
CHONDRICHTHYES	AETOBATIDAE	*Aetobatus ocellatus*	VU	NC
CHONDRICHTHYES	DASYATIDAE	*Fontitrygon colarensis*	VU	NC
CHONDRICHTHYES	DASYATIDAE	*Hemitrygon laosensis*	EN	NC
CHONDRICHTHYES	DASYATIDAE	*Himantura uarnak*	VU	NC
CHONDRICHTHYES	DASYATIDAE	*Himantura undulata*	VU	NC
CHONDRICHTHYES	DASYATIDAE	*Maculabatis arabica*	CR	NC
CHONDRICHTHYES	DASYATIDAE	*Maculabatis gerrardi*	VU	NC
CHONDRICHTHYES	DASYATIDAE	*Maculabatis pastinacoides*	VU	NC
CHONDRICHTHYES	DASYATIDAE	*Pastinachus sephen*	NT	NC
CHONDRICHTHYES	DASYATIDAE	*Pastinachus solocirostris*	EN	NC
CHONDRICHTHYES	DASYATIDAE	*Pateobatis fai*	VU	NC
CHONDRICHTHYES	DASYATIDAE	*Pateobatis uarnacoides*	VU	NC
CHONDRICHTHYES	DASYATIDAE	*Urogymnus asperrimus*	VU	NC
CHONDRICHTHYES	DASYATIDAE	*Urogymnus granulatus*	VU	NC
CHONDRICHTHYES	DASYATIDAE	*Urogymnus polylepis*	EN	NC
CHONDRICHTHYES	MOBULIDAE	*Mobula alfredi*	VU	II
CHONDRICHTHYES	MOBULIDAE	*Mobula birostris*	VU	II
CHONDRICHTHYES	MOBULIDAE	*Mobula hypostoma*	EN	II

Class	Family	Species	Red List category	CITES Appendix
CHONDRICHTHYES	MOBULIDAE	*Mobula mobular*	EN	II
CHONDRICHTHYES	MOBULIDAE	*Mobula munkiana*	VU	II
CHONDRICHTHYES	MOBULIDAE	*Mobula tarapacana*	EN	II
CHONDRICHTHYES	MOBULIDAE	*Mobula thurstoni*	EN	II
CHONDRICHTHYES	MYLIOBATIDAE	*Aetomylaeus nichofii*	VU	NC
CHONDRICHTHYES	MYLIOBATIDAE	*Myliobatis hamlyni*	NT	NC
CHONDRICHTHYES	POTAMOTRYGONIDAE	*Potamotrygon tigrina*	EN	NC
CHONDRICHTHYES	RHINOPTERIDAE	*Rhinoptera marginata*	NT	NC
CHONDRICHTHYES	HEMISCYLLIIDAE	*Chiloscyllium hasselti*	NT	NC
CHONDRICHTHYES	HEMISCYLLIIDAE	*Chiloscyllium plagiosum*	NT	NC
CHONDRICHTHYES	RHINCODONTIDAE	*Rhincodon typus*	EN	II
CHONDRICHTHYES	STEGOSTOMIDAE	*Stegostoma tigrinum*	EN	NC
CHONDRICHTHYES	ARHYNCHOBATIDAE	*Atlantoraja cyclophora*	VU	NC
CHONDRICHTHYES	ARHYNCHOBATIDAE	*Atlantoraja platana*	VU	NC
CHONDRICHTHYES	ARHYNCHOBATIDAE	*Bathyraja albomaculata*	VU	NC
CHONDRICHTHYES	ARHYNCHOBATIDAE	*Bathyraja cousseauae*	NT	NC
CHONDRICHTHYES	ARHYNCHOBATIDAE	*Bathyraja griseocauda*	EN	NC
CHONDRICHTHYES	ARHYNCHOBATIDAE	*Bathyraja macloviana*	NT	NC
CHONDRICHTHYES	ARHYNCHOBATIDAE	*Bathyraja multispinis*	NT	NC
CHONDRICHTHYES	ARHYNCHOBATIDAE	*Bathyraja scaphiops*	NT	NC
CHONDRICHTHYES	ARHYNCHOBATIDAE	*Bathyraja spinicauda*	NT	NC
CHONDRICHTHYES	ARHYNCHOBATIDAE	*Sympterygia acuta*	VU	NC
CHONDRICHTHYES	RAJIDAE	*Amblyraja radiata*	VU	NC
CHONDRICHTHYES	RAJIDAE	*Dipturus batis*	CR	NC
CHONDRICHTHYES	RAJIDAE	*Dipturus laevis*	EN	NC
CHONDRICHTHYES	RAJIDAE	*Dipturus mennii*	VU	NC
CHONDRICHTHYES	RAJIDAE	*Dipturus trachyderma*	VU	NC
CHONDRICHTHYES	RAJIDAE	*Leucoraja erinacea*	NT	NC
CHONDRICHTHYES	RAJIDAE	*Leucoraja ocellata*	EN	NC
CHONDRICHTHYES	RAJIDAE	*Raja brachyura*	NT	NC
CHONDRICHTHYES	RAJIDAE	*Raja clavata*	NT	NC
CHONDRICHTHYES	RAJIDAE	*Raja undulata*	EN	NC
CHONDRICHTHYES	RAJIDAE	*Dipturus chilensis*	EN	NC
CHONDRICHTHYES	RAJIDAE	*Dipturus brevicaudatus*	VU	NC
CHONDRICHTHYES	GLAUCOSTEGIDAE	*Glaucostegus cemiculus*	CR	II
CHONDRICHTHYES	GLAUCOSTEGIDAE	*Glaucostegus granulatus*	CR	II
CHONDRICHTHYES	GLAUCOSTEGIDAE	*Glaucostegus halavi*	CR	II
CHONDRICHTHYES	GLAUCOSTEGIDAE	*Glaucostegus obtusus*	CR	II
CHONDRICHTHYES	GLAUCOSTEGIDAE	*Glaucostegus thouin*	CR	II
CHONDRICHTHYES	GLAUCOSTEGIDAE	*Glaucostegus typus*	CR	II
CHONDRICHTHYES	PRISTIDAE	*Anoxypristis cuspidata*	EN	I
CHONDRICHTHYES	PRISTIDAE	*Pristis pectinata*	CR	I
CHONDRICHTHYES	PRISTIDAE	*Pristis pristis*	CR	I
CHONDRICHTHYES	RHINIDAE	*Rhina ancylostoma*	CR	II
CHONDRICHTHYES	RHINIDAE	*Rhynchobatus australiae*	CR	II

**Class**	**Family**	**Species**	**Red List category**	**CITES Appendix**
CHONDRICHTHYES	RHINIDAE	*Rhynchobatus cooki*	CR	II
CHONDRICHTHYES	RHINIDAE	*Rhynchobatus djiddensis*	CR	II
CHONDRICHTHYES	RHINIDAE	*Rhynchobatus immaculatus*	CR	II
CHONDRICHTHYES	RHINIDAE	*Rhynchobatus laevis*	CR	II
CHONDRICHTHYES	RHINIDAE	*Rhynchobatus luebberti*	CR	II
CHONDRICHTHYES	RHINIDAE	*Rhynchobatus palpebratus*	NT	II
CHONDRICHTHYES	RHINIDAE	*Rhynchobatus springeri*	CR	II
CHONDRICHTHYES	RHINIDAE	*Rhynchorhina mauritaniensis*	CR	II
CHONDRICHTHYES	RHINOBATIDAE	*Acroteriobatus leucospilus*	EN	NC
CHONDRICHTHYES	RHINOBATIDAE	*Acroteriobatus salalah*	NT	NC
CHONDRICHTHYES	RHINOBATIDAE	*Acroteriobatus variegatus*	CR	NC
CHONDRICHTHYES	RHINOBATIDAE	*Acroteriobatus zanzibarensis*	NT	NC
CHONDRICHTHYES	RHINOBATIDAE	*Pseudobatos percellens*	NT	NC
CHONDRICHTHYES	RHINOBATIDAE	*Rhinobatos albomaculatus*	VU	NC
CHONDRICHTHYES	RHINOBATIDAE	*Rhinobatos irvinei*	VU	NC
CHONDRICHTHYES	RHINOBATIDAE	*Rhinobatos punctifer*	NT	NC
CHONDRICHTHYES	RHINOBATIDAE	*Rhinobatos rhinobatos*	EN	NC
CHONDRICHTHYES	DALATIIDAE	*Dalatias licha*	VU	NC
CHONDRICHTHYES	SQUALIDAE	*Squalus acanthias*	VU	NC
CHONDRICHTHYES	SQUALIDAE	*Squalus edmundsi*	NT	NC
CHONDRICHTHYES	SQUALIDAE	*Squalus hemipinnis*	NT	NC
CHONDRICHTHYES	SQUALIDAE	*Squalus nasutus*	NT	NC
CHONDRICHTHYES	SQUATINIDAE	*Squatina aculeata*	CR	NC
CHONDRICHTHYES	SQUATINIDAE	*Squatina africana*	NT	NC
CHONDRICHTHYES	SQUATINIDAE	*Squatina argentina*	CR	NC
CHONDRICHTHYES	SQUATINIDAE	*Squatina david*	NT	NC
CHONDRICHTHYES	SQUATINIDAE	*Squatina japonica*	VU	NC
CHONDRICHTHYES	SQUATINIDAE	*Squatina oculata*	CR	NC
CYCADOPSIDA	CYCADACEAE	*Cycas bifida*	VU	II
CYCADOPSIDA	CYCADACEAE	*Cycas changjiangensis*	EN	II
CYCADOPSIDA	CYCADACEAE	*Cycas chevalieri*	NT	II
CYCADOPSIDA	CYCADACEAE	*Cycas couttsiana*	NT	II
CYCADOPSIDA	CYCADACEAE	*Cycas debaoensis*	CR	II
CYCADOPSIDA	CYCADACEAE	*Cycas diannanensis*	VU	II
CYCADOPSIDA	CYCADACEAE	*Cycas dolichophylla*	NT	II
CYCADOPSIDA	CYCADACEAE	*Cycas elephantipes*	EN	II
CYCADOPSIDA	CYCADACEAE	*Cycas elongata*	EN	II
CYCADOPSIDA	CYCADACEAE	*Cycas guizhouensis*	VU	II
CYCADOPSIDA	CYCADACEAE	*Cycas hainanensis*	EN	II
CYCADOPSIDA	CYCADACEAE	*Cycas hoabinhensis*	EN	II
CYCADOPSIDA	CYCADACEAE	*Cycas hongheensis*	CR	II
CYCADOPSIDA	CYCADACEAE	*Cycas multipinnata*	EN	II
CYCADOPSIDA	CYCADACEAE	*Cycas nongnoochiae*	VU	II
CYCADOPSIDA	CYCADACEAE	*Cycas ophiolitica*	VU	II
CYCADOPSIDA	CYCADACEAE	*Cycas pachypoda*	CR	II

Class	Family	Species	Red List category	CITES Appendix
CYCADOPSIDA	CYCADACEAE	*Cycas panzhihuaensis*	VU	II
CYCADOPSIDA	CYCADACEAE	*Cycas platyphylla*	EN	II
CYCADOPSIDA	CYCADACEAE	*Cycas riuminiana*	EN	II
CYCADOPSIDA	CYCADACEAE	*Cycas seemannii*	VU	II
CYCADOPSIDA	CYCADACEAE	*Cycas sexseminifera*	NT	II
CYCADOPSIDA	CYCADACEAE	*Cycas siamensis*	VU	II
CYCADOPSIDA	CYCADACEAE	*Cycas taitungensis*	EN	II
CYCADOPSIDA	CYCADACEAE	*Cycas tansachana*	CR	II
CYCADOPSIDA	ZAMIACEAE	*Ceratozamia alvarezii*	EN	I
CYCADOPSIDA	ZAMIACEAE	*Ceratozamia becerrae*	EN	I
CYCADOPSIDA	ZAMIACEAE	*Ceratozamia hildae*	EN	I
CYCADOPSIDA	ZAMIACEAE	*Ceratozamia huastecorum*	CR	I
CYCADOPSIDA	ZAMIACEAE	*Ceratozamia kuesteriana*	CR	I
CYCADOPSIDA	ZAMIACEAE	*Ceratozamia latifolia*	EN	I
CYCADOPSIDA	ZAMIACEAE	*Ceratozamia miqueliana*	CR	I
CYCADOPSIDA	ZAMIACEAE	*Ceratozamia norstogii*	EN	I
CYCADOPSIDA	ZAMIACEAE	*Ceratozamia robusta*	EN	I
CYCADOPSIDA	ZAMIACEAE	*Ceratozamia zaragozae*	CR	I
CYCADOPSIDA	ZAMIACEAE	*Ceratozamia zoquorum*	CR	I
CYCADOPSIDA	ZAMIACEAE	*Dioon califanoi*	EN	II
CYCADOPSIDA	ZAMIACEAE	*Dioon edule*	NT	II
CYCADOPSIDA	ZAMIACEAE	*Dioon holmgrenii*	EN	II
CYCADOPSIDA	ZAMIACEAE	*Dioon sonorense*	EN	II
CYCADOPSIDA	ZAMIACEAE	*Dioon spinulosum*	EN	II
CYCADOPSIDA	ZAMIACEAE	*Dioon tomasellii*	VU	II
CYCADOPSIDA	ZAMIACEAE	*Encephalartos aemulans*	CR	I
CYCADOPSIDA	ZAMIACEAE	*Encephalartos aplanatus*	VU	I
CYCADOPSIDA	ZAMIACEAE	*Encephalartos arenarius*	EN	I
CYCADOPSIDA	ZAMIACEAE	*Encephalartos barteri*	VU	I
CYCADOPSIDA	ZAMIACEAE	*Encephalartos caffer*	NT	I
CYCADOPSIDA	ZAMIACEAE	*Encephalartos cerinus*	CR	I
CYCADOPSIDA	ZAMIACEAE	*Encephalartos chimanimaniensis*	EN	I
CYCADOPSIDA	ZAMIACEAE	*Encephalartos delucanus*	EN	I
CYCADOPSIDA	ZAMIACEAE	*Encephalartos dolomiticus*	CR	I
CYCADOPSIDA	ZAMIACEAE	*Encephalartos dyerianus*	CR	I
CYCADOPSIDA	ZAMIACEAE	*Encephalartos equatorialis*	CR	I
CYCADOPSIDA	ZAMIACEAE	*Encephalartos eugene-maraisii*	EN	I
CYCADOPSIDA	ZAMIACEAE	*Encephalartos ferox*	NT	I
CYCADOPSIDA	ZAMIACEAE	*Encephalartos friderici-guilielmi*	NT	I
CYCADOPSIDA	ZAMIACEAE	*Encephalartos ghellinckii*	VU	I
CYCADOPSIDA	ZAMIACEAE	*Encephalartos hirsutus*	CR	I
CYCADOPSIDA	ZAMIACEAE	*Encephalartos horridus*	EN	I
CYCADOPSIDA	ZAMIACEAE	*Encephalartos humilis*	VU	I

Class	Family	Species	Red List category	CITES Appendix
CYCADOPSIDA	ZAMIACEAE	*Encephalartos inopinus*	CR	I
CYCADOPSIDA	ZAMIACEAE	*Encephalartos kanga*	CR	I
CYCADOPSIDA	ZAMIACEAE	*Encephalartos kisambo*	EN	I
CYCADOPSIDA	ZAMIACEAE	*Encephalartos latifrons*	CR	I
CYCADOPSIDA	ZAMIACEAE	*Encephalartos laurentianus*	NT	I
CYCADOPSIDA	ZAMIACEAE	*Encephalartos lehmannii*	NT	I
CYCADOPSIDA	ZAMIACEAE	*Encephalartos longifolius*	NT	I
CYCADOPSIDA	ZAMIACEAE	*Encephalartos mackenziei*	NT	I
CYCADOPSIDA	ZAMIACEAE	*Encephalartos macrostrobilus*	EN	I
CYCADOPSIDA	ZAMIACEAE	*Encephalartos manikensis*	VU	I
CYCADOPSIDA	ZAMIACEAE	*Encephalartos marunguensis*	VU	I
CYCADOPSIDA	ZAMIACEAE	*Encephalartos middelburgensis*	CR	I
CYCADOPSIDA	ZAMIACEAE	*Encephalartos msinganus*	CR	I
CYCADOPSIDA	ZAMIACEAE	*Encephalartos munchii*	CR	I
CYCADOPSIDA	ZAMIACEAE	*Encephalartos ngoyanus*	VU	I
CYCADOPSIDA	ZAMIACEAE	*Encephalartos paucidentatus*	VU	I
CYCADOPSIDA	ZAMIACEAE	*Encephalartos princeps*	VU	I
CYCADOPSIDA	ZAMIACEAE	*Encephalartos pterogonus*	CR	I
CYCADOPSIDA	ZAMIACEAE	*Encephalartos schmitzii*	VU	I
CYCADOPSIDA	ZAMIACEAE	*Encephalartos sclavoi*	CR	I
CYCADOPSIDA	ZAMIACEAE	*Encephalartos trispinosus*	VU	I
CYCADOPSIDA	ZAMIACEAE	*Encephalartos umbeluziensis*	EN	I
CYCADOPSIDA	ZAMIACEAE	*Macrozamia conferta*	VU	II
CYCADOPSIDA	ZAMIACEAE	*Macrozamia fawcettii*	NT	II
CYCADOPSIDA	ZAMIACEAE	*Macrozamia flexuosa*	EN	II
CYCADOPSIDA	ZAMIACEAE	*Macrozamia lomandroides*	EN	II
CYCADOPSIDA	ZAMIACEAE	*Macrozamia machinii*	VU	II
CYCADOPSIDA	ZAMIACEAE	*Macrozamia occidua*	VU	II
CYCADOPSIDA	ZAMIACEAE	*Macrozamia parcifolia*	VU	II
CYCADOPSIDA	ZAMIACEAE	*Macrozamia pauli-guilielmi*	EN	II
CYCADOPSIDA	ZAMIACEAE	*Macrozamia stenomera*	NT	II
CYCADOPSIDA	ZAMIACEAE	*Microcycas calocoma*	CR	I
CYCADOPSIDA	ZAMIACEAE	*Zamia amazonum*	NT	II
CYCADOPSIDA	ZAMIACEAE	*Zamia cremnophila*	EN	II
CYCADOPSIDA	ZAMIACEAE	*Zamia cunaria*	VU	II
CYCADOPSIDA	ZAMIACEAE	*Zamia encephalartoides*	VU	II
CYCADOPSIDA	ZAMIACEAE	*Zamia erosa*	VU	II
CYCADOPSIDA	ZAMIACEAE	*Zamia fischeri*	EN	II
CYCADOPSIDA	ZAMIACEAE	*Zamia furfuracea*	EN	II
CYCADOPSIDA	ZAMIACEAE	*Zamia katzeriana*	EN	II
CYCADOPSIDA	ZAMIACEAE	*Zamia manicata*	NT	II
CYCADOPSIDA	ZAMIACEAE	*Zamia pygmaea*	CR	II
CYCADOPSIDA	ZAMIACEAE	*Zamia restrepoi*	CR	I
CYCADOPSIDA	ZAMIACEAE	*Zamia skinneri*	EN	II

Class	Family	Species	Red List category	CITES Appendix
CYCADOPSIDA	ZAMIACEAE	*Zamia soconuscensis*	VU	II
CYCADOPSIDA	ZAMIACEAE	*Zamia vazquezii*	CR	II
GASTROPODA	HALIOTIDAE	*Haliotis kamtschatkana*	EN	NC
GASTROPODA	CONIDAE	*Conus anabathrum*	VU	NC
GASTROPODA	CONIDAE	*Conus aurantius*	NT	NC
GASTROPODA	CONIDAE	*Conus cuneolus*	EN	NC
GASTROPODA	CONIDAE	*Conus denizi*	NT	NC
GASTROPODA	CONIDAE	*Conus gauguini*	NT	NC
GASTROPODA	CONIDAE	*Conus guinaicus*	VU	NC
GASTROPODA	CONIDAE	*Conus immelmani*	VU	NC
GASTROPODA	CONIDAE	*Conus julii*	VU	NC
GASTROPODA	CONIDAE	*Conus kersteni*	NT	NC
GASTROPODA	CONIDAE	*Conus mercator*	EN	NC
GASTROPODA	CONIDAE	*Conus nobrei*	NT	NC
GASTROPODA	CONIDAE	*Conus richardbinghami*	VU	NC
GASTROPODA	CONIDAE	*Conus thevenardensis*	VU	NC
GASTROPODA	CONIDAE	*Conus trochulus*	NT	NC
GASTROPODA	MELANOPSIDAE	*Melanopsis chlorotica*	CR	NC
GASTROPODA	MELANOPSIDAE	*Melanopsis letourneuxi*	EN	NC
GASTROPODA	MELANOPSIDAE	*Melanopsis magnifica*	EN	NC
GASTROPODA	MELANOPSIDAE	*Melanopsis mourebeyensis*	EN	NC
GASTROPODA	MELANOPSIDAE	*Melanopsis saharica*	CR	NC
GASTROPODA	MELANOPSIDAE	*Melanopsis scalaris*	EN	NC
GASTROPODA	MELANOPSIDAE	*Melanopsis subgraellsiana*	VU	NC
GASTROPODA	PACHYCHILIDAE	*Tylomelania amphiderita*	EN	NC
GASTROPODA	PACHYCHILIDAE	*Tylomelania gemmifera*	EN	NC
GASTROPODA	PACHYCHILIDAE	*Tylomelania patriarchalis*	EN	NC
GASTROPODA	PACHYCHILIDAE	*Tylomelania sinabartfeldi*	CR	NC
GASTROPODA	PACHYCHILIDAE	*Tylomelania towutensis*	EN	NC
GASTROPODA	PACHYCHILIDAE	*Tylomelania towutica*	EN	NC
GASTROPODA	CAMAENIDAE	*Papustyla pulcherrima*	NT	II
GASTROPODA	CHONDRINIDAE	*Chondrina feneriensis*	NT	NC
GASTROPODA	CLAUSILIIDAE	*Bofilliella subarcuata*	NT	NC
GASTROPODA	CLAUSILIIDAE	*Charpentieria crassicostata*	CR	NC
GASTROPODA	CLAUSILIIDAE	*Charpentieria eminens*	EN	NC
GASTROPODA	CLAUSILIIDAE	*Charpentieria ferrox*	CR	NC
GASTROPODA	CLAUSILIIDAE	*Charpentieria grohmanniana*	EN	NC
GASTROPODA	CLAUSILIIDAE	*Charpentieria leucophryna*	EN	NC
GASTROPODA	CLAUSILIIDAE	*Charpentieria nobilis*	EN	NC
GASTROPODA	CLAUSILIIDAE	*Charpentieria septemplicata*	NT	NC
GASTROPODA	CLAUSILIIDAE	*Charpentieria spezialensis*	CR	NC
GASTROPODA	CLAUSILIIDAE	*Charpentieria tiberii*	NT	NC
GASTROPODA	CLAUSILIIDAE	*Macedonica zilchi*	VU	NC
GASTROPODA	FERUSSACIIDAE	*Cecilioides clessini*	NT	NC
GASTROPODA	OXYCHILIDAE	*Oxychilus basajauna*	EN	NC

**Class**	**Family**	**Species**	**Red List category**	**CITES Appendix**
GNETOPSIDA	GNETACEAE	*Gnetum africanum*	NT	NC
GNETOPSIDA	GNETACEAE	*Gnetum buchholzianum*	NT	NC
HOLOTHUROIDEA	HOLOTHURIIDAE	*Actinopyga echinites*	VU	NC
HOLOTHUROIDEA	HOLOTHURIIDAE	*Actinopyga mauritiana*	VU	NC
HOLOTHUROIDEA	HOLOTHURIIDAE	*Actinopyga miliaris*	VU	NC
HOLOTHUROIDEA	HOLOTHURIIDAE	*Holothuria fuscogilva*	VU	II
HOLOTHUROIDEA	HOLOTHURIIDAE	*Holothuria lessoni*	EN	NC
HOLOTHUROIDEA	HOLOTHURIIDAE	*Holothuria nobilis*	EN	II
HOLOTHUROIDEA	HOLOTHURIIDAE	*Holothuria scabra*	EN	NC
HOLOTHUROIDEA	HOLOTHURIIDAE	*Holothuria whitmaei*	EN	II
HOLOTHUROIDEA	STICHOPODIDAE	*Apostichopus japonicus*	EN	NC
HOLOTHUROIDEA	STICHOPODIDAE	*Isostichopus fuscus*	EN	III
HOLOTHUROIDEA	STICHOPODIDAE	*Thelenota ananas*	EN	NC
HYDROZOA	MILLEPORIDAE	*Millepora boschmai*	CR	II
HYDROZOA	MILLEPORIDAE	*Millepora foveolata*	VU	II
HYDROZOA	MILLEPORIDAE	*Millepora latifolia*	VU	II
HYDROZOA	MILLEPORIDAE	*Millepora murrayi*	NT	II
HYDROZOA	MILLEPORIDAE	*Millepora striata*	EN	II
HYDROZOA	MILLEPORIDAE	*Millepora tuberosa*	EN	II
INSECTA	CERAMBYCIDAE	*Pseudogaurotina excellens*	EN	NC
INSECTA	GEOTRUPIDAE	*Ceratophyus martinezi*	EN	NC
INSECTA	GEOTRUPIDAE	*Ceratophyus rossii*	EN	NC
INSECTA	PAPILIONIDAE	*Bhutanitis thaidina*	NT	II
INSECTA	PAPILIONIDAE	*Ornithoptera aesacus*	VU	II
INSECTA	PAPILIONIDAE	*Ornithoptera alexandrae*	EN	I
INSECTA	PAPILIONIDAE	*Ornithoptera croesus*	NT	II
INSECTA	PAPILIONIDAE	*Ornithoptera meridionalis*	NT	II
INSECTA	PAPILIONIDAE	*Ornithoptera paradisea*	NT	II
INSECTA	PAPILIONIDAE	*Ornithoptera rothschildi*	NT	II
INSECTA	PAPILIONIDAE	*Pachliopta jophon*	EN	II
INSECTA	PAPILIONIDAE	*Trogonoptera trojana*	NT	II
INSECTA	PAPILIONIDAE	*Troides andromache*	VU	II
INSECTA	PAPILIONIDAE	*Troides dohertyi*	VU	II
INSECTA	TETTIGONIIDAE	*Poecilimon heinrichi*	NT	NC
LILIOPSIDA	ARACEAE	*Alocasia sanderiana*	CR	NC
LILIOPSIDA	ARACEAE	*Amorphophallus curvistylis*	VU	NC
LILIOPSIDA	ARACEAE	*Anthurium magnificum*	NT	NC
LILIOPSIDA	ARACEAE	*Arum purpureospathum*	VU	NC
LILIOPSIDA	ARACEAE	*Biarum davisii*	NT	NC
LILIOPSIDA	ARACEAE	*Philodendron gloriosum*	VU	NC
LILIOPSIDA	ARECACEAE	*Basselinia deplanchei*	NT	NC
LILIOPSIDA	ARECACEAE	*Basselinia moorei*	CR	NC
LILIOPSIDA	ARECACEAE	*Basselinia porphyrea*	EN	NC
LILIOPSIDA	ARECACEAE	*Beccariophoenix alfredii*	VU	NC
LILIOPSIDA	ARECACEAE	*Burretiokentia koghiensis*	CR	NC

Class	Family	Species	Red List category	CITES Appendix
LILIOPSIDA	ARECACEAE	*Chamaedorea klotzschiana*	EN	NC
LILIOPSIDA	ARECACEAE	*Chamaedorea oblongata*	VU	NC
LILIOPSIDA	ARECACEAE	*Clinosperma macrocarpa*	CR	NC
LILIOPSIDA	ARECACEAE	*Clinosperma vaginata*	NT	NC
LILIOPSIDA	ARECACEAE	*Cyphophoenix nucele*	VU	NC
LILIOPSIDA	ARECACEAE	*Dypsis albofarinosa*	CR	NC
LILIOPSIDA	ARECACEAE	*Dypsis ambositrae*	CR	NC
LILIOPSIDA	ARECACEAE	*Dypsis ampasindavae*	CR	NC
LILIOPSIDA	ARECACEAE	*Dypsis antanambensis*	CR	NC
LILIOPSIDA	ARECACEAE	*Dypsis arenarum*	CR	NC
LILIOPSIDA	ARECACEAE	*Dypsis basilonga*	CR	NC
LILIOPSIDA	ARECACEAE	*Dypsis carlsmithii*	CR	NC
LILIOPSIDA	ARECACEAE	*Dypsis ceracea*	EN	NC
LILIOPSIDA	ARECACEAE	*Dypsis decaryi*	VU	II
LILIOPSIDA	ARECACEAE	*Dypsis decipiens*	VU	I
LILIOPSIDA	ARECACEAE	*Dypsis hovomantsina*	CR	NC
LILIOPSIDA	ARECACEAE	*Dypsis ifanadianae*	CR	NC
LILIOPSIDA	ARECACEAE	*Dypsis leptocheilos*	CR	NC
LILIOPSIDA	ARECACEAE	*Dypsis malcomberi*	EN	NC
LILIOPSIDA	ARECACEAE	*Dypsis mananjarensis*	NT	NC
LILIOPSIDA	ARECACEAE	*Dypsis onilahensis*	VU	NC
LILIOPSIDA	ARECACEAE	*Dypsis pilulifera*	VU	NC
LILIOPSIDA	ARECACEAE	*Dypsis tokoravina*	CR	NC
LILIOPSIDA	ARECACEAE	*Dypsis tsaravoasira*	VU	NC
LILIOPSIDA	ARECACEAE	*Lemurophoenix halleuxii*	EN	II
LILIOPSIDA	ARECACEAE	*Marojejya darianii*	EN	II
LILIOPSIDA	ARECACEAE	*Masoala madagascariensis*	CR	NC
LILIOPSIDA	ARECACEAE	*Parajubaea sunkha*	EN	NC
LILIOPSIDA	ARECACEAE	*Phytelephas seemannii*	LR/cd	NC
LILIOPSIDA	ARECACEAE	*Phytelephas tumacana*	EN	NC
LILIOPSIDA	ARECACEAE	*Ravenea musicalis*	CR	NC
LILIOPSIDA	ARECACEAE	*Ravenea rivularis*	VU	II
LILIOPSIDA	ARECACEAE	*Saribus jeanneneyi*	CR	NC
LILIOPSIDA	ARECACEAE	*Satranala decussilvae*	EN	II
LILIOPSIDA	ARECACEAE	*Voanioala gerardii*	CR	II
LILIOPSIDA	ARECACEAE	*Washingtonia filifera*	LR/nt	NC
LILIOPSIDA	AMARYLLIDACEAE	*Acis nicaeensis*	EN	NC
LILIOPSIDA	AMARYLLIDACEAE	*Caliphruria hartwegiana*	EN	NC
LILIOPSIDA	AMARYLLIDACEAE	*Crinum thaianum*	EN	NC
LILIOPSIDA	AMARYLLIDACEAE	*Galanthus nivalis*	NT	II
LILIOPSIDA	AMARYLLIDACEAE	*Galanthus peshmenii*	VU	II
LILIOPSIDA	AMARYLLIDACEAE	*Galanthus trojanus*	CR	II
LILIOPSIDA	AMARYLLIDACEAE	*Narcissus gaditanus*	NT	NC
LILIOPSIDA	AMARYLLIDACEAE	*Narcissus jeanmonodii*	EN	NC
LILIOPSIDA	AMARYLLIDACEAE	*Narcissus lusitanicus*	VU	NC

Class	Family	Species	Red List category	CITES Appendix
LILIOPSIDA	AMARYLLIDACEAE	*Narcissus nevadensis*	EN	NC
LILIOPSIDA	AMARYLLIDACEAE	*Narcissus viridiflorus*	NT	NC
LILIOPSIDA	AMARYLLIDACEAE	*Narcissus willkommii*	EN	NC
LILIOPSIDA	AMARYLLIDACEAE	*Phaedranassa viridiflora*	EN	NC
LILIOPSIDA	AMARYLLIDACEAE	*Worsleya procera*	CR	NC
LILIOPSIDA	ASPARAGACEAE	*Agave albopilosa*	CR	NC
LILIOPSIDA	ASPARAGACEAE	*Agave bovicornuta*	VU	NC
LILIOPSIDA	ASPARAGACEAE	*Agave cupreata*	EN	NC
LILIOPSIDA	ASPARAGACEAE	*Agave guiengola*	EN	NC
LILIOPSIDA	ASPARAGACEAE	*Agave impressa*	EN	NC
LILIOPSIDA	ASPARAGACEAE	*Agave isthmensis*	VU	NC
LILIOPSIDA	ASPARAGACEAE	*Agave kavandivi*	CR	NC
LILIOPSIDA	ASPARAGACEAE	*Agave macroacantha*	EN	NC
LILIOPSIDA	ASPARAGACEAE	*Agave parrasana*	VU	NC
LILIOPSIDA	ASPARAGACEAE	*Agave pelona*	CR	NC
LILIOPSIDA	ASPARAGACEAE	*Agave petrophila*	EN	NC
LILIOPSIDA	ASPARAGACEAE	*Agave pintilla*	CR	NC
LILIOPSIDA	ASPARAGACEAE	*Agave potatorum*	VU	NC
LILIOPSIDA	ASPARAGACEAE	*Agave potrerana*	VU	NC
LILIOPSIDA	ASPARAGACEAE	*Agave titanota*	EN	NC
LILIOPSIDA	ASPARAGACEAE	*Agave warelliana*	EN	NC
LILIOPSIDA	ASPARAGACEAE	*Agave zebra*	VU	NC
LILIOPSIDA	ASPARAGACEAE	*Beaucarnea goldmanii*	VU	II
LILIOPSIDA	ASPARAGACEAE	*Beaucarnea gracilis*	EN	II
LILIOPSIDA	ASPARAGACEAE	*Beaucarnea hiriartiae*	CR	II
LILIOPSIDA	ASPARAGACEAE	*Beaucarnea pliabilis*	VU	II
LILIOPSIDA	ASPARAGACEAE	*Beaucarnea purpusii*	EN	II
LILIOPSIDA	ASPARAGACEAE	*Beaucarnea recurvata*	CR	II
LILIOPSIDA	ASPARAGACEAE	*Beaucarnea sanctomariana*	EN	II
LILIOPSIDA	ASPARAGACEAE	*Beaucarnea stricta*	VU	II
LILIOPSIDA	ASPARAGACEAE	*Chlorophytum borivilianum*	CR	NC
LILIOPSIDA	ASPARAGACEAE	*Yucca linearifolia*	VU	NC
LILIOPSIDA	ASPHODELACEAE	*Aloe classenii*	CR	II
LILIOPSIDA	ASPHODELACEAE	*Aloe flexilifolia*	CR	II
LILIOPSIDA	ASPHODELACEAE	*Aloe ketabrowniorum*	EN	II
LILIOPSIDA	ASPHODELACEAE	*Aloe kilifiensis*	EN	II
LILIOPSIDA	ASPHODELACEAE	*Aloe leptosiphon*	CR	II
LILIOPSIDA	ASPHODELACEAE	*Aloe massawana*	VU	II
LILIOPSIDA	ASPHODELACEAE	*Aloe ukambensis*	VU	II
LILIOPSIDA	IRIDACEAE	*Iris atropurpurea*	CR	NC
LILIOPSIDA	IRIDACEAE	*Iris nusairiensis*	CR	NC
LILIOPSIDA	IRIDACEAE	*Iris orjenii*	VU	NC
LILIOPSIDA	IRIDACEAE	*Moraea callista*	VU	NC
LILIOPSIDA	ORCHIDACEAE	*Aerangis macrocentra*	NT	II
LILIOPSIDA	ORCHIDACEAE	*Aeranthes albidiflora*	CR	II

Class	Family	Species	Red List category	CITES Appendix
LILIOPSIDA	ORCHIDACEAE	*Aeranthes antennophora*	EN	II
LILIOPSIDA	ORCHIDACEAE	*Aeranthes multinodis*	CR	II
LILIOPSIDA	ORCHIDACEAE	*Aeranthes neoperrieri*	EN	II
LILIOPSIDA	ORCHIDACEAE	*Aeranthes setipes*	EN	II
LILIOPSIDA	ORCHIDACEAE	*Aeranthes strangulata*	VU	II
LILIOPSIDA	ORCHIDACEAE	*Altensteinia longispicata*	VU	II
LILIOPSIDA	ORCHIDACEAE	*Amesiella monticola*	CR	II
LILIOPSIDA	ORCHIDACEAE	*Amesiella philippensis*	EN	II
LILIOPSIDA	ORCHIDACEAE	*Anathallis tigridens*	EN	II
LILIOPSIDA	ORCHIDACEAE	*Angraecum humbertii*	EN	II
LILIOPSIDA	ORCHIDACEAE	*Angraecum mahavavense*	CR	II
LILIOPSIDA	ORCHIDACEAE	*Angraecum muscicolum*	CR	II
LILIOPSIDA	ORCHIDACEAE	*Angraecum obesum*	EN	II
LILIOPSIDA	ORCHIDACEAE	*Angraecum palmicolum*	CR	II
LILIOPSIDA	ORCHIDACEAE	*Angraecum perhumile*	CR	II
LILIOPSIDA	ORCHIDACEAE	*Angraecum peyrotii*	CR	II
LILIOPSIDA	ORCHIDACEAE	*Angraecum pinifolium*	EN	II
LILIOPSIDA	ORCHIDACEAE	*Angraecum potamophilum*	CR	II
LILIOPSIDA	ORCHIDACEAE	*Angraecum protensum*	EN	II
LILIOPSIDA	ORCHIDACEAE	*Angraecum rigidifolium*	CR	II
LILIOPSIDA	ORCHIDACEAE	*Angraecum rubellum*	CR	II
LILIOPSIDA	ORCHIDACEAE	*Angraecum sambiranoense*	EN	II
LILIOPSIDA	ORCHIDACEAE	*Angraecum setipes*	VU	II
LILIOPSIDA	ORCHIDACEAE	*Anguloa cliftonii*	CR	II
LILIOPSIDA	ORCHIDACEAE	*Ansellia africana*	VU	II
LILIOPSIDA	ORCHIDACEAE	*Brachionidium dressleri*	EN	II
LILIOPSIDA	ORCHIDACEAE	*Brachionidium pteroglossum*	CR	II
LILIOPSIDA	ORCHIDACEAE	*Bulbophyllum anjozorobeense*	EN	II
LILIOPSIDA	ORCHIDACEAE	*Bulbophyllum auriflorum*	EN	II
LILIOPSIDA	ORCHIDACEAE	*Bulbophyllum evrardii*	EN	II
LILIOPSIDA	ORCHIDACEAE	*Bulbophyllum hamelinii*	VU	II
LILIOPSIDA	ORCHIDACEAE	*Bulbophyllum hirsutiusculum*	CR	II
LILIOPSIDA	ORCHIDACEAE	*Bulbophyllum jumelleanum*	EN	II
LILIOPSIDA	ORCHIDACEAE	*Bulbophyllum kainochiloides*	EN	II
LILIOPSIDA	ORCHIDACEAE	*Bulbophyllum moratii*	CR	II
LILIOPSIDA	ORCHIDACEAE	*Bulbophyllum paleiferum*	EN	II
LILIOPSIDA	ORCHIDACEAE	*Bulbophyllum sanguineum*	CR	II
LILIOPSIDA	ORCHIDACEAE	*Bulbophyllum tampoketsense*	CR	II
LILIOPSIDA	ORCHIDACEAE	*Ceratocentron fesselii*	CR	II
LILIOPSIDA	ORCHIDACEAE	*Cryptopus brachiatus*	EN	II
LILIOPSIDA	ORCHIDACEAE	*Cryptopus dissectus*	CR	II
LILIOPSIDA	ORCHIDACEAE	*Cymbidiella pardalina*	EN	II
LILIOPSIDA	ORCHIDACEAE	*Cynorkis aphylla*	EN	II
LILIOPSIDA	ORCHIDACEAE	*Cynorkis bimaculata*	CR	II
LILIOPSIDA	ORCHIDACEAE	*Cynorkis monadenia*	CR	II

Class	Family	Species	Red List category	CITES Appendix
LILIOPSIDA	ORCHIDACEAE	*Cynorkis perrieri*	EN	II
LILIOPSIDA	ORCHIDACEAE	*Cynorkis sacculata*	CR	II
LILIOPSIDA	ORCHIDACEAE	*Cynorkis stolonifera*	EN	II
LILIOPSIDA	ORCHIDACEAE	*Cynorkis tenerrima*	EN	II
LILIOPSIDA	ORCHIDACEAE	*Cypripedium arietinum*	NT	II
LILIOPSIDA	ORCHIDACEAE	*Cypripedium bardolphianum*	VU	II
LILIOPSIDA	ORCHIDACEAE	*Cypripedium calcicola*	EN	II
LILIOPSIDA	ORCHIDACEAE	*Cypripedium californicum*	EN	II
LILIOPSIDA	ORCHIDACEAE	*Cypripedium candidum*	VU	II
LILIOPSIDA	ORCHIDACEAE	*Cypripedium cordigerum*	VU	II
LILIOPSIDA	ORCHIDACEAE	*Cypripedium daweishanense*	CR	II
LILIOPSIDA	ORCHIDACEAE	*Cypripedium debile*	VU	II
LILIOPSIDA	ORCHIDACEAE	*Cypripedium elegans*	EN	II
LILIOPSIDA	ORCHIDACEAE	*Cypripedium fargesii*	EN	II
LILIOPSIDA	ORCHIDACEAE	*Cypripedium farreri*	EN	II
LILIOPSIDA	ORCHIDACEAE	*Cypripedium fasciculatum*	VU	II
LILIOPSIDA	ORCHIDACEAE	*Cypripedium fasciolatum*	EN	II
LILIOPSIDA	ORCHIDACEAE	*Cypripedium flavum*	VU	II
LILIOPSIDA	ORCHIDACEAE	*Cypripedium formosanum*	EN	II
LILIOPSIDA	ORCHIDACEAE	*Cypripedium forrestii*	EN	II
LILIOPSIDA	ORCHIDACEAE	*Cypripedium franchetii*	EN	II
LILIOPSIDA	ORCHIDACEAE	*Cypripedium froschii*	CR	II
LILIOPSIDA	ORCHIDACEAE	*Cypripedium henryi*	VU	II
LILIOPSIDA	ORCHIDACEAE	*Cypripedium himalaicum*	EN	II
LILIOPSIDA	ORCHIDACEAE	*Cypripedium japonicum*	EN	II
LILIOPSIDA	ORCHIDACEAE	*Cypripedium lentiginosum*	EN	II
LILIOPSIDA	ORCHIDACEAE	*Cypripedium lichiangense*	EN	II
LILIOPSIDA	ORCHIDACEAE	*Cypripedium ludlowii*	EN	II
LILIOPSIDA	ORCHIDACEAE	*Cypripedium malipoense*	CR	II
LILIOPSIDA	ORCHIDACEAE	*Cypripedium margaritaceum*	EN	II
LILIOPSIDA	ORCHIDACEAE	*Cypripedium micranthum*	EN	II
LILIOPSIDA	ORCHIDACEAE	*Cypripedium montanum*	VU	II
LILIOPSIDA	ORCHIDACEAE	*Cypripedium palangshanense*	VU	II
LILIOPSIDA	ORCHIDACEAE	*Cypripedium passerinum*	VU	II
LILIOPSIDA	ORCHIDACEAE	*Cypripedium plectrochilum*	VU	II
LILIOPSIDA	ORCHIDACEAE	*Cypripedium segawae*	CR	II
LILIOPSIDA	ORCHIDACEAE	*Cypripedium shanxiense*	NT	II
LILIOPSIDA	ORCHIDACEAE	*Cypripedium sichuanense*	EN	II
LILIOPSIDA	ORCHIDACEAE	*Cypripedium singchii*	EN	II
LILIOPSIDA	ORCHIDACEAE	*Cypripedium subtropicum*	EN	II
LILIOPSIDA	ORCHIDACEAE	*Cypripedium taibaiense*	CR	II
LILIOPSIDA	ORCHIDACEAE	*Cypripedium wardii*	EN	II
LILIOPSIDA	ORCHIDACEAE	*Cypripedium wumengense*	CR	II
LILIOPSIDA	ORCHIDACEAE	*Cypripedium yatabeanum*	NT	II
LILIOPSIDA	ORCHIDACEAE	*Cypripedium yunnanense*	EN	II

**Class**	**Family**	**Species**	**Red List category**	**CITES Appendix**
LILIOPSIDA	ORCHIDACEAE	*Dactylorhiza elata*	NT	II
LILIOPSIDA	ORCHIDACEAE	*Dactylorhiza euxina*	NT	II
LILIOPSIDA	ORCHIDACEAE	*Dendrobium atroviolaceum*	VU	II
LILIOPSIDA	ORCHIDACEAE	*Dendrobium mussauense*	VU	II
LILIOPSIDA	ORCHIDACEAE	*Dendrobium schuetzei*	CR	II
LILIOPSIDA	ORCHIDACEAE	*Dendrobium tangerinum*	VU	II
LILIOPSIDA	ORCHIDACEAE	*Dendrobium williamsianum*	NT	II
LILIOPSIDA	ORCHIDACEAE	*Disperis bosseri*	CR	II
LILIOPSIDA	ORCHIDACEAE	*Disperis erucifera*	EN	II
LILIOPSIDA	ORCHIDACEAE	*Disperis lanceolata*	EN	II
LILIOPSIDA	ORCHIDACEAE	*Encyclia kingsii*	CR	II
LILIOPSIDA	ORCHIDACEAE	*Eulophia nervosa*	CR	II
LILIOPSIDA	ORCHIDACEAE	*Eulophiella galbana*	VU	II
LILIOPSIDA	ORCHIDACEAE	*Galeandra bicarinata*	EN	II
LILIOPSIDA	ORCHIDACEAE	*Gastrorchis tuberculosa*	EN	II
LILIOPSIDA	ORCHIDACEAE	*Gomesa silvana*	EN	II
LILIOPSIDA	ORCHIDACEAE	*Lankesterella gnomus*	NT	II
LILIOPSIDA	ORCHIDACEAE	*Masdevallia apparitio*	CR	II
LILIOPSIDA	ORCHIDACEAE	*Myrmecophila thomsoniana*	EN	II
LILIOPSIDA	ORCHIDACEAE	*Paphiopedilum acmodontum*	EN	I
LILIOPSIDA	ORCHIDACEAE	*Paphiopedilum adductum*	CR	I
LILIOPSIDA	ORCHIDACEAE	*Paphiopedilum appletonianum*	EN	I
LILIOPSIDA	ORCHIDACEAE	*Paphiopedilum areeanum*	EN	I
LILIOPSIDA	ORCHIDACEAE	*Paphiopedilum argus*	EN	I
LILIOPSIDA	ORCHIDACEAE	*Paphiopedilum armeniacum*	EN	I
LILIOPSIDA	ORCHIDACEAE	*Paphiopedilum barbatum*	EN	I
LILIOPSIDA	ORCHIDACEAE	*Paphiopedilum barbigerum*	EN	I
LILIOPSIDA	ORCHIDACEAE	*Paphiopedilum bellatulum*	EN	I
LILIOPSIDA	ORCHIDACEAE	*Paphiopedilum bougainvilleanum*	CR	I
LILIOPSIDA	ORCHIDACEAE	*Paphiopedilum bullenianum*	EN	I
LILIOPSIDA	ORCHIDACEAE	*Paphiopedilum callosum*	EN	I
LILIOPSIDA	ORCHIDACEAE	*Paphiopedilum canhii*	CR	I
LILIOPSIDA	ORCHIDACEAE	*Paphiopedilum charlesworthii*	EN	I
LILIOPSIDA	ORCHIDACEAE	*Paphiopedilum ciliolare*	EN	I
LILIOPSIDA	ORCHIDACEAE	*Paphiopedilum concolor*	EN	I
LILIOPSIDA	ORCHIDACEAE	*Paphiopedilum cornuatum*	CR	I
LILIOPSIDA	ORCHIDACEAE	*Paphiopedilum dayanum*	CR	I
LILIOPSIDA	ORCHIDACEAE	*Paphiopedilum delenatii*	CR	I
LILIOPSIDA	ORCHIDACEAE	*Paphiopedilum dianthum*	EN	I
LILIOPSIDA	ORCHIDACEAE	*Paphiopedilum druryi*	CR	I
LILIOPSIDA	ORCHIDACEAE	*Paphiopedilum emersonii*	CR	I
LILIOPSIDA	ORCHIDACEAE	*Paphiopedilum exul*	CR	I
LILIOPSIDA	ORCHIDACEAE	*Paphiopedilum fairrieanum*	CR	I
LILIOPSIDA	ORCHIDACEAE	*Paphiopedilum fowliei*	CR	I

**Class**	**Family**	**Species**	**Red List category**	**CITES Appendix**
LILIOPSIDA	ORCHIDACEAE	*Paphiopedilum gigantifolium*	CR	I
LILIOPSIDA	ORCHIDACEAE	*Paphiopedilum glanduliferum*	EN	I
LILIOPSIDA	ORCHIDACEAE	*Paphiopedilum glaucophyllum*	EN	I
LILIOPSIDA	ORCHIDACEAE	*Paphiopedilum godefroyae*	EN	I
LILIOPSIDA	ORCHIDACEAE	*Paphiopedilum gratrixianum*	CR	I
LILIOPSIDA	ORCHIDACEAE	*Paphiopedilum guangdongense*	CR	I
LILIOPSIDA	ORCHIDACEAE	*Paphiopedilum hangianum*	CR	I
LILIOPSIDA	ORCHIDACEAE	*Paphiopedilum haynaldianum*	EN	I
LILIOPSIDA	ORCHIDACEAE	*Paphiopedilum helenae*	CR	I
LILIOPSIDA	ORCHIDACEAE	*Paphiopedilum hennisianum*	EN	I
LILIOPSIDA	ORCHIDACEAE	*Paphiopedilum henryanum*	CR	I
LILIOPSIDA	ORCHIDACEAE	*Paphiopedilum hirsutissimum*	VU	I
LILIOPSIDA	ORCHIDACEAE	*Paphiopedilum hookerae*	EN	I
LILIOPSIDA	ORCHIDACEAE	*Paphiopedilum inamorii*	CR	I
LILIOPSIDA	ORCHIDACEAE	*Paphiopedilum insigne*	EN	I
LILIOPSIDA	ORCHIDACEAE	*Paphiopedilum intaniae*	CR	I
LILIOPSIDA	ORCHIDACEAE	*Paphiopedilum javanicum*	EN	I
LILIOPSIDA	ORCHIDACEAE	*Paphiopedilum kolopakingii*	CR	I
LILIOPSIDA	ORCHIDACEAE	*Paphiopedilum lawrenceanum*	CR	I
LILIOPSIDA	ORCHIDACEAE	*Paphiopedilum liemianum*	CR	I
LILIOPSIDA	ORCHIDACEAE	*Paphiopedilum lowii*	EN	I
LILIOPSIDA	ORCHIDACEAE	*Paphiopedilum malipoense*	EN	I
LILIOPSIDA	ORCHIDACEAE	*Paphiopedilum micranthum*	CR	I
LILIOPSIDA	ORCHIDACEAE	*Paphiopedilum niveum*	EN	I
LILIOPSIDA	ORCHIDACEAE	*Paphiopedilum ooii*	CR	I
LILIOPSIDA	ORCHIDACEAE	*Paphiopedilum papuanum*	EN	I
LILIOPSIDA	ORCHIDACEAE	*Paphiopedilum parishii*	EN	I
LILIOPSIDA	ORCHIDACEAE	*Paphiopedilum parnatanum*	CR	I
LILIOPSIDA	ORCHIDACEAE	*Paphiopedilum philippinense*	NT	I
LILIOPSIDA	ORCHIDACEAE	*Paphiopedilum platyphyllum*	CR	I
LILIOPSIDA	ORCHIDACEAE	*Paphiopedilum primulinum*	CR	I
LILIOPSIDA	ORCHIDACEAE	*Paphiopedilum purpuratum*	CR	I
LILIOPSIDA	ORCHIDACEAE	*Paphiopedilum qingyongii*	CR	I
LILIOPSIDA	ORCHIDACEAE	*Paphiopedilum randsii*	EN	I
LILIOPSIDA	ORCHIDACEAE	*Paphiopedilum rothschildianum*	CR	I
LILIOPSIDA	ORCHIDACEAE	*Paphiopedilum sanderianum*	CR	I
LILIOPSIDA	ORCHIDACEAE	*Paphiopedilum sangii*	CR	I
LILIOPSIDA	ORCHIDACEAE	*Paphiopedilum schoseri*	CR	I
LILIOPSIDA	ORCHIDACEAE	*Paphiopedilum spicerianum*	EN	I
LILIOPSIDA	ORCHIDACEAE	*Paphiopedilum stonei*	CR	I
LILIOPSIDA	ORCHIDACEAE	*Paphiopedilum sugiyamanum*	CR	I
LILIOPSIDA	ORCHIDACEAE	*Paphiopedilum sukhakulii*	CR	I
LILIOPSIDA	ORCHIDACEAE	*Paphiopedilum supardii*	CR	I

**Class**	**Family**	**Species**	**Red List category**	**CITES Appendix**
LILIOPSIDA	ORCHIDACEAE	*Paphiopedilum superbiens*	EN	I
LILIOPSIDA	ORCHIDACEAE	*Paphiopedilum thaianum*	CR	I
LILIOPSIDA	ORCHIDACEAE	*Paphiopedilum tigrinum*	EN	I
LILIOPSIDA	ORCHIDACEAE	*Paphiopedilum tonsum*	EN	I
LILIOPSIDA	ORCHIDACEAE	*Paphiopedilum tranlienianum*	CR	I
LILIOPSIDA	ORCHIDACEAE	*Paphiopedilum urbanianum*	CR	I
LILIOPSIDA	ORCHIDACEAE	*Paphiopedilum venustum*	EN	I
LILIOPSIDA	ORCHIDACEAE	*Paphiopedilum victoria-mariae*	CR	I
LILIOPSIDA	ORCHIDACEAE	*Paphiopedilum victoria-regina*	CR	I
LILIOPSIDA	ORCHIDACEAE	*Paphiopedilum vietnamense*	CR	I
LILIOPSIDA	ORCHIDACEAE	*Paphiopedilum villosum*	VU	I
LILIOPSIDA	ORCHIDACEAE	*Paphiopedilum violascens*	EN	I
LILIOPSIDA	ORCHIDACEAE	*Paphiopedilum wardii*	EN	I
LILIOPSIDA	ORCHIDACEAE	*Paphiopedilum wenshanense*	CR	I
LILIOPSIDA	ORCHIDACEAE	*Paphiopedilum wentworthianum*	CR	I
LILIOPSIDA	ORCHIDACEAE	*Paphiopedilum wilhelminae*	EN	I
LILIOPSIDA	ORCHIDACEAE	*Phalaenopsis lindenii*	EN	II
LILIOPSIDA	ORCHIDACEAE	*Phalaenopsis micholitzii*	CR	II
LILIOPSIDA	ORCHIDACEAE	*Phalaenopsis violacea*	VU	II
LILIOPSIDA	ORCHIDACEAE	*Pholidota chinensis*	NT	II
LILIOPSIDA	ORCHIDACEAE	*Phragmipedium andreettae*	CR	I
LILIOPSIDA	ORCHIDACEAE	*Phragmipedium caudatum*	EN	I
LILIOPSIDA	ORCHIDACEAE	*Phragmipedium christiansenianum*	CR	I
LILIOPSIDA	ORCHIDACEAE	*Phragmipedium exstaminodium*	CR	I
LILIOPSIDA	ORCHIDACEAE	*Phragmipedium fischeri*	CR	I
LILIOPSIDA	ORCHIDACEAE	*Phragmipedium hartwegii*	EN	I
LILIOPSIDA	ORCHIDACEAE	*Phragmipedium hirtzii*	EN	I
LILIOPSIDA	ORCHIDACEAE	*Phragmipedium klotzschianum*	EN	I
LILIOPSIDA	ORCHIDACEAE	*Phragmipedium kovachii*	CR	I
LILIOPSIDA	ORCHIDACEAE	*Phragmipedium manzurii*	CR	I
LILIOPSIDA	ORCHIDACEAE	*Phragmipedium reticulatum*	VU	I
LILIOPSIDA	ORCHIDACEAE	*Phragmipedium richteri*	CR	I
LILIOPSIDA	ORCHIDACEAE	*Phragmipedium schlimii*	EN	I
LILIOPSIDA	ORCHIDACEAE	*Phragmipedium tetzlaffianum*	CR	I
LILIOPSIDA	ORCHIDACEAE	*Phragmipedium warszewiczianum*	VU	I
LILIOPSIDA	ORCHIDACEAE	*Platanthera algeriensis*	NT	II
LILIOPSIDA	ORCHIDACEAE	*Telipogon biolleyi*	NT	II
LILIOPSIDA	ORCHIDACEAE	*Vanda javierae*	EN	II
LILIOPSIDA	ORCHIDACEAE	*Vanda scandens*	EN	II
LILIOPSIDA	ORCHIDACEAE	*Vanda spathulata*	VU	II
LILIOPSIDA	ORCHIDACEAE	*Vanilla cribbiana*	CR	II
LILIOPSIDA	ORCHIDACEAE	*Vanilla hartii*	EN	II

Class	Family	Species	Red List category	CITES Appendix
LILIOPSIDA	ORCHIDACEAE	*Vanilla planifolia*	EN	II
LILIOPSIDA	DIOSCOREACEAE	*Dioscorea sylvatica*	VU	NC
LILIOPSIDA	LILIACEAE	*Fritillaria davisii*	NT	NC
LILIOPSIDA	BROMELIACEAE	*Aechmea manzanaresiana*	EN	NC
LILIOPSIDA	BROMELIACEAE	*Cryptanthus diamantinensis*	CR	NC
LILIOPSIDA	BROMELIACEAE	*Cryptanthus pseudoscaposus*	EN	NC
LILIOPSIDA	BROMELIACEAE	*Guzmania bismarckii*	CR	NC
LILIOPSIDA	BROMELIACEAE	*Tillandsia candida*	VU	NC
LILIOPSIDA	BROMELIACEAE	*Tillandsia paniculata*	NT	NC
LILIOPSIDA	BROMELIACEAE	*Tillandsia yerba-santae*	CR	NC
LILIOPSIDA	POACEAE	*Cymbopogon bhutanicus*	VU	NC
LILIOPSIDA	MUSACEAE	*Musa coccinea*	EN	NC
LILIOPSIDA	ZINGIBERACEAE	*Curcuma alismatifolia*	NT	NC
LILIOPSIDA	ZINGIBERACEAE	*Curcuma caulina*	EN	NC
LILIOPSIDA	ZINGIBERACEAE	*Curcuma coriacea*	EN	NC
LILIOPSIDA	ZINGIBERACEAE	*Curcuma pseudomontana*	VU	NC
LILIOPSIDA	ZINGIBERACEAE	*Curcuma rhabdota*	VU	NC
LILIOPSIDA	ZINGIBERACEAE	*Curcuma sparganiifolia*	NT	NC
LILIOPSIDA	ZINGIBERACEAE	*Curcuma supraneeana*	CR	NC
LILIOPSIDA	ZINGIBERACEAE	*Etlingera venusta*	VU	NC
LILIOPSIDA	ZINGIBERACEAE	*Hedychium glabrum*	NT	NC
LILIOPSIDA	ZINGIBERACEAE	*Sulettaria lambirensis*	VU	NC
LILIOPSIDA	ZINGIBERACEAE	*Zingiber niveum*	EN	NC
MAGNOLIOPSIDA	ASTERACEAE	*Anacyclus pyrethrum*	VU	NC
MAGNOLIOPSIDA	ASTERACEAE	*Saussurea costus*	CR	I
MAGNOLIOPSIDA	SCHISANDRACEAE	*Illicium griffithii*	EN	NC
MAGNOLIOPSIDA	AIZOACEAE	*Lithops francisci*	VU	NC
MAGNOLIOPSIDA	ANACAMPSEROTACEAE	*Anacampseros decapitata*	VU	II
MAGNOLIOPSIDA	CACTACEAE	*Acharagma aguirreanum*	CR	II
MAGNOLIOPSIDA	CACTACEAE	*Ariocarpus agavoides*	EN	I
MAGNOLIOPSIDA	CACTACEAE	*Ariocarpus bravoanus*	EN	I
MAGNOLIOPSIDA	CACTACEAE	*Ariocarpus kotschoubeyanus*	NT	I
MAGNOLIOPSIDA	CACTACEAE	*Ariocarpus scaphirostris*	EN	I
MAGNOLIOPSIDA	CACTACEAE	*Astrophytum asterias*	VU	I
MAGNOLIOPSIDA	CACTACEAE	*Astrophytum caput-medusae*	CR	II
MAGNOLIOPSIDA	CACTACEAE	*Astrophytum coahuilense*	VU	II
MAGNOLIOPSIDA	CACTACEAE	*Astrophytum ornatum*	VU	II
MAGNOLIOPSIDA	CACTACEAE	*Aztekium hintonii*	NT	II
MAGNOLIOPSIDA	CACTACEAE	*Cephalocereus senilis*	EN	II
MAGNOLIOPSIDA	CACTACEAE	*Coleocephalocereus purpureus*	CR	II
MAGNOLIOPSIDA	CACTACEAE	*Copiapoa ahremephiana*	CR	II
MAGNOLIOPSIDA	CACTACEAE	*Copiapoa angustiflora*	CR	II
MAGNOLIOPSIDA	CACTACEAE	*Copiapoa decorticans*	CR	II
MAGNOLIOPSIDA	CACTACEAE	*Copiapoa esmeraldana*	CR	II

Class	Family	Species	Red List category	CITES Appendix
MAGNOLIOPSIDA	CACTACEAE	*Copiapoa grandiflora*	EN	II
MAGNOLIOPSIDA	CACTACEAE	*Copiapoa humilis*	NT	II
MAGNOLIOPSIDA	CACTACEAE	*Copiapoa hypogaea*	EN	II
MAGNOLIOPSIDA	CACTACEAE	*Copiapoa marginata*	NT	II
MAGNOLIOPSIDA	CACTACEAE	*Copiapoa serpentisulcata*	EN	II
MAGNOLIOPSIDA	CACTACEAE	*Coryphantha maiz-tablasensis*	EN	II
MAGNOLIOPSIDA	CACTACEAE	*Discocactus diersianus*	EN	I
MAGNOLIOPSIDA	CACTACEAE	*Discocactus petr-halfari*	CR	I
MAGNOLIOPSIDA	CACTACEAE	*Echinocactus grusonii*	EN	II
MAGNOLIOPSIDA	CACTACEAE	*Echinocereus nivosus*	CR	II
MAGNOLIOPSIDA	CACTACEAE	*Echinocereus schmollii*	EN	I
MAGNOLIOPSIDA	CACTACEAE	*Echinopsis backebergii*	VU	II
MAGNOLIOPSIDA	CACTACEAE	*Echinopsis chrysantha*	VU	II
MAGNOLIOPSIDA	CACTACEAE	*Echinopsis famatinensis*	VU	II
MAGNOLIOPSIDA	CACTACEAE	*Echinopsis hertrichiana*	EN	II
MAGNOLIOPSIDA	CACTACEAE	*Echinopsis pampana*	EN	II
MAGNOLIOPSIDA	CACTACEAE	*Eriosyce aspillagae*	EN	II
MAGNOLIOPSIDA	CACTACEAE	*Eriosyce calderana*	EN	II
MAGNOLIOPSIDA	CACTACEAE	*Eriosyce confinis*	VU	II
MAGNOLIOPSIDA	CACTACEAE	*Eriosyce crispa*	EN	II
MAGNOLIOPSIDA	CACTACEAE	*Eriosyce esmeraldana*	EN	II
MAGNOLIOPSIDA	CACTACEAE	*Eriosyce iquiquensis*	EN	II
MAGNOLIOPSIDA	CACTACEAE	*Eriosyce laui*	CR	II
MAGNOLIOPSIDA	CACTACEAE	*Eriosyce napina*	VU	II
MAGNOLIOPSIDA	CACTACEAE	*Eriosyce occulta*	EN	II
MAGNOLIOPSIDA	CACTACEAE	*Eriosyce odieri*	VU	II
MAGNOLIOPSIDA	CACTACEAE	*Eriosyce recondita*	VU	II
MAGNOLIOPSIDA	CACTACEAE	*Eriosyce rodentiophila*	VU	II
MAGNOLIOPSIDA	CACTACEAE	*Eriosyce senilis*	VU	II
MAGNOLIOPSIDA	CACTACEAE	*Eriosyce simulans*	EN	II
MAGNOLIOPSIDA	CACTACEAE	*Eriosyce sociabilis*	EN	II
MAGNOLIOPSIDA	CACTACEAE	*Eriosyce taltalensis*	VU	II
MAGNOLIOPSIDA	CACTACEAE	*Geohintonia mexicana*	NT	II
MAGNOLIOPSIDA	CACTACEAE	*Gymnocalycium neuhuberi*	CR	II
MAGNOLIOPSIDA	CACTACEAE	*Gymnocalycium ragonesei*	CR	II
MAGNOLIOPSIDA	CACTACEAE	*Hatiora gaertneri*	VU	II
MAGNOLIOPSIDA	CACTACEAE	*Hatiora herminiae*	EN	II
MAGNOLIOPSIDA	CACTACEAE	*Hatiora rosea*	NT	II
MAGNOLIOPSIDA	CACTACEAE	*Lophophora diffusa*	VU	II
MAGNOLIOPSIDA	CACTACEAE	*Lophophora williamsii*	VU	II
MAGNOLIOPSIDA	CACTACEAE	*Maihueniopsis minuta*	EN	II
MAGNOLIOPSIDA	CACTACEAE	*Mammillaria albicoma*	EN	II
MAGNOLIOPSIDA	CACTACEAE	*Mammillaria albiflora*	CR	II
MAGNOLIOPSIDA	CACTACEAE	*Mammillaria anniana*	CR	II
MAGNOLIOPSIDA	CACTACEAE	*Mammillaria armillata*	VU	II

Class	Family	Species	Red List category	CITES Appendix
MAGNOLIOPSIDA	CACTACEAE	*Mammillaria aureilanata*	EN	II
MAGNOLIOPSIDA	CACTACEAE	*Mammillaria blossfeldiana*	NT	II
MAGNOLIOPSIDA	CACTACEAE	*Mammillaria bombycina*	VU	II
MAGNOLIOPSIDA	CACTACEAE	*Mammillaria carmenae*	CR	II
MAGNOLIOPSIDA	CACTACEAE	*Mammillaria deherdtiana*	VU	II
MAGNOLIOPSIDA	CACTACEAE	*Mammillaria gasseriana*	EN	II
MAGNOLIOPSIDA	CACTACEAE	*Mammillaria glochidiata*	CR	II
MAGNOLIOPSIDA	CACTACEAE	*Mammillaria herrerae*	CR	II
MAGNOLIOPSIDA	CACTACEAE	*Mammillaria humboldtii*	CR	II
MAGNOLIOPSIDA	CACTACEAE	*Mammillaria laui*	CR	II
MAGNOLIOPSIDA	CACTACEAE	*Mammillaria marcosii*	CR	II
MAGNOLIOPSIDA	CACTACEAE	*Mammillaria mathildae*	EN	II
MAGNOLIOPSIDA	CACTACEAE	*Mammillaria melaleuca*	EN	II
MAGNOLIOPSIDA	CACTACEAE	*Mammillaria napina*	NT	II
MAGNOLIOPSIDA	CACTACEAE	*Mammillaria parkinsonii*	EN	II
MAGNOLIOPSIDA	CACTACEAE	*Mammillaria pectinifera*	EN	I
MAGNOLIOPSIDA	CACTACEAE	*Mammillaria pennispinosa*	CR	II
MAGNOLIOPSIDA	CACTACEAE	*Mammillaria perbella*	VU	II
MAGNOLIOPSIDA	CACTACEAE	*Mammillaria plumosa*	NT	II
MAGNOLIOPSIDA	CACTACEAE	*Mammillaria rettigiana*	EN	II
MAGNOLIOPSIDA	CACTACEAE	*Mammillaria sanchez-mejoradae*	CR	II
MAGNOLIOPSIDA	CACTACEAE	*Mammillaria schiedeana*	VU	II
MAGNOLIOPSIDA	CACTACEAE	*Mammillaria surculosa*	EN	II
MAGNOLIOPSIDA	CACTACEAE	*Mammillaria theresae*	CR	II
MAGNOLIOPSIDA	CACTACEAE	*Mammillaria zeilmanniana*	CR	II
MAGNOLIOPSIDA	CACTACEAE	*Mammillaria zublerae*	EN	II
MAGNOLIOPSIDA	CACTACEAE	*Matucana krahnii*	VU	II
MAGNOLIOPSIDA	CACTACEAE	*Matucana madisoniorum*	CR	II
MAGNOLIOPSIDA	CACTACEAE	*Matucana paucicostata*	VU	II
MAGNOLIOPSIDA	CACTACEAE	*Matucana pujupatii*	NT	II
MAGNOLIOPSIDA	CACTACEAE	*Matucana ritteri*	CR	II
MAGNOLIOPSIDA	CACTACEAE	*Matucana tuberculata*	EN	II
MAGNOLIOPSIDA	CACTACEAE	*Matucana weberbaueri*	EN	II
MAGNOLIOPSIDA	CACTACEAE	*Melocactus ferreophilus*	CR	II
MAGNOLIOPSIDA	CACTACEAE	*Mila caespitosa*	VU	II
MAGNOLIOPSIDA	CACTACEAE	*Obregonia denegrii*	EN	I
MAGNOLIOPSIDA	CACTACEAE	*Oroya peruviana*	EN	II
MAGNOLIOPSIDA	CACTACEAE	*Parodia buiningii*	CR	II
MAGNOLIOPSIDA	CACTACEAE	*Parodia mueller-melchersii*	EN	II
MAGNOLIOPSIDA	CACTACEAE	*Parodia scopa*	VU	II
MAGNOLIOPSIDA	CACTACEAE	*Parodia werdermanniana*	CR	II
MAGNOLIOPSIDA	CACTACEAE	*Pediocactus bradyi*	NT	I
MAGNOLIOPSIDA	CACTACEAE	*Pediocactus despainii*	NT	I
MAGNOLIOPSIDA	CACTACEAE	*Pediocactus knowltonii*	CR	I

Class	Family	Species	Red List category	CITES Appendix
MAGNOLIOPSIDA	CACTACEAE	*Pediocactus paradinei*	EN	I
MAGNOLIOPSIDA	CACTACEAE	*Pierrebraunia bahiensis*	VU	II
MAGNOLIOPSIDA	CACTACEAE	*Pygmaeocereus bieblii*	EN	II
MAGNOLIOPSIDA	CACTACEAE	*Rebutia albipectinata*	EN	II
MAGNOLIOPSIDA	CACTACEAE	*Schlumbergera opuntioides*	VU	II
MAGNOLIOPSIDA	CACTACEAE	*Schlumbergera orssichiana*	EN	II
MAGNOLIOPSIDA	CACTACEAE	*Schlumbergera russelliana*	EN	II
MAGNOLIOPSIDA	CACTACEAE	*Sclerocactus brevispinus*	CR	I
MAGNOLIOPSIDA	CACTACEAE	*Sclerocactus sileri*	VU	I
MAGNOLIOPSIDA	CACTACEAE	*Sclerocactus wrightiae*	NT	I
MAGNOLIOPSIDA	CACTACEAE	*Tephrocactus bonnieae*	EN	II
MAGNOLIOPSIDA	CACTACEAE	*Turbinicarpus gielsdorfianus*	CR	I
MAGNOLIOPSIDA	CACTACEAE	*Turbinicarpus hoferi*	CR	I
MAGNOLIOPSIDA	CACTACEAE	*Turbinicarpus horripilus*	EN	I
MAGNOLIOPSIDA	CACTACEAE	*Turbinicarpus laui*	CR	I
MAGNOLIOPSIDA	CACTACEAE	*Turbinicarpus lophophoroides*	NT	I
MAGNOLIOPSIDA	CACTACEAE	*Turbinicarpus pseudomacrochele*	EN	I
MAGNOLIOPSIDA	CACTACEAE	*Turbinicarpus saueri*	VU	I
MAGNOLIOPSIDA	CACTACEAE	*Turbinicarpus schmiedickeanus*	NT	I
MAGNOLIOPSIDA	CACTACEAE	*Turbinicarpus subterraneus*	EN	I
MAGNOLIOPSIDA	CACTACEAE	*Turbinicarpus swobodae*	CR	I
MAGNOLIOPSIDA	CACTACEAE	*Turbinicarpus valdezianus*	VU	I
MAGNOLIOPSIDA	CACTACEAE	*Uebelmannia buiningii*	CR	I
MAGNOLIOPSIDA	CACTACEAE	*Uebelmannia pectinifera*	EN	I
MAGNOLIOPSIDA	CARYOPHYLLACEAE	*Silene chustupica*	CR	NC
MAGNOLIOPSIDA	DILLENIACEAE	*Dillenia crenatifolia*	VU	NC
MAGNOLIOPSIDA	DILLENIACEAE	*Dillenia philippinensis*	NT	NC
MAGNOLIOPSIDA	CAPRIFOLIACEAE	*Nardostachys jatamansi*	CR	II
MAGNOLIOPSIDA	BALSAMINACEAE	*Impatiens adenioides*	CR	NC
MAGNOLIOPSIDA	BALSAMINACEAE	*Impatiens mirabilis*	NT	NC
MAGNOLIOPSIDA	BALSAMINACEAE	*Impatiens parishii*	NT	NC
MAGNOLIOPSIDA	EBENACEAE	*Diospyros crassiflora*	VU	II
MAGNOLIOPSIDA	EBENACEAE	*Diospyros insularis*	EN	NC
MAGNOLIOPSIDA	SAPOTACEAE	*Baillonella toxisperma*	VU	NC
MAGNOLIOPSIDA	SAPOTACEAE	*Burckella erythrophylla*	EN	NC
MAGNOLIOPSIDA	SAPOTACEAE	*Burckella macropoda*	NT	NC
MAGNOLIOPSIDA	SAPOTACEAE	*Madhuca crassipes*	NT	NC
MAGNOLIOPSIDA	SAPOTACEAE	*Madhuca curtisii*	VU	NC
MAGNOLIOPSIDA	SAPOTACEAE	*Madhuca endertii*	VU	NC
MAGNOLIOPSIDA	SAPOTACEAE	*Madhuca engleri*	EN	NC
MAGNOLIOPSIDA	SAPOTACEAE	*Madhuca erythrophylla*	EN	NC
MAGNOLIOPSIDA	SAPOTACEAE	*Madhuca kingiana*	NT	NC
MAGNOLIOPSIDA	SAPOTACEAE	*Madhuca korthalsii*	NT	NC

Class	Family	Species	Red List category	CITES Appendix
MAGNOLIOPSIDA	SAPOTACEAE	*Madhuca motleyana*	NT	NC
MAGNOLIOPSIDA	SAPOTACEAE	*Madhuca sericea*	VU	NC
MAGNOLIOPSIDA	SAPOTACEAE	*Madhuca spectabilis*	EN	NC
MAGNOLIOPSIDA	SAPOTACEAE	*Madhuca utilis*	EN	NC
MAGNOLIOPSIDA	SAPOTACEAE	*Planchonella maingayi*	NT	NC
MAGNOLIOPSIDA	FABACEAE	*Afzelia africana*	VU	NC
MAGNOLIOPSIDA	FABACEAE	*Brachystegia leonensis*	VU	NC
MAGNOLIOPSIDA	FABACEAE	*Cynometra malaccensis*	NT	NC
MAGNOLIOPSIDA	FABACEAE	*Dalbergia abrahamii*	EN	II
MAGNOLIOPSIDA	FABACEAE	*Dalbergia andapensis*	EN	II
MAGNOLIOPSIDA	FABACEAE	*Dalbergia calderonii*	CR	II
MAGNOLIOPSIDA	FABACEAE	*Dalbergia chapelieri*	NT	II
MAGNOLIOPSIDA	FABACEAE	*Dalbergia chlorocarpa*	VU	II
MAGNOLIOPSIDA	FABACEAE	*Dalbergia congestiflora*	EN	II
MAGNOLIOPSIDA	FABACEAE	*Dalbergia davidii*	CR	II
MAGNOLIOPSIDA	FABACEAE	*Dalbergia delphinensis*	EN	II
MAGNOLIOPSIDA	FABACEAE	*Dalbergia glaberrima*	VU	II
MAGNOLIOPSIDA	FABACEAE	*Dalbergia glaucocarpa*	VU	II
MAGNOLIOPSIDA	FABACEAE	*Dalbergia glomerata*	CR	II
MAGNOLIOPSIDA	FABACEAE	*Dalbergia granadillo*	CR	II
MAGNOLIOPSIDA	FABACEAE	*Dalbergia greveana*	VU	II
MAGNOLIOPSIDA	FABACEAE	*Dalbergia hildebrandtii*	VU	II
MAGNOLIOPSIDA	FABACEAE	*Dalbergia humbertii*	VU	II
MAGNOLIOPSIDA	FABACEAE	*Dalbergia lemurica*	VU	II
MAGNOLIOPSIDA	FABACEAE	*Dalbergia madagascariensis*	VU	II
MAGNOLIOPSIDA	FABACEAE	*Dalbergia maritima*	EN	II
MAGNOLIOPSIDA	FABACEAE	*Dalbergia melanocardium*	EN	II
MAGNOLIOPSIDA	FABACEAE	*Dalbergia mollis*	VU	II
MAGNOLIOPSIDA	FABACEAE	*Dalbergia monticola*	VU	II
MAGNOLIOPSIDA	FABACEAE	*Dalbergia neoperrieri*	VU	II
MAGNOLIOPSIDA	FABACEAE	*Dalbergia palo-escrito*	EN	II
MAGNOLIOPSIDA	FABACEAE	*Dalbergia pervillei*	VU	II
MAGNOLIOPSIDA	FABACEAE	*Dalbergia pseudobaronii*	VU	II
MAGNOLIOPSIDA	FABACEAE	*Dalbergia purpurascens*	VU	II
MAGNOLIOPSIDA	FABACEAE	*Dalbergia retusa*	CR	II
MAGNOLIOPSIDA	FABACEAE	*Dalbergia suaresensis*	EN	II
MAGNOLIOPSIDA	FABACEAE	*Dalbergia tricolor*	VU	II
MAGNOLIOPSIDA	FABACEAE	*Dalbergia tsiandalana*	EN	II
MAGNOLIOPSIDA	FABACEAE	*Dalbergia urschii*	EN	II
MAGNOLIOPSIDA	FABACEAE	*Delonix pumila*	EN	NC
MAGNOLIOPSIDA	FABACEAE	*Gilbertiodendron limba*	NT	NC
MAGNOLIOPSIDA	FABACEAE	*Kalappia celebica*	VU	NC
MAGNOLIOPSIDA	FABACEAE	*Lathyrus hirticarpus*	EN	NC
MAGNOLIOPSIDA	FABACEAE	*Lathyrus libani*	EN	NC
MAGNOLIOPSIDA	FABACEAE	*Lathyrus odoratus*	CR	NC

**Class**	**Family**	**Species**	**Red List category**	**CITES Appendix**
MAGNOLIOPSIDA	FABACEAE	*Olneya tesota*	NT	NC
MAGNOLIOPSIDA	FABACEAE	*Paramachaerium gruberi*	CR	NC
MAGNOLIOPSIDA	FABACEAE	*Pericopsis mooniana*	VU	NC
MAGNOLIOPSIDA	FABACEAE	*Pterocarpus dalbergioides*	VU	NC
MAGNOLIOPSIDA	FABACEAE	*Pterocarpus erinaceus*	EN	II
MAGNOLIOPSIDA	FABACEAE	*Pterocarpus macrocarpus*	EN	NC
MAGNOLIOPSIDA	FABACEAE	*Pterocarpus marsupium*	NT	NC
MAGNOLIOPSIDA	FABACEAE	*Pterocarpus santalinus*	NT	II
MAGNOLIOPSIDA	FAGACEAE	*Castanopsis tungurrut*	EN	NC
MAGNOLIOPSIDA	FAGACEAE	*Lithocarpus vinkii*	VU	NC
MAGNOLIOPSIDA	JUGLANDACEAE	*Juglans neotropica*	EN	NC
MAGNOLIOPSIDA	NOTHOFAGACEAE	*Nothofagus alessandrii*	EN	NC
MAGNOLIOPSIDA	APOCYNACEAE	*Amsonia orientalis*	CR	NC
MAGNOLIOPSIDA	APOCYNACEAE	*Pachypodium baronii*	EN	I
MAGNOLIOPSIDA	APOCYNACEAE	*Pachypodium brevicaule*	VU	II
MAGNOLIOPSIDA	APOCYNACEAE	*Pachypodium eburneum*	CR	II
MAGNOLIOPSIDA	APOCYNACEAE	*Pachypodium inopinatum*	CR	II
MAGNOLIOPSIDA	APOCYNACEAE	*Pachypodium mikea*	EN	II
MAGNOLIOPSIDA	APOCYNACEAE	*Pachypodium namaquanum*	LR/nt	II
MAGNOLIOPSIDA	GENTIANACEAE	*Gentiana kurroo*	CR	NC
MAGNOLIOPSIDA	RUBIACEAE	*Balmea stormiae*	EN	I
MAGNOLIOPSIDA	RUBIACEAE	*Mastixiodendron plectocarpum*	LR/nt	NC
MAGNOLIOPSIDA	LAMIACEAE	*Gmelina peltata*	VU	NC
MAGNOLIOPSIDA	LAMIACEAE	*Gmelina salomonensis*	EN	NC
MAGNOLIOPSIDA	LAMIACEAE	*Mentha gattefossei*	VU	NC
MAGNOLIOPSIDA	LAMIACEAE	*Sideritis scardica*	NT	NC
MAGNOLIOPSIDA	LAMIACEAE	*Thymus saturejoides*	VU	NC
MAGNOLIOPSIDA	LAURACEAE	*Cinnamomum wightii*	EN	NC
MAGNOLIOPSIDA	LAURACEAE	*Eusideroxylon zwageri*	VU	NC
MAGNOLIOPSIDA	MAGNOLIACEAE	*Magnolia sinostellata*	EN	NC
MAGNOLIOPSIDA	MYRISTICACEAE	*Virola surinamensis*	EN	NC
MAGNOLIOPSIDA	CLUSIACEAE	*Garcinia indica*	VU	NC
MAGNOLIOPSIDA	EUPHORBIACEAE	*Cnidoscolus tepiquensis*	VU	NC
MAGNOLIOPSIDA	EUPHORBIACEAE	*Endospermum medullosum*	VU	NC
MAGNOLIOPSIDA	EUPHORBIACEAE	*Euphorbia bisglobosa*	EN	II
MAGNOLIOPSIDA	EUPHORBIACEAE	*Euphorbia capuronii*	CR	II
MAGNOLIOPSIDA	EUPHORBIACEAE	*Euphorbia geroldii*	CR	II
MAGNOLIOPSIDA	EUPHORBIACEAE	*Euphorbia gottlebei*	VU	II
MAGNOLIOPSIDA	EUPHORBIACEAE	*Euphorbia itremensis*	VU	II
MAGNOLIOPSIDA	EUPHORBIACEAE	*Euphorbia kondoi*	CR	II
MAGNOLIOPSIDA	EUPHORBIACEAE	*Euphorbia lophogona*	VU	II
MAGNOLIOPSIDA	DIPTEROCARPACEAE	*Anisoptera costata*	EN	NC
MAGNOLIOPSIDA	DIPTEROCARPACEAE	*Anisoptera curtisii*	VU	NC
MAGNOLIOPSIDA	DIPTEROCARPACEAE	*Anisoptera reticulata*	EN	NC

Class	Family	Species	Red List category	CITES Appendix
MAGNOLIOPSIDA	DIPTEROCARPACEAE	*Anisoptera scaphula*	EN	NC
MAGNOLIOPSIDA	DIPTEROCARPACEAE	*Anisoptera thurifera*	VU	NC
MAGNOLIOPSIDA	DIPTEROCARPACEAE	*Dipterocarpus acutangulus*	EN	NC
MAGNOLIOPSIDA	DIPTEROCARPACEAE	*Dipterocarpus alatus*	VU	NC
MAGNOLIOPSIDA	DIPTEROCARPACEAE	*Dipterocarpus caudiferus*	NT	NC
MAGNOLIOPSIDA	DIPTEROCARPACEAE	*Dipterocarpus chartaceus*	EN	NC
MAGNOLIOPSIDA	DIPTEROCARPACEAE	*Dipterocarpus costatus*	VU	NC
MAGNOLIOPSIDA	DIPTEROCARPACEAE	*Dipterocarpus costulatus*	NT	NC
MAGNOLIOPSIDA	DIPTEROCARPACEAE	*Dipterocarpus crinitus*	VU	NC
MAGNOLIOPSIDA	DIPTEROCARPACEAE	*Dipterocarpus cuspidatus*	CR	NC
MAGNOLIOPSIDA	DIPTEROCARPACEAE	*Dipterocarpus dyeri*	EN	NC
MAGNOLIOPSIDA	DIPTEROCARPACEAE	*Dipterocarpus gracilis*	VU	NC
MAGNOLIOPSIDA	DIPTEROCARPACEAE	*Dipterocarpus grandiflorus*	EN	NC
MAGNOLIOPSIDA	DIPTEROCARPACEAE	*Dipterocarpus hasseltii*	EN	NC
MAGNOLIOPSIDA	DIPTEROCARPACEAE	*Dipterocarpus intricatus*	EN	NC
MAGNOLIOPSIDA	DIPTEROCARPACEAE	*Dipterocarpus kerrii*	EN	NC
MAGNOLIOPSIDA	DIPTEROCARPACEAE	*Dipterocarpus mundus*	VU	NC
MAGNOLIOPSIDA	DIPTEROCARPACEAE	*Dipterocarpus retusus*	EN	NC
MAGNOLIOPSIDA	DIPTEROCARPACEAE	*Dipterocarpus tuberculatus*	NT	NC
MAGNOLIOPSIDA	DIPTEROCARPACEAE	*Dipterocarpus turbinatus*	VU	NC
MAGNOLIOPSIDA	DIPTEROCARPACEAE	*Dipterocarpus verrucosus*	NT	NC
MAGNOLIOPSIDA	DIPTEROCARPACEAE	*Dryobalanops sumatrensis*	VU	NC
MAGNOLIOPSIDA	DIPTEROCARPACEAE	*Hopea beccariana*	VU	NC
MAGNOLIOPSIDA	DIPTEROCARPACEAE	*Hopea fluvialis*	NT	NC
MAGNOLIOPSIDA	DIPTEROCARPACEAE	*Hopea griffithii*	EN	NC
MAGNOLIOPSIDA	DIPTEROCARPACEAE	*Hopea helferi*	EN	NC
MAGNOLIOPSIDA	DIPTEROCARPACEAE	*Hopea odorata*	VU	NC
MAGNOLIOPSIDA	DIPTEROCARPACEAE	*Hopea sangal*	VU	NC
MAGNOLIOPSIDA	DIPTEROCARPACEAE	*Hopea sublanceolata*	VU	NC
MAGNOLIOPSIDA	DIPTEROCARPACEAE	*Hopea sulcata*	NT	NC
MAGNOLIOPSIDA	DIPTEROCARPACEAE	*Neobalanocarpus heimii*	EN	NC
MAGNOLIOPSIDA	DIPTEROCARPACEAE	*Shorea albida*	VU	NC
MAGNOLIOPSIDA	DIPTEROCARPACEAE	*Shorea amplexicaulis*	NT	NC
MAGNOLIOPSIDA	DIPTEROCARPACEAE	*Shorea andulensis*	VU	NC
MAGNOLIOPSIDA	DIPTEROCARPACEAE	*Shorea angustifolia*	NT	NC
MAGNOLIOPSIDA	DIPTEROCARPACEAE	*Shorea atrinervosa*	VU	NC
MAGNOLIOPSIDA	DIPTEROCARPACEAE	*Shorea bentongensis*	VU	NC
MAGNOLIOPSIDA	DIPTEROCARPACEAE	*Shorea bracteolata*	EN	NC
MAGNOLIOPSIDA	DIPTEROCARPACEAE	*Shorea collina*	VU	NC
MAGNOLIOPSIDA	DIPTEROCARPACEAE	*Shorea coriacea*	NT	NC
MAGNOLIOPSIDA	DIPTEROCARPACEAE	*Shorea dispar*	CR	NC
MAGNOLIOPSIDA	DIPTEROCARPACEAE	*Shorea exelliptica*	NT	NC
MAGNOLIOPSIDA	DIPTEROCARPACEAE	*Shorea faguetiana*	EN	NC
MAGNOLIOPSIDA	DIPTEROCARPACEAE	*Shorea farinosa*	EN	NC
MAGNOLIOPSIDA	DIPTEROCARPACEAE	*Shorea ferruginea*	VU	NC

Class	Family	Species	Red List category	CITES Appendix
MAGNOLIOPSIDA	DIPTEROCARPACEAE	*Shorea foxworthyi*	VU	NC
MAGNOLIOPSIDA	DIPTEROCARPACEAE	*Shorea geniculata*	VU	NC
MAGNOLIOPSIDA	DIPTEROCARPACEAE	*Shorea glauca*	EN	NC
MAGNOLIOPSIDA	DIPTEROCARPACEAE	*Shorea gratissima*	EN	NC
MAGNOLIOPSIDA	DIPTEROCARPACEAE	*Shorea guiso*	VU	NC
MAGNOLIOPSIDA	DIPTEROCARPACEAE	*Shorea hemsleyana*	VU	NC
MAGNOLIOPSIDA	DIPTEROCARPACEAE	*Shorea henryana*	EN	NC
MAGNOLIOPSIDA	DIPTEROCARPACEAE	*Shorea hypochra*	EN	NC
MAGNOLIOPSIDA	DIPTEROCARPACEAE	*Shorea induplicata*	CR	NC
MAGNOLIOPSIDA	DIPTEROCARPACEAE	*Shorea laevis*	VU	NC
MAGNOLIOPSIDA	DIPTEROCARPACEAE	*Shorea leprosula*	NT	NC
MAGNOLIOPSIDA	DIPTEROCARPACEAE	*Shorea longisperma*	EN	NC
MAGNOLIOPSIDA	DIPTEROCARPACEAE	*Shorea macrantha*	CR	NC
MAGNOLIOPSIDA	DIPTEROCARPACEAE	*Shorea mecistopteryx*	VU	NC
MAGNOLIOPSIDA	DIPTEROCARPACEAE	*Shorea ochracea*	VU	NC
MAGNOLIOPSIDA	DIPTEROCARPACEAE	*Shorea pachyphylla*	EN	NC
MAGNOLIOPSIDA	DIPTEROCARPACEAE	*Shorea patoiensis*	NT	NC
MAGNOLIOPSIDA	DIPTEROCARPACEAE	*Shorea praestans*	CR	NC
MAGNOLIOPSIDA	DIPTEROCARPACEAE	*Shorea quadrinervis*	VU	NC
MAGNOLIOPSIDA	DIPTEROCARPACEAE	*Shorea rotundifolia*	CR	NC
MAGNOLIOPSIDA	DIPTEROCARPACEAE	*Shorea sagittata*	NT	NC
MAGNOLIOPSIDA	DIPTEROCARPACEAE	*Shorea scaberrima*	NT	NC
MAGNOLIOPSIDA	DIPTEROCARPACEAE	*Shorea singkawang*	VU	NC
MAGNOLIOPSIDA	DIPTEROCARPACEAE	*Shorea smithiana*	VU	NC
MAGNOLIOPSIDA	DIPTEROCARPACEAE	*Shorea stenoptera*	NT	NC
MAGNOLIOPSIDA	DIPTEROCARPACEAE	*Shorea sumatrana*	EN	NC
MAGNOLIOPSIDA	DIPTEROCARPACEAE	*Shorea superba*	VU	NC
MAGNOLIOPSIDA	DIPTEROCARPACEAE	*Shorea symingtonii*	VU	NC
MAGNOLIOPSIDA	DIPTEROCARPACEAE	*Shorea woodii*	EN	NC
MAGNOLIOPSIDA	MALVACEAE	*Adansonia grandidieri*	EN	II
MAGNOLIOPSIDA	MALVACEAE	*Gossypium mustelinum*	EN	NC
MAGNOLIOPSIDA	MALVACEAE	*Scaphium longiflorum*	VU	NC
MAGNOLIOPSIDA	THYMELAEACEAE	*Aquilaria beccariana*	VU	II
MAGNOLIOPSIDA	THYMELAEACEAE	*Aquilaria crassna*	CR	II
MAGNOLIOPSIDA	THYMELAEACEAE	*Aquilaria filaria*	VU	II
MAGNOLIOPSIDA	THYMELAEACEAE	*Aquilaria malaccensis*	CR	II
MAGNOLIOPSIDA	THYMELAEACEAE	*Aquilaria microcarpa*	EN	II
MAGNOLIOPSIDA	THYMELAEACEAE	*Aquilaria rostrata*	CR	II
MAGNOLIOPSIDA	THYMELAEACEAE	*Gonystylus affinis*	VU	II
MAGNOLIOPSIDA	THYMELAEACEAE	*Gonystylus bancanus*	CR	II
MAGNOLIOPSIDA	THYMELAEACEAE	*Gonystylus forbesii*	NT	II
MAGNOLIOPSIDA	THYMELAEACEAE	*Gonystylus maingayi*	VU	II
MAGNOLIOPSIDA	THYMELAEACEAE	*Gonystylus velutinus*	NT	II
MAGNOLIOPSIDA	COMBRETACEAE	*Terminalia brassii*	NT	NC
MAGNOLIOPSIDA	MYRTACEAE	*Eucalyptopsis papuana*	LR/nt	NC

Class	Family	Species	Red List category	CITES Appendix
MAGNOLIOPSIDA	MYRTACEAE	*Eucalyptus deglupta*	VU	NC
MAGNOLIOPSIDA	MYRTACEAE	*Xanthostemon melanoxylon*	VU	NC
MAGNOLIOPSIDA	DROSERACEAE	*Aldrovanda vesiculosa*	EN	NC
MAGNOLIOPSIDA	NEPENTHACEAE	*Nepenthes adnata*	EN	II
MAGNOLIOPSIDA	NEPENTHACEAE	*Nepenthes aristolochioides*	CR	II
MAGNOLIOPSIDA	NEPENTHACEAE	*Nepenthes clipeata*	CR	II
MAGNOLIOPSIDA	NEPENTHACEAE	*Nepenthes gantungensis*	VU	II
MAGNOLIOPSIDA	NEPENTHACEAE	*Nepenthes klossii*	EN	II
MAGNOLIOPSIDA	NEPENTHACEAE	*Nepenthes mapuluensis*	EN	II
MAGNOLIOPSIDA	NEPENTHACEAE	*Nepenthes palawanensis*	EN	II
MAGNOLIOPSIDA	NEPENTHACEAE	*Nepenthes paniculata*	EN	II
MAGNOLIOPSIDA	NEPENTHACEAE	*Nepenthes petiolata*	VU	II
MAGNOLIOPSIDA	NEPENTHACEAE	*Nepenthes pitopangii*	VU	II
MAGNOLIOPSIDA	NEPENTHACEAE	*Nepenthes rigidifolia*	CR	II
MAGNOLIOPSIDA	NEPENTHACEAE	*Nepenthes tenuis*	EN	II
MAGNOLIOPSIDA	CEPHALOTACEAE	*Cephalotus follicularis*	VU	NC
MAGNOLIOPSIDA	THEOPHRASTACEAE	*Clavija domingensis*	CR	NC
MAGNOLIOPSIDA	RANUNCULACEAE	*Aconitum chasmanthum*	CR	NC
MAGNOLIOPSIDA	RANUNCULACEAE	*Aconitum heterophyllum*	EN	NC
MAGNOLIOPSIDA	RANUNCULACEAE	*Aconitum violaceum*	VU	NC
MAGNOLIOPSIDA	RANUNCULACEAE	*Coptis teeta*	EN	NC
MAGNOLIOPSIDA	RANUNCULACEAE	*Hydrastis canadensis*	VU	II
MAGNOLIOPSIDA	MORACEAE	*Milicia excelsa*	LR/nt	NC
MAGNOLIOPSIDA	ROSACEAE	*Prunus africana*	VU	II
MAGNOLIOPSIDA	ULMACEAE	*Zelkova carpinifolia*	VU	NC
MAGNOLIOPSIDA	ULMACEAE	*Zelkova schneideriana*	VU	NC
MAGNOLIOPSIDA	ULMACEAE	*Zelkova serrata*	NT	NC
MAGNOLIOPSIDA	ULMACEAE	*Zelkova sinica*	VU	NC
MAGNOLIOPSIDA	SANTALACEAE	*Okoubaka aubrevillei*	EN	NC
MAGNOLIOPSIDA	SANTALACEAE	*Santalum album*	VU	NC
MAGNOLIOPSIDA	SANTALACEAE	*Santalum yasi*	EN	NC
MAGNOLIOPSIDA	ANACARDIACEAE	*Pistacia atlantica*	NT	NC
MAGNOLIOPSIDA	BURSERACEAE	*Boswellia ovalifoliolata*	VU	NC
MAGNOLIOPSIDA	BURSERACEAE	*Bursera linanoe*	VU	NC
MAGNOLIOPSIDA	BURSERACEAE	*Canarium luzonicum*	NT	NC
MAGNOLIOPSIDA	BURSERACEAE	*Commiphora guidottii*	VU	NC
MAGNOLIOPSIDA	BURSERACEAE	*Commiphora wightii*	CR	NC
MAGNOLIOPSIDA	BURSERACEAE	*Dacryodes buettneri*	VU	NC
MAGNOLIOPSIDA	MELIACEAE	*Cedrela fissilis*	VU	II
MAGNOLIOPSIDA	MELIACEAE	*Cedrela odorata*	VU	II
MAGNOLIOPSIDA	MELIACEAE	*Swietenia humilis*	EN	II
MAGNOLIOPSIDA	MELIACEAE	*Swietenia mahagoni*	NT	II
MAGNOLIOPSIDA	RUTACEAE	*Zanthoxylum flavum*	VU	NC
MAGNOLIOPSIDA	SAPINDACEAE	*Acer skutchii*	CR	NC
MAGNOLIOPSIDA	ACANTHACEAE	*Justicia leucoxiphos*	EN	NC

Class	Family	Species	Red List category	CITES Appendix
MAGNOLIOPSIDA	GESNERIACEAE	*Saintpaulia ulugurensis*	CR	NC
MAGNOLIOPSIDA	GESNERIACEAE	*Saintpaulia watkinsii*	CR	NC
MAGNOLIOPSIDA	GESNERIACEAE	*Streptocarpus myoporoides*	EN	NC
MAGNOLIOPSIDA	OLEACEAE	*Fraxinus latifolia*	NT	NC
MAGNOLIOPSIDA	OLEACEAE	*Syringa josikaea*	EN	NC
MAGNOLIOPSIDA	PEDALIACEAE	*Uncarina ankaranensis*	CR	NC
MAGNOLIOPSIDA	PEDALIACEAE	*Uncarina platycarpa*	CR	NC
MAGNOLIOPSIDA	PEDALIACEAE	*Uncarina turicana*	CR	NC
MAGNOLIOPSIDA	SCROPHULARIACEAE	*Neopicrorhiza minima*	EN	NC
MAGNOLIOPSIDA	THEACEAE	*Camellia azalea*	CR	NC
MAGNOLIOPSIDA	THEACEAE	*Camellia chrysanthoides*	EN	NC
MAGNOLIOPSIDA	THEACEAE	*Camellia euphlebia*	EN	NC
MAGNOLIOPSIDA	THEACEAE	*Camellia fangchengensis*	CR	NC
MAGNOLIOPSIDA	THEACEAE	*Camellia fascicularis*	CR	NC
MAGNOLIOPSIDA	THEACEAE	*Camellia flava*	CR	NC
MAGNOLIOPSIDA	THEACEAE	*Camellia granthamiana*	VU	NC
MAGNOLIOPSIDA	THEACEAE	*Camellia impressinervis*	CR	NC
MAGNOLIOPSIDA	THEACEAE	*Camellia longzhouensis*	EN	NC
MAGNOLIOPSIDA	THEACEAE	*Camellia nitidissima*	EN	NC
MAGNOLIOPSIDA	THEACEAE	*Camellia rosmannii*	CR	NC
MAGNOLIOPSIDA	THEACEAE	*Camellia subintegra*	NT	NC
MAGNOLIOPSIDA	FOUQUIERIACEAE	*Fouquieria columnaris*	VU	II
MAGNOLIOPSIDA	ZYGOPHYLLACEAE	*Gonopterodendron sarmientoi*	EN	II
MAGNOLIOPSIDA	ZYGOPHYLLACEAE	*Guaiacum officinale*	EN	II
MAGNOLIOPSIDA	ZYGOPHYLLACEAE	*Guaiacum sanctum*	NT	II
MALACOSTRACA	ASTACIDAE	*Astacus astacus*	VU	NC
MALACOSTRACA	ATYIDAE	*Caridina dennerli*	CR	NC
MALACOSTRACA	ATYIDAE	*Caridina glaubrechti*	CR	NC
MALACOSTRACA	ATYIDAE	*Caridina holthuisi*	EN	NC
MALACOSTRACA	ATYIDAE	*Caridina lanceolata*	CR	NC
MALACOSTRACA	ATYIDAE	*Caridina lingkonae*	CR	NC
MALACOSTRACA	ATYIDAE	*Caridina loehae*	CR	NC
MALACOSTRACA	ATYIDAE	*Caridina masapi*	CR	NC
MALACOSTRACA	ATYIDAE	*Caridina parvula*	CR	NC
MALACOSTRACA	ATYIDAE	*Caridina spinata*	CR	NC
MALACOSTRACA	ATYIDAE	*Caridina striata*	CR	NC
MALACOSTRACA	ATYIDAE	*Caridina tenuirostris*	CR	NC
MALACOSTRACA	ATYIDAE	*Caridina woltereckae*	CR	NC
MALACOSTRACA	GECARCINUCIDAE	*Nautilothelphusa zimmeri*	EN	NC
MALACOSTRACA	GECARCINUCIDAE	*Parathelphusa ferruginea*	EN	NC
MALACOSTRACA	GECARCINUCIDAE	*Parathelphusa pantherina*	EN	NC
MALACOSTRACA	GECARCINUCIDAE	*Syntripsa flavichela*	EN	NC
MALACOSTRACA	GECARCINUCIDAE	*Syntripsa matannensis*	EN	NC
MALACOSTRACA	PALAEMONIDAE	*Arachnochium kulsiense*	EN	NC
MALACOSTRACA	PALINURIDAE	*Palinurus elephas*	VU	NC

Class	Family	Species	Red List category	CITES Appendix
MAMMALIA	AILURIDAE	*Ailurus fulgens*	EN	I
MAMMALIA	FELIDAE	*Acinonyx jubatus*	VU	I
MAMMALIA	FELIDAE	*Catopuma badia*	EN	II
MAMMALIA	FELIDAE	*Catopuma temminckii*	NT	I
MAMMALIA	FELIDAE	*Felis bieti*	VU	II
MAMMALIA	FELIDAE	*Neofelis diardi*	VU	I
MAMMALIA	FELIDAE	*Neofelis nebulosa*	VU	I
MAMMALIA	FELIDAE	*Panthera leo*	VU	I/II
MAMMALIA	FELIDAE	*Panthera onca*	NT	I
MAMMALIA	FELIDAE	*Panthera pardus*	VU	I
MAMMALIA	FELIDAE	*Panthera tigris*	EN	I
MAMMALIA	FELIDAE	*Panthera uncia*	VU	I
MAMMALIA	MUSTELIDAE	*Enhydra lutris*	EN	II
MAMMALIA	MUSTELIDAE	*Lutra lutra*	NT	I
MAMMALIA	URSIDAE	*Helarctos malayanus*	VU	I
MAMMALIA	URSIDAE	*Melursus ursinus*	VU	I
MAMMALIA	URSIDAE	*Ursus maritimus*	VU	II
MAMMALIA	URSIDAE	*Ursus thibetanus*	VU	I
MAMMALIA	VIVERRIDAE	*Arctictis binturong*	VU	III
MAMMALIA	VIVERRIDAE	*Chrotogale owstoni*	EN	NC
MAMMALIA	VIVERRIDAE	*Viverra civettina*	CR	III
MAMMALIA	BALAENOPTERIDAE	*Balaenoptera borealis*	EN	I
MAMMALIA	BOVIDAE	*Addax nasomaculatus*	CR	I
MAMMALIA	BOVIDAE	*Bos gaurus*	VU	I
MAMMALIA	BOVIDAE	*Bos javanicus*	EN	NC
MAMMALIA	BOVIDAE	*Bos sauveli*	CR	I
MAMMALIA	BOVIDAE	*Bubalus arnee*	EN	III
MAMMALIA	BOVIDAE	*Capra falconeri*	NT	I
MAMMALIA	BOVIDAE	*Capricornis rubidus*	NT	I
MAMMALIA	BOVIDAE	*Capricornis sumatraensis*	VU	I
MAMMALIA	BOVIDAE	*Dorcatragus megalotis*	VU	NC
MAMMALIA	BOVIDAE	*Gazella arabica*	VU	NC
MAMMALIA	BOVIDAE	*Gazella cuvieri*	VU	I
MAMMALIA	BOVIDAE	*Gazella dorcas*	VU	III
MAMMALIA	BOVIDAE	*Gazella leptoceros*	EN	I
MAMMALIA	BOVIDAE	*Gazella spekei*	EN	NC
MAMMALIA	BOVIDAE	*Gazella subgutturosa*	VU	NC
MAMMALIA	BOVIDAE	*Naemorhedus baileyi*	VU	I
MAMMALIA	BOVIDAE	*Naemorhedus caudatus*	VU	I
MAMMALIA	BOVIDAE	*Nanger dama*	CR	I
MAMMALIA	BOVIDAE	*Oryx leucoryx*	VU	I
MAMMALIA	BOVIDAE	*Ovis ammon*	NT	II
MAMMALIA	BOVIDAE	*Pantholops hodgsonii*	NT	I
MAMMALIA	BOVIDAE	*Pelea capreolus*	NT	NC
MAMMALIA	BOVIDAE	*Pseudoryx nghetinhensis*	CR	I

Class	Family	Species	Red List category	CITES Appendix
MAMMALIA	BOVIDAE	*Redunca fulvorufula*	EN	NC
MAMMALIA	BOVIDAE	*Saiga tatarica*	CR	II
MAMMALIA	BOVIDAE	*Tetracerus quadricornis*	VU	III
MAMMALIA	BOVIDAE	*Tragelaphus buxtoni*	EN	NC
MAMMALIA	BOVIDAE	*Tragelaphus eurycerus*	NT	NC
MAMMALIA	BOVIDAE	*Tragelaphus imberbis*	NT	NC
MAMMALIA	CERVIDAE	*Axis porcinus*	EN	III
MAMMALIA	CERVIDAE	*Elaphodus cephalophus*	NT	NC
MAMMALIA	CERVIDAE	*Hippocamelus antisensis*	VU	I
MAMMALIA	CERVIDAE	*Ozotoceros bezoarticus*	NT	I
MAMMALIA	CERVIDAE	*Rangifer tarandus*	VU	NC
MAMMALIA	CERVIDAE	*Rucervus eldii*	EN	I
MAMMALIA	CERVIDAE	*Rusa timorensis*	VU	NC
MAMMALIA	CERVIDAE	*Rusa unicolor*	VU	NC
MAMMALIA	DELPHINIDAE	*Tursiops aduncus*	NT	II
MAMMALIA	HIPPOPOTAMIDAE	*Hippopotamus amphibius*	VU	II
MAMMALIA	MOSCHIDAE	*Moschus anhuiensis*	EN	II
MAMMALIA	MOSCHIDAE	*Moschus berezovskii*	EN	II
MAMMALIA	MOSCHIDAE	*Moschus chrysogaster*	EN	I/II
MAMMALIA	MOSCHIDAE	*Moschus cupreus*	EN	I
MAMMALIA	MOSCHIDAE	*Moschus fuscus*	EN	I/II
MAMMALIA	MOSCHIDAE	*Moschus leucogaster*	EN	I
MAMMALIA	MOSCHIDAE	*Moschus moschiferus*	VU	II
MAMMALIA	TAYASSUIDAE	*Tayassu pecari*	VU	II
MAMMALIA	PTEROPODIDAE	*Acerodon celebensis*	VU	II
MAMMALIA	PTEROPODIDAE	*Eidolon helvum*	NT	NC
MAMMALIA	PTEROPODIDAE	*Pteropus anetianus*	VU	II
MAMMALIA	PTEROPODIDAE	*Pteropus cognatus*	VU	II
MAMMALIA	PTEROPODIDAE	*Pteropus dasymallus*	VU	II
MAMMALIA	PTEROPODIDAE	*Pteropus fundatus*	EN	II
MAMMALIA	PTEROPODIDAE	*Pteropus nitendiensis*	EN	II
MAMMALIA	PTEROPODIDAE	*Pteropus pohlei*	VU	II
MAMMALIA	PTEROPODIDAE	*Pteropus rayneri*	NT	II
MAMMALIA	PTEROPODIDAE	*Pteropus rennelli*	EN	II
MAMMALIA	CHLAMYPHORIDAE	*Tolypeutes matacus*	NT	NC
MAMMALIA	LEPORIDAE	*Nesolagus timminsi*	EN	NC
MAMMALIA	TACHYGLOSSIDAE	*Zaglossus bartoni*	VU	II
MAMMALIA	TACHYGLOSSIDAE	*Zaglossus bruijnii*	CR	II
MAMMALIA	EQUIDAE	*Equus quagga*	NT	NC
MAMMALIA	RHINOCEROTIDAE	*Ceratotherium simum*	NT	I
MAMMALIA	RHINOCEROTIDAE	*Dicerorhinus sumatrensis*	CR	I
MAMMALIA	RHINOCEROTIDAE	*Diceros bicornis*	CR	I
MAMMALIA	RHINOCEROTIDAE	*Rhinoceros sondaicus*	CR	I
MAMMALIA	RHINOCEROTIDAE	*Rhinoceros unicornis*	VU	I
MAMMALIA	TAPIRIDAE	*Tapirus terrestris*	VU	II

Class	Family	Species	Red List category	CITES Appendix
MAMMALIA	MANIDAE	*Manis crassicaudata*	EN	I
MAMMALIA	MANIDAE	*Manis culionensis*	CR	I
MAMMALIA	MANIDAE	*Manis javanica*	CR	I
MAMMALIA	MANIDAE	*Manis pentadactyla*	CR	I
MAMMALIA	MANIDAE	*Phataginus tetradactyla*	VU	I
MAMMALIA	MANIDAE	*Phataginus tricuspis*	EN	I
MAMMALIA	MANIDAE	*Smutsia gigantea*	EN	I
MAMMALIA	MANIDAE	*Smutsia temminckii*	VU	I
MAMMALIA	AOTIDAE	*Aotus nancymaae*	VU	II
MAMMALIA	CERCOPITHECIDAE	*Cercopithecus roloway*	CR	I
MAMMALIA	CERCOPITHECIDAE	*Macaca arctoides*	VU	II
MAMMALIA	CERCOPITHECIDAE	*Macaca assamensis*	NT	II
MAMMALIA	CERCOPITHECIDAE	*Macaca nigra*	CR	II
MAMMALIA	CERCOPITHECIDAE	*Macaca sylvanus*	EN	I
MAMMALIA	CERCOPITHECIDAE	*Nasalis larvatus*	EN	I
MAMMALIA	CERCOPITHECIDAE	*Piliocolobus waldroni*	CR	II
MAMMALIA	CERCOPITHECIDAE	*Presbytis hosei*	VU	II
MAMMALIA	CERCOPITHECIDAE	*Presbytis melalophos*	EN	II
MAMMALIA	CERCOPITHECIDAE	*Presbytis mitrata*	VU	II
MAMMALIA	CERCOPITHECIDAE	*Presbytis potenziani*	CR	I
MAMMALIA	CERCOPITHECIDAE	*Procolobus verus*	VU	II
MAMMALIA	CERCOPITHECIDAE	*Pygathrix nemaeus*	EN	I
MAMMALIA	CERCOPITHECIDAE	*Pygathrix nigripes*	EN	I
MAMMALIA	CERCOPITHECIDAE	*Rhinopithecus strykeri*	CR	I
MAMMALIA	CERCOPITHECIDAE	*Semnopithecus priam*	NT	I
MAMMALIA	CERCOPITHECIDAE	*Trachypithecus francoisi*	EN	II
MAMMALIA	CERCOPITHECIDAE	*Trachypithecus shortridgei*	EN	I
MAMMALIA	HOMINIDAE	*Gorilla beringei*	CR	I
MAMMALIA	HOMINIDAE	*Pongo abelii*	CR	I
MAMMALIA	HOMINIDAE	*Pongo pygmaeus*	CR	I
MAMMALIA	HYLOBATIDAE	*Nomascus gabriellae*	EN	I
MAMMALIA	HYLOBATIDAE	*Nomascus annamensis*	EN	I
MAMMALIA	LORISIDAE	*Nycticebus coucang*	EN	I
MAMMALIA	LORISIDAE	*Nycticebus javanicus*	CR	I
MAMMALIA	LORISIDAE	*Nycticebus menagensis*	VU	I
MAMMALIA	LORISIDAE	*Nycticebus kayan*	VU	I
MAMMALIA	LORISIDAE	*Nycticebus pygmaeus*	VU	I
MAMMALIA	ELEPHANTIDAE	*Elephas maximus*	EN	I
MAMMALIA	ELEPHANTIDAE	*Loxodonta africana*	EN	I/II
MAMMALIA	ELEPHANTIDAE	*Loxodonta cyclotis*	CR	I
MAMMALIA	CHINCHILLIDAE	*Chinchilla chinchilla*	EN	I
MAMMALIA	CHINCHILLIDAE	*Chinchilla lanigera*	EN	I
MAMMALIA	SCIURIDAE	*Marmota sibirica*	EN	NC
MAMMALIA	TRICHECHIDAE	*Trichechus senegalensis*	VU	I
MEROSTOMATA	LIMULIDAE	*Limulus polyphemus*	VU	NC

Class	Family	Species	Red List category	CITES Appendix
MYXINI	MYXINIDAE	*Eptatretus burgeri*	NT	NC
PINOPSIDA	ARAUCARIACEAE	*Agathis borneensis*	EN	NC
PINOPSIDA	ARAUCARIACEAE	*Agathis dammara*	VU	NC
PINOPSIDA	ARAUCARIACEAE	*Agathis lanceolata*	VU	NC
PINOPSIDA	ARAUCARIACEAE	*Agathis moorei*	VU	NC
PINOPSIDA	ARAUCARIACEAE	*Agathis silbae*	NT	NC
PINOPSIDA	ARAUCARIACEAE	*Araucaria angustifolia*	CR	NC
PINOPSIDA	CUPRESSACEAE	*Chamaecyparis obtusa*	NT	NC
PINOPSIDA	CUPRESSACEAE	*Pilgerodendron uviferum*	VU	I
PINOPSIDA	CUPRESSACEAE	*Widdringtonia whytei*	CR	II
PINOPSIDA	PINACEAE	*Abies holophylla*	NT	NC
PINOPSIDA	PINACEAE	*Pinus greggii*	VU	NC
PINOPSIDA	PINACEAE	*Pinus merkusii*	VU	NC
PINOPSIDA	PODOCARPACEAE	*Nageia nagi*	NT	NC
PINOPSIDA	PODOCARPACEAE	*Podocarpus costalis*	EN	NC
PINOPSIDA	PODOCARPACEAE	*Podocarpus nubigenus*	NT	NC
PINOPSIDA	PODOCARPACEAE	*Podocarpus rumphii*	NT	NC
PINOPSIDA	PODOCARPACEAE	*Podocarpus teysmannii*	NT	NC
PINOPSIDA	TAXACEAE	*Amentotaxus yunnanensis*	VU	NC
PINOPSIDA	TAXACEAE	*Taxus chinensis*	EN	II
PINOPSIDA	TAXACEAE	*Taxus contorta*	EN	II
PINOPSIDA	TAXACEAE	*Taxus mairei*	VU	NC
PINOPSIDA	TAXACEAE	*Taxus wallichiana*	EN	II
REPTILIA	CROCODYLIDAE	*Crocodylus acutus*	VU	I/II
REPTILIA	CROCODYLIDAE	*Crocodylus siamensis*	CR	I
REPTILIA	CROCODYLIDAE	*Mecistops cataphractus*	CR	I
REPTILIA	AGAMIDAE	*Lyriocephalus scutatus*	NT	II
REPTILIA	AGAMIDAE	*Malayodracon robinsonii*	NT	NC
REPTILIA	AGAMIDAE	*Physignathus cocincinus*	VU	NC
REPTILIA	AGAMIDAE	*Trapelus savignii*	VU	NC
REPTILIA	AGAMIDAE	*Uromastyx aegyptia*	VU	II
REPTILIA	AGAMIDAE	*Uromastyx shobraki*	NT	II
REPTILIA	AGAMIDAE	*Uromastyx thomasi*	VU	II
REPTILIA	AGAMIDAE	*Uromastyx yemenensis*	NT	II
REPTILIA	ANGUIDAE	*Abronia campbelli*	CR	I
REPTILIA	ANGUIDAE	*Abronia mixteca*	VU	II
REPTILIA	ANGUIDAE	*Abronia taeniata*	VU	II
REPTILIA	BOIDAE	*Boa orophias*	EN	II
REPTILIA	CARPHODACTYLIDAE	*Orraya occultus*	VU	NC
REPTILIA	CARPHODACTYLIDAE	*Phyllurus amnicola*	NT	NC
REPTILIA	CARPHODACTYLIDAE	*Phyllurus caudiannulatus*	NT	NC
REPTILIA	CARPHODACTYLIDAE	*Phyllurus pinnaclensis*	CR	NC
REPTILIA	CHAMAELEONIDAE	*Bradypodion caeruleogula*	EN	II
REPTILIA	CHAMAELEONIDAE	*Bradypodion thamnobates*	EN	II
REPTILIA	CHAMAELEONIDAE	*Brookesia perarmata*	EN	I

Class	Family	Species	Red List category	CITES Appendix
REPTILIA	CHAMAELEONIDAE	*Brookesia valerieae*	EN	II
REPTILIA	CHAMAELEONIDAE	*Calumma gallus*	EN	II
REPTILIA	CHAMAELEONIDAE	*Calumma parsonii*	NT	II
REPTILIA	CHAMAELEONIDAE	*Furcifer belalandaensis*	CR	II
REPTILIA	CHAMAELEONIDAE	*Furcifer minor*	EN	II
REPTILIA	CHAMAELEONIDAE	*Kinyongia carpenteri*	NT	II
REPTILIA	CHAMAELEONIDAE	*Kinyongia fischeri*	NT	II
REPTILIA	CHAMAELEONIDAE	*Kinyongia multituberculata*	EN	II
REPTILIA	CHAMAELEONIDAE	*Kinyongia xenorhina*	NT	II
REPTILIA	CHAMAELEONIDAE	*Rhampholeon acuminatus*	CR	II
REPTILIA	CHAMAELEONIDAE	*Rhampholeon spinosus*	EN	II
REPTILIA	CHAMAELEONIDAE	*Rhampholeon viridis*	EN	II
REPTILIA	CHAMAELEONIDAE	*Trioceros feae*	NT	II
REPTILIA	CHAMAELEONIDAE	*Trioceros montium*	NT	II
REPTILIA	CHAMAELEONIDAE	*Trioceros perreti*	EN	II
REPTILIA	CHAMAELEONIDAE	*Trioceros pfefferi*	EN	II
REPTILIA	CHAMAELEONIDAE	*Trioceros quadricornis*	VU	II
REPTILIA	CHAMAELEONIDAE	*Trioceros serratus*	VU	II
REPTILIA	COLUBRIDAE	*Elaphe moellendorffi*	VU	NC
REPTILIA	COLUBRIDAE	*Euprepiophis perlacea*	EN	NC
REPTILIA	COLUBRIDAE	*Lampropeltis ruthveni*	NT	NC
REPTILIA	CORDYLIDAE	*Platysaurus imperator*	VU	NC
REPTILIA	CORDYLIDAE	*Smaug giganteus*	VU	II
REPTILIA	DACTYLOIDAE	*Anolis proboscis*	EN	NC
REPTILIA	DIPLODACTYLIDAE	*Mokopirirakau granulatus*	VU	III
REPTILIA	DIPLODACTYLIDAE	*Naultinus elegans*	VU	II
REPTILIA	DIPLODACTYLIDAE	*Naultinus gemmeus*	EN	II
REPTILIA	DIPLODACTYLIDAE	*Naultinus grayii*	NT	II
REPTILIA	DIPLODACTYLIDAE	*Naultinus stellatus*	VU	II
REPTILIA	DIPLODACTYLIDAE	*Naultinus tuberculatus*	VU	II
REPTILIA	DIPLODACTYLIDAE	*Rhacodactylus trachyrhynchus*	EN	NC
REPTILIA	DIPLODACTYLIDAE	*Tukutuku rakiurae*	EN	III
REPTILIA	DIPSADIDAE	*Alsophis antiguae*	CR	NC
REPTILIA	DIPSADIDAE	*Atractus gigas*	NT	NC
REPTILIA	ELAPIDAE	*Naja atra*	VU	II
REPTILIA	ELAPIDAE	*Naja mandalayensis*	VU	II
REPTILIA	ELAPIDAE	*Naja philippinensis*	NT	II
REPTILIA	ELAPIDAE	*Naja siamensis*	VU	II
REPTILIA	ELAPIDAE	*Ophiophagus hannah*	VU	II
REPTILIA	EUBLEPHARIDAE	*Goniurosaurus bawanglingensis*	EN	II
REPTILIA	EUBLEPHARIDAE	*Goniurosaurus catbaensis*	EN	II
REPTILIA	EUBLEPHARIDAE	*Goniurosaurus huuliensis*	CR	II
REPTILIA	EUBLEPHARIDAE	*Goniurosaurus kuroiwae*	VU	III
REPTILIA	EUBLEPHARIDAE	*Goniurosaurus liboensis*	EN	II

Class	Family	Species	Red List category	CITES Appendix
REPTILIA	EUBLEPHARIDAE	*Goniurosaurus lichtenfelderi*	VU	II
REPTILIA	EUBLEPHARIDAE	*Goniurosaurus splendens*	EN	III
REPTILIA	EUBLEPHARIDAE	*Goniurosaurus yingdeensis*	CR	II
REPTILIA	EUBLEPHARIDAE	*Goniurosaurus zhelongi*	EN	II
REPTILIA	GEKKONIDAE	*Cnemaspis psychedelica*	EN	I
REPTILIA	GEKKONIDAE	*Cyrtodactylus pulchellus*	EN	NC
REPTILIA	GEKKONIDAE	*Cyrtodactylus salomonensis*	NT	NC
REPTILIA	GEKKONIDAE	*Gekko badenii*	EN	NC
REPTILIA	GEKKONIDAE	*Gekko swinhonis*	VU	NC
REPTILIA	GEKKONIDAE	*Lygodactylus williamsi*	CR	I
REPTILIA	GEKKONIDAE	*Phelsuma flavigularis*	EN	II
REPTILIA	GEKKONIDAE	*Phelsuma pronki*	CR	II
REPTILIA	GEKKONIDAE	*Phelsuma serraticauda*	EN	II
REPTILIA	GEKKONIDAE	*Phelsuma standingi*	VU	II
REPTILIA	GEKKONIDAE	*Uroplatus henkeli*	VU	II
REPTILIA	GEKKONIDAE	*Uroplatus pietschmanni*	EN	II
REPTILIA	GERRHOSAURIDAE	*Zonosaurus maximus*	VU	NC
REPTILIA	IGUANIDAE	*Ctenosaura bakeri*	CR	II
REPTILIA	IGUANIDAE	*Ctenosaura defensor*	VU	II
REPTILIA	IGUANIDAE	*Ctenosaura melanosterna*	EN	II
REPTILIA	IGUANIDAE	*Ctenosaura nolascensis*	VU	II
REPTILIA	IGUANIDAE	*Ctenosaura oedirhina*	EN	II
REPTILIA	IGUANIDAE	*Ctenosaura palearis*	EN	II
REPTILIA	IGUANIDAE	*Ctenosaura quinquecarinata*	EN	II
REPTILIA	IGUANIDAE	*Cyclura cychlura*	VU	I
REPTILIA	IGUANIDAE	*Sauromalus hispidus*	EN	NC
REPTILIA	IGUANIDAE	*Sauromalus varius*	VU	I
REPTILIA	LACERTIDAE	*Acanthodactylus pardalis*	VU	NC
REPTILIA	LACERTIDAE	*Iberolacerta martinezricai*	CR	NC
REPTILIA	LACERTIDAE	*Podarcis lilfordi*	EN	II
REPTILIA	LEIOSAURIDAE	*Pristidactylus casuhatiensis*	CR	NC
REPTILIA	LEIOSAURIDAE	*Pristidactylus valeriae*	EN	NC
REPTILIA	LEIOSAURIDAE	*Pristidactylus volcanensis*	EN	NC
REPTILIA	PHYLLODACTYLIDAE	*Tarentola chazaliae*	VU	NC
REPTILIA	PHYLLODACTYLIDAE	*Tarentola gigas*	EN	NC
REPTILIA	PSEUDOXYRHOPHIIDAE	*Lycodryas citrinus*	VU	NC
REPTILIA	PYTHONIDAE	*Python bivittatus*	VU	II
REPTILIA	SCINCIDAE	*Larutia trifasciata*	NT	NC
REPTILIA	SCINCIDAE	*Trachylepis loluiensis*	VU	NC
REPTILIA	SHINISAURIDAE	*Shinisaurus crocodilurus*	EN	I
REPTILIA	SPHAERODACTYLIDAE	*Gonatodes daudini*	CR	I
REPTILIA	TROPIDOPHIIDAE	*Tropidophis caymanensis*	CR	II
REPTILIA	VARANIDAE	*Varanus olivaceus*	VU	II
REPTILIA	VIPERIDAE	*Atheris ceratophora*	VU	NC
REPTILIA	VIPERIDAE	*Atheris desaixi*	EN	II

Class	Family	Species	Red List category	CITES Appendix
REPTILIA	VIPERIDAE	*Bitis armata*	VU	NC
REPTILIA	VIPERIDAE	*Bothriechis aurifer*	VU	NC
REPTILIA	VIPERIDAE	*Bothriechis marchi*	EN	NC
REPTILIA	VIPERIDAE	*Crotalus catalinensis*	CR	NC
REPTILIA	VIPERIDAE	*Cryptelytrops kanburiensis*	EN	NC
REPTILIA	VIPERIDAE	*Daboia deserti*	NT	NC
REPTILIA	VIPERIDAE	*Macrovipera schweizeri*	EN	NC
REPTILIA	VIPERIDAE	*Montivipera albizona*	EN	NC
REPTILIA	VIPERIDAE	*Montivipera raddei*	NT	NC
REPTILIA	VIPERIDAE	*Montivipera wagneri*	CR	II
REPTILIA	VIPERIDAE	*Popeia buniana*	EN	NC
REPTILIA	VIPERIDAE	*Popeia nebularis*	VU	NC
REPTILIA	VIPERIDAE	*Protobothrops mangshanensis*	EN	NC
REPTILIA	VIPERIDAE	*Protobothrops tokarensis*	VU	NC
REPTILIA	VIPERIDAE	*Vipera anatolica*	CR	NC
REPTILIA	VIPERIDAE	*Vipera barani*	NT	NC
REPTILIA	VIPERIDAE	*Vipera dinniki*	VU	NC
REPTILIA	VIPERIDAE	*Vipera kaznakovi*	EN	NC
REPTILIA	VIPERIDAE	*Vipera orlovi*	CR	NC
REPTILIA	VIPERIDAE	*Vipera transcaucasiana*	NT	NC
REPTILIA	VIPERIDAE	*Vipera ursinii*	VU	I
REPTILIA	XENOSAURIDAE	*Xenosaurus grandis*	VU	NC
REPTILIA	CARETTOCHELYIDAE	*Carettochelys insculpta*	EN	II
REPTILIA	CHELIDAE	*Acanthochelys pallidipectoris*	EN	NC
REPTILIA	CHELIDAE	*Chelodina mccordi*	CR	II
REPTILIA	CHELIDAE	*Chelodina parkeri*	NT	NC
REPTILIA	CHELIDAE	*Chelodina pritchardi*	VU	NC
REPTILIA	CHELIDAE	*Chelodina reimanni*	LR/nt	NC
REPTILIA	CHELIDAE	*Elseya branderhorsti*	VU	NC
REPTILIA	CHELIDAE	*Mesoclemmys hogei*	CR	NC
REPTILIA	CHELIDAE	*Phrynops williamsi*	VU	NC
REPTILIA	CHELONIIDAE	*Eretmochelys imbricata*	CR	I
REPTILIA	CHELYDRIDAE	*Macrochelys temminckii*	VU	III
REPTILIA	EMYDIDAE	*Clemmys guttata*	EN	II
REPTILIA	EMYDIDAE	*Emydoidea blandingii*	EN	II
REPTILIA	EMYDIDAE	*Graptemys barbouri*	VU	III
REPTILIA	EMYDIDAE	*Graptemys ernsti*	NT	III
REPTILIA	EMYDIDAE	*Graptemys gibbonsi*	EN	III
REPTILIA	EMYDIDAE	*Graptemys oculifera*	VU	III
REPTILIA	EMYDIDAE	*Graptemys pearlensis*	EN	III
REPTILIA	EMYDIDAE	*Malaclemys terrapin*	VU	II
REPTILIA	EMYDIDAE	*Pseudemys gorzugi*	NT	NC
REPTILIA	GEOEMYDIDAE	*Batagur affinis*	CR	I
REPTILIA	GEOEMYDIDAE	*Batagur baska*	CR	I
REPTILIA	GEOEMYDIDAE	*Batagur dhongoka*	CR	II

Class	Family	Species	Red List category	CITES Appendix
REPTILIA	GEOEMYDIDAE	*Batagur kachuga*	CR	II
REPTILIA	GEOEMYDIDAE	*Batagur trivittata*	CR	II
REPTILIA	GEOEMYDIDAE	*Cuora bourreti*	CR	I
REPTILIA	GEOEMYDIDAE	*Cuora galbinifrons*	CR	II
REPTILIA	GEOEMYDIDAE	*Cuora picturata*	CR	I
REPTILIA	GEOEMYDIDAE	*Cuora trifasciata*	CR	II
REPTILIA	GEOEMYDIDAE	*Cuora yunnanensis*	CR	II
REPTILIA	GEOEMYDIDAE	*Cyclemys dentata*	NT	II
REPTILIA	GEOEMYDIDAE	*Cyclemys atripons*	EN	II
REPTILIA	GEOEMYDIDAE	*Cyclemys enigmatica*	NT	II
REPTILIA	GEOEMYDIDAE	*Cyclemys gmeli*	NT	II
REPTILIA	GEOEMYDIDAE	*Cyclemys oldhamii*	EN	II
REPTILIA	GEOEMYDIDAE	*Cyclemys pulchristriata*	EN	II
REPTILIA	GEOEMYDIDAE	*Geoclemys hamiltonii*	EN	I
REPTILIA	GEOEMYDIDAE	*Orlitia borneensis*	EN	II
REPTILIA	GEOEMYDIDAE	*Siebenrockiella crassicollis*	VU	II
REPTILIA	PELOMEDUSIDAE	*Pelusios niger*	NT	NC
REPTILIA	TESTUDINIDAE	*Astrochelys radiata*	CR	I
REPTILIA	TESTUDINIDAE	*Astrochelys yniphora*	CR	I
REPTILIA	TESTUDINIDAE	*Chelonoidis chathamensis*	EN	I
REPTILIA	TESTUDINIDAE	*Chelonoidis guntheri*	CR	I
REPTILIA	TESTUDINIDAE	*Chersobius signatus*	EN	II
REPTILIA	TESTUDINIDAE	*Geochelone elegans*	VU	I
REPTILIA	TESTUDINIDAE	*Indotestudo elongata*	CR	II
REPTILIA	TESTUDINIDAE	*Kinixys homeana*	VU	II
REPTILIA	TESTUDINIDAE	*Kinixys lobatsiana*	VU	II
REPTILIA	TESTUDINIDAE	*Kinixys natalensis*	VU	II
REPTILIA	TESTUDINIDAE	*Malacochersus tornieri*	CR	I
REPTILIA	TESTUDINIDAE	*Manouria emys*	CR	II
REPTILIA	TESTUDINIDAE	*Psammobates geometricus*	CR	I
REPTILIA	TESTUDINIDAE	*Psammobates tentorius*	NT	II
REPTILIA	TESTUDINIDAE	*Pyxis arachnoides*	CR	I
REPTILIA	TESTUDINIDAE	*Pyxis planicauda*	CR	I
REPTILIA	TESTUDINIDAE	*Testudo hermanni*	NT	II
REPTILIA	TESTUDINIDAE	*Testudo kleinmanni*	CR	I
REPTILIA	TRIONYCHIDAE	*Chitra chitra*	CR	I
REPTILIA	TRIONYCHIDAE	*Cyclanorbis senegalensis*	VU	II
REPTILIA	TRIONYCHIDAE	*Cycloderma aubryi*	VU	II
REPTILIA	TRIONYCHIDAE	*Cycloderma frenatum*	EN	II
REPTILIA	TRIONYCHIDAE	*Pelochelys signifera*	VU	II

Supplementary Table 2 | Number of threatened and Near Threatened species in categories Likely, Insufficient information and Unlikely with coded BRU threats.

Class	Total species	Category		
		Likely	Insufficient information	Unlikely
Actinopterygii	1289	257	348	684
Agaricomycetes	32		2	30
Amphibia	1535	97	70	1368
Anthozoa	403	200	199	4
Arachnida	19	18		1
Arthoniomycetes	1			1
Aves	1252	202	313	737
Bivalvia	44	2	8	34
Bryopsida	18		5	13
Cephalopoda	7	1	1	5
Chondrichthyes	315	129	112	74
Clitellata	1		1	
Cycadopsida	161	105	45	11
Diplopoda	47			47
Enopla	1			1
Florideophyceae	1			1
Gastropoda	347	42	81	224
Ginkgoopsida	1		1	
Gnetopsida	7	2	3	2
Holothuroidea	14	11	3	
Hydrozoa	6	6		
Insecta	601	15	49	537
Jungermanniopsida	18		1	17
Lecanoromycetes	8		3	5
Leotiomycetes	1			1
Liliopsida	1420	340	526	554
Lycopodiopsida	4		1	3
Magnoliopsida	4812	398	2121	2293
Malacostraca	160	20	39	101
Mammalia	1126	134	146	846
Marchantiopsida	3			3
Merostomata	2	1	1	
Myxini	6	1	3	2
Onychophora	3		1	2
Pezizomycetes	1			1
Phaeophyceae	5			5
Pinopsida	205	22	53	130
Polypodiopsida	35		17	18
Reptilia	837	191	183	463
Sarcopterygii	1		1	
TOTAL	14741	2194	4337	8210

Supplementary Table 3 | Comparison of the number of Actinopterygii and Amphibia species categorised as Likely, Insufficient information and Unlikely based on automated and manual coding. Grey cells indicate the number of species given the same code using both methods.

		**Automated coding**		
		Likely	Insufficient information	Unlikely
**Manual coding**	Likely	221	57	2
	Insufficient information	59	358	57
	Unlikely	7	81	674

Supplementary Table 4 | Number of Actinopterygii and Amphibia species with new/updated assessments assigned to the categories Likely, Unlikely and Insufficient information based on assessments published in version 2020-1 of the Red List ("previous assessment", based on manual coding) and those published in version 2021-2 ("new assessments", based on automated coding). Grey cells indicate the number of species that did not change category following the publication of a new assessment.

		**New assessment (v 2021-2)**		
		Likely	Insufficient information	Unlikely
**Previous assessment (v 2020-1)**	Likely	46	13	3
	Insufficient information	8	51	14
	Unlikely	8	18	351
	No previous classification	40	244	511

Supplementary Table 5 | Species of Actinopterygii and Amphibia newly assessed or reassessed since Red List version 2020-1 was published that were classified as Likely threatened by international trade using advanced automated coding (n=54 species). *Indicates the species was coded Likely based on their previous assessment using the automated process.

Class	Family	Species	Red List category	CITES Appendix	Former category
ACTINOPTERYGII	ANGUILLIDAE	*Anguilla borneensis*	VU	NC	I*
ACTINOPTERYGII	CALLICHTHYIDAE	*Corydoras habrosus*	NT	NC	NA
ACTINOPTERYGII	CALLICHTHYIDAE	*Corydoras metae*	VU	NC	NA
ACTINOPTERYGII	CICHLIDAE	*Coptodon discolor*	NT	NC	U
ACTINOPTERYGII	CICHLIDAE	*Nandopsis ramsdeni*	EN	NC	NA
ACTINOPTERYGII	CYPRINIDAE	*Balantiocheilos melanopterus*	VU	NC	NA
ACTINOPTERYGII	CYPRINIDAE	*Brevibora exilis*	NT	NC	NA
ACTINOPTERYGII	CYPRINIDAE	*Desmopuntius foerschi*	NT	NC	NA
ACTINOPTERYGII	CYPRINIDAE	*Fangfangia spinicleithralis*	NT	NC	NA
ACTINOPTERYGII	CYPRINIDAE	*Paedocypris carbunculus*	NT	NC	NA
ACTINOPTERYGII	CYPRINIDAE	*Schizocypris brucei*	VU	NC	NA
ACTINOPTERYGII	GASTROMYZONTIDAE	*Gastromyzon katibasensis*	NT	NC	NA
ACTINOPTERYGII	GASTROMYZONTIDAE	*Gastromyzon stellatus*	NT	NC	NA
ACTINOPTERYGII	GASTROMYZONTIDAE	*Gastromyzon viriosus*	NT	NC	NA
ACTINOPTERYGII	GOBIIDAE	*Lentipes dimetrodon*	VU	NC	NA
ACTINOPTERYGII	GOBIIDAE	*Lentipes watsoni*	EN	NC	NA
ACTINOPTERYGII	GOBIIDAE	*Sicyopterus calliochromus*	NT	NC	NA
ACTINOPTERYGII	HORABAGRIDAE	*Horabagrus brachysoma*	VU	NC	I*
ACTINOPTERYGII	LEPISOSTEIDAE	*Atractosteus tristoechus*	CR	NC	NA
ACTINOPTERYGII	MELANOTAENIIDAE	*Melanotaenia lacustris*	EN	NC	NA
ACTINOPTERYGII	OSPHRONEMIDAE	*Pseudosphromenus dayi*	VU	NC	I*
ACTINOPTERYGII	RIVULIDAE	*Renova oscari*	VU	NC	NA
ACTINOPTERYGII	SALMONIDAE	*Brachymystax savinovi*	EN	NC	NA
ACTINOPTERYGII	SCIAENIDAE	*Bahaba taipingensis*	CR	NC	I
ACTINOPTERYGII	SCIAENIDAE	*Cynoscion acoupa*	VU	NC	NA
ACTINOPTERYGII	SILURIDAE	*Ompok bimaculatus*	NT	NC	I*
ACTINOPTERYGII	TETRAODONTIDE	*Carinotetraodon salivator*	NT	NC	NA
ACTINOPTERYGII	ZENARCHOPTERIDAE	*Dermogenys orientalis*	VU	NC	NA
ACTINOPTERYGII	ZENARCHOPTERIDAE	*Nomorhamphus kolonodalensis*	NT	NC	NA
ACTINOPTERYGII	ZENARCHOPTERIDAE	*Nomorhamphus liemi*	NT	NC	NA
AMPHIBIA	AMBYSTOMATIDAE	*Ambystoma altamirani*	EN	NC	U
AMPHIBIA	AMBYSTOMATIDAE	*Ambystoma granulosum*	EN	NC	NA
AMPHIBIA	AMBYSTOMATIDAE	*Ambystoma leorae*	CR	NC	U
AMPHIBIA	AMBYSTOMATIDAE	*Ambystoma ordinarium*	EN	NC	NA
AMPHIBIA	HYNOBIIDAE	*Hynobius boulengeri*	EN	NC	NA
AMPHIBIA	HYNOBIIDAE	*Hynobius tsurugiensis*	EN	NC	NA

AMPHIBIA	HYNOBIIDAE	*Pseudohynobius shuichengensis*	CR	NC	U
AMPHIBIA	MANTELLIDAE	*Mantella aurantiaca*	EN	II	I*
AMPHIBIA	MEGOPHRYIDAE	*Leptobrachium boringii*	EN	NC	U
AMPHIBIA	PLETHODONTIDAE	*Bolitoglossa conanti*	VU	NC	U
AMPHIBIA	PLETHODONTIDAE	*Bolitoglossa dofleini*	NT	NC	I*
AMPHIBIA	PLETHODONTIDAE	*Chiropterotriton multidentatus*	EN	NC	NA
AMPHIBIA	SALMANDRIDAE	*Cynops pyrrhogaster*	NT	NC	NA
AMPHIBIA	SALMANDRIDAE	*Cynops yunnanensis*	VU	NC	NA
AMPHIBIA	SALMANDRIDAE	*Echinotriton maxiquadratus*	CR	NC	NA
AMPHIBIA	SALMANDRIDAE	*Pachytriton changi*	EN	NC	NA
AMPHIBIA	SALMANDRIDAE	*Pachytriton xanthospilos*	CR	NC	NA
AMPHIBIA	SALMANDRIDAE	*Paramesotriton caudopunctatus*	NT	II	NA
AMPHIBIA	SALMANDRIDAE	*Paramesotriton longliensis*	VU	II	NA
AMPHIBIA	SALMANDRIDAE	*Paramesotriton yunwuensis*	EN	II	NA
AMPHIBIA	SALMANDRIDAE	*Paramesotriton zhijinensis*	EN	II	NA
AMPHIBIA	SALMANDRIDAE	*Tylototriton lizhengchangi*	CR	II	NA
AMPHIBIA	SALMANDRIDAE	*Tylototriton yangi*	EN	II	NA
AMPHIBIA	TELMATOBIIDAE	*Telmatobius culeus*	EN	I	U

Supplementary Table 6 | Comparison of the number of animal species categorised as Likely, Insufficient information and Unlikely based on automated and manual coding. Grey cells indicate the number of species given the same code using both methods.

		**Automated coding**		
		Likely	Insufficient information	Unlikely
**Manual coding**	Likely	622	453	40
	Insufficient information	160	1258	269
	Unlikely	17	276	2237

4. Supplementary Results

4.1 Proportion of species Likely threatened by international trade

Proportion of globally threatened and Near Threatened species

Our results suggest that a relatively small proportion of globally threatened and Near Threatened species are Likely threatened by international trade, but the proportion increases when considering uncertainty within our dataset. Incorporating uncertainty that species categorised as having Insufficient information introduce, the percentage and number of species Likely threatened by international trade is, lower bound: 5.8% (2,211 species), mid-point: 15% (5,737 species), and upper bound: 23% (8,796 species).

Proportion of species with coded BRU threats

The 14,741 species with coded BRU threats in our dataset represent 87% of the species with these coded threats on the Red List. Incorporating the uncertainty that species categorised as Insufficient information introduce, the percentage and number of species Likely threatened by international trade as a proportion of species with coded BRU threats is, lower bound: 15% (2,194 species), mid-point: 21% (3,096 species), and upper bound: 44% (6,486 species). The lower bound is 2,194 species and not 2,211 because 17 species were coded Likely despite not having coded BRU threats.

4.2 Proportion of species Likely threatened by international trade – intentional use

The proportion of species in classes with BRU threats that are also Likely threatened by international trade is relatively small (Fig. 2) but increases for most groups when considering intentional use only (Extended Data Fig. 3). Across taxonomic groups the proportion of species with coded BRU threats that are also Likely threatened by international trade is 15% (as above). Considering intentional use only, the proportion of species that have intentional

BRU threats coded and are Likely threatened by international trade is 29% (2,093/7,243 species – excluding 118 species Likely threatened by international trade that do not have BRU threats coded (17 species) or intentional BRU threats coded (101 species).

4.3 Retrospective coding results

Retrospective coding of Actinopterygii and Amphibia species

When applied to the 1,516 species of Actinopterygii (n=1187) and Amphibia (n=329) that had been manually coded (Supplementary Table 3) the automated coding method detailed in Supplementary Methods 2.8, returned the same categorisation in 82.7% (1,253/1,516 species) of cases. Fleiss' Kappa was $\kappa = 0.72$ (95% CI: 0.68–0.75), p .000, indicating a high level of agreement[42] between the manual and automated coding. Including the species initially coded with the automated method (Supplementary Methods 2.4 (*Automated coding*)), i.e., all initial coding of species in these classes, 92% of 3,623 species in Actinopterygii and Amphibia received the same code as the manual coding. Fleiss' Kappa was $\kappa = 0.82$ (95% CI: 0.80–0.85), p .000, indicating almost perfect agreement[42] and demonstrating that the automated coding performed well.

Classification of new and updated Red List assessments

Of the Red List version 2021-2 assessments, 1,307 species of Actinopterygii and Amphibia that met the initial selection criteria (Methods and Supplementary Methods 2.2) had been newly assessed for the Red List since the data were downloaded for this paper (which used Red List version 2020-1). Of these, 512 species had previously been categorised using our criteria, and the automated coding accurately coded 87.5% of species (Supplementary Table 4). Of these 512 species, 64 were placed in a different category from their previous categorisation based on manual coding (Supplementary Table 4). Based on these new

assessments, a further 54 Actinopterygii (n=30) and Amphibia (n=24) species were categorised as Likely to be threatened by international trade in addition to the manually coded species in Supplementary Table 1 (see Supplementary Table 5). It should be noted that 6 of these species had already been coded as Likely using the automated coding approach based on the previous assessments, and so the comparison with the previous manual coding may overinflate the difference.

Classification of broader taxonomic groups

Applying the advanced automated coding method in Supplementary Methods 2.8 (as tested for Actinopterygii and Amphibia) to all animals (kingdom Animalia) resulted in 77% accuracy compared with the manual coding (n=5,332 species) (Supplementary Table 6). Fleiss' Kappa $\kappa = 0.6$ (95% CI: 0.58–0.62, p .000), indicating moderate agreement[42]. This is slightly lower than the 82.7% match for the classes Actinopterygii and Amphibia against which the code was tested, indicating a need to develop additional keyword strings specific to the other taxonomic groups. However, where class-specific keyword strings are used, as demonstrated for Actinopterygii and Amphibia, the accuracy of automated coding compared with manual coding can reach >80% and be comparable to manual coding by a human being.

5. Supplementary Discussion

5.1 Application of results

Species Likely threatened by international trade

The species in this category were so included because they met the relevant criteria (see Methods). However, the placement of species in this category does not imply that they are necessarily threatened by international trade at the global level, or that international trade constitutes a major threat to the species, or that in instances where evidence in assessments does indicate that species are threatened by international trade-driven harvest, that this threat applies ubiquitously across the species' geographic range or over time. Also, it does not imply that all of these species require an immediate international policy response under CITES. This study adopted an evidentiary but precautionary approach (i.e., assumed greater rather than lesser risk to species) to reasonably deduce from available information in assessments whether there was evidence that international trade was a threat to species, regardless of the level of threat, and categorised species accordingly using the criteria developed for this purpose.

Informing decision-making in CITES

The 904 species categorised as Likely threatened by international trade but not included in CITES should be of interest to the CITES Parties because they are likely at some risk, depending on the scope and severity of threat from harvest for international trade. For example, species for which a small sub-population is threatened by harvest for international trade will likely require a different international policy response to a widespread species for which the majority of the global population is threatened by harvest for international trade. This also applies to broader conservation interventions beyond CITES trade controls. Species categorised on the Red List as Critically Endangered and Endangered should be

priorities for further evaluation of the impact of international trade because they face an extremely high or very high risk of extinction. However, key differences between the IUCN Red List Categories and Criteria and the CITES listing criteria (Supplementary Methods 2.6) mean that the combination of species being globally threatened on the Red List, including by BRU and/or determined to be Likely threatened by international trade, does not mean that they will, by default, meet the CITES listing criteria nor that proposals to amend the Appendices involving these species will necessarily be adopted by the Parties. The mechanism presented in this paper is a first step in identifying species that are Likely to be threatened by international trade and which may (or may not) benefit from CITES trade measures[17]. This determination will require further scrutiny of particular species, including whether they meet the CITES listing criteria, or not. This will require consideration of species' biology and ecology, associated harvest rates and trade levels, and the impact of offtake on populations spatially and temporally. Ideally, it should explicitly consider whether listing species in the Appendices will benefit the conservation of the species[17], before listing proposals are submitted. In some cases the Parties may decide that a species would benefit from greater trade regulation, but in others, they may decide that it may not, or that there are associated risks for species[47] and thus come to the decision not to submit, or subsequently adopt, a particular proposal.

Similarly, where species have been categorised as Likely threatened by international trade in our analyses and are included in the CITES Appendices, whether the species require further attention within the Convention (e.g., through inclusion in the RST or bespoke review mechanisms for Appendix I species) will depend on the scope and severity of threat from harvest for international trade. The above example contrasting a species for which only a small sub-population faces this threat compared with a widespread species for which the

majority of the global population faces the threat and likely difference in potential conservation response is relevant here. This also applies to conservation interventions beyond CITES.

It should be noted that this analysis does not mean that species categorised as Insufficient information or Unlikely definitely do not qualify for inclusion in CITES. There may be other evidence not yet included in assessments that can be brought to bear to show that these species do meet the CITES listing criteria and would benefit from inclsion in CITES. The mechanism presented is a way of helping to focus attention on those species that are most likely to be at risk from overexploitation for international trade, but it is not meant to be a method for excluding species from further, in-depth consideration for potential inclusion in CITES.

Our results may also be used to identify species that have improved in conservation status and can potentially be traded internationally on a sustainable basis. Where this is the case, the results (and future iterations) could provide the CITES Parties with key information to inform the potential relaxation of trade controls for species. Most obvious would be the transfer of species that are determined to be Unlikely to be threatened by international trade from Appendix I to II. This would need further scrutiny of the species, harvest and trade levels, and whether or not these measures would be likely to contribute to the conservation of the taxa[17].

Our results further indicate that of species with coded BRU threats in our dataset, the proportion also Likely threatened by international trade within each class is relatively small but increases when considering intentional use only (Extended Data Figure 3). There are

exceptions, including arachnids, anthozoans, cycads, sea cucumbers and fire corals, of which a high proportion of taxa that have coded BRU threats are Likely threatened by international trade. These species may warrant further examination from the CITES Parties and other conservation stakeholders regarding (i) which species are, and are not, included in CITES, and (ii) the impact of international trade to ensure that it is not detrimental to wild populations.

Red List assessments through time

Our study focussed on the Red List assessments at the time of our analysis and did not consider the length of time since the assessment was published or last updated. If the information in a Red List assessment was outdated at the time of our analysis, then theoretically our method could result in a species being coded as "Insufficient information" or "Unlikely" when in reality it is threatened by international trade or coded as 'Likely' when it is no longer threatened by international trade. These issues could be addressed by annotating any outputs provided to the CITES Parties (Fig. 4), highlighting species for which assessments need updating. It is worth reiterating that decisions taken by the Parties would be expected to rely not just on our results but on additional sources of information as well.

It is worth mentioning that several studies have attempted to use data on changes in Red List assessments over time to, among others, try to understand how listing in the CITES Appendices affects IUCN status over time[e.g., 60] . The main problem with most such analyses is that they fail to correctly account for genuine changes in status over time. This error is often incurred by making use of the tables listing genuine vs non-genuine changes on the Red List website. Unfortunately, doing so is an inappropriate use of Red List data. Rather, in order to undertake such analyses correctly, it is necessary to make use of the retrospectively

corrected Red List Categories that are generated through the process of producing the Red List Index (RLI). The Grey Wolf, *Canis lupus*, for example, was assessed as threatened in the mid-1980s on the Red List; it is LC today. In reality, the species has never, globally, been anything other than LC, so the corrected category in the 1980s, were one available, would be LC. Unfortunately, these corrected categories: i) are not currently available on the Red List website, and ii) are only available to certain previous points in time and for certain taxonomic groups (e.g., birds to 1988; mammals to 1996; amphibians to 1980).

Guiding future research

Our results also provide future research directions. In our analysis, 6,583 species were categorised as Insufficient information because it was not possible to determine whether they were threatened by international trade or not. These species would benefit from further research, both from the research community and Red List assessors, to determine whether international trade poses a threat, or not, and the severity of any threat. The Critically Endangered (n=1,059) and Endangered (n=2,149) species (see Supplementary Data 1) are obvious priorities for research effort given higher levels of extinction risk.

No funga species were evaluated to be Likely threatened by international trade though the number of funga species on the Red List is small. Further research on funga harvest and trade, and Red List assessments for these species, are needed[61].

5.2 Feasibility of recommendations

Recent research[47] has called for wildlife trade studies to evaluate the feasibility of associated policy recommendations. Here we suggest that periodic and systematic assessment of whether species on the Red List are Likely to be threatened by international trade, or not, could provide a starting point for CITES Parties to consider proposals to amend the Appendices for species negatively impacted by international trade. Our results could be used by Parties and other stakeholders during discussions on potential proposals ahead of CITES CoPs. We also propose that results from future analyses could be used to inform the CITES scientific committees about species that merit further examination between CoPs (Fig. 4). The scientific committees already discuss such matters during intersessional periods[62] (i.e., between CoPs). Our rapid mechanism for repeating and automating this analysis means that it should be feasible to conduct future analyses on a timeline that would be most beneficial to the Parties (i.e., at an appropriate time between CoPs).

Moreover, this process aligns directly with the CITES Strategic Vision[37], Objective 1.4 of which aims to ensure that “The Appendices correctly reflect the conservation status and needs of species”, and the mechanism presented here could therefore be expected to be of interest to the CITES Parties because it could help them to achieve this vision.

Disseminating the results to the CITES scientific committees could take a couple of forms. First, IUCN and collaborators could undertake this research and publish it in an open access format meaning it is available for Parties and other stakeholders at the appropriate time before each CoP meeting (see Fig. 4). Second, as this research has been undertaken by, and with, IUCN and UNEP-WCMC (UN Environment Programme World Conservation Monitoring Centre), which provide ongoing technical input into CITES meetings[63], future

results could be contributed in a similar way. They could be submitted in an "Information document" to the Animals and Plants Committee meetings or future CoPs, with the aid of a supportive Party, or through a formal agenda item to such meetings (Fig. 4). This would require further discussion with the CITES Parties, scientific committees and Secretariat. There is, however, precedent for IUCN submitting similar analyses to CITES meetings. For example, at the 28th meeting of the CITES Animals Committee (AC28) in 2015, IUCN submitted information on the conservation status of Asian snakes reported to occur in international trade to inform the future conservation management of Serpentes spp.[40]. As such, we consider both of these options regarding dissemination to the CITES Parties to be realistic. Subject to the number of new or additional species categorised as Likely threatened by international trade in future iterations of these analyses, the CITES scientific commmittees and Parties may wish to prioritise particular species or groups, and any formal process of doing so would require further discussion between the CITES scientific committees, Parties and Secretariat.

Two further points should be noted. First, while our analyses provide a starting point for Parties in thinking about potential proposals, in cases where it is subsequently deemed that species are being negatively impacted by international trade and would benefit from new or revised trade controls, the submission and adoption of any proposals to amend the Appendices will require political will by the world's governments. This pertains to the submission of proposals, and thereafter, their adoption. Second, our results are not limited to informing policy decisions in CITES. They may equally be used by Parties and other conservation stakeholders to inform national or international measures, interventions and/or policies to conserve biodiversity.

5.3 Implications for the IUCN Red List

The results in this article rely on the quality of the information contained in Red List assessments. During analysis, key fields from >12,395 assessments were read, including the rationale, threats, and use and trade text fields, and these fields were interpreted with coded fields on use and trade during manual coding. We make the following recommendations for improving the quality of data on use and trade in the Red List, which should be taken together with those presented in another recent study[34].

Use of language **–** Across taxonomic groups, it was difficult for a subset of species to determine from the text fields whether use and/or trade took place, whether use and/or trade constituted a threat to the species or not, and at what scale use and/or trade took place if it did (e.g., domestic or international). For example, the rationale, threats and use and trade sections of the assessment for *Carinotetraodon travancoricus* (a pufferfish) refer to capture for the "aquarium trade" and "pet trade" but do not capture the scale of the trade. This was compounded by variation in language between assessments relating to different taxonomic groups. As examples, the assessment for *Presbytis melalophos* (a primate) states that "trapping of the species for the illegal pet trade is a threat" whereas the assessment for *Taeniurops meyeni* (a stingray) refers to use and trade in terms of "landings" and "fishing pressure". While some assessments may include this information (e.g., in the coded fields on scale of use [e.g., subsistence level]), the accessibility of assessments for the type of analyses conducted here would be improved by assessors paying particular attention to the use of language in the threats and use and trade text fields, focusing on (i) whether use and/or trade in the species takes place, (ii) whether use and/or trade is a threat, and (iii) at what scale use and/or trade takes place (e.g., local, national and or/international).

Impact of harvest – Direct threats on the Red List are defined as "the proximate human activities or processes that have impacted, are impacting, or may impact the status of the taxon being assessed"[38]. For some assessments the threat to species from overexploitation is described as "trade". For example, the threats text field for *Cynops ensicauda* (a newt) states that "the species is also in the pet trade". However, in such cases the threat is undoubtedly from overharvesting (i.e., harvesting at a rate that exceeds the ability of populations to recover), which although may be trade-driven in some cases, trade in the species per se does not constitute the threat to the species.

Relatedly, the threat from overharvest depends on the harvest rate temporally and spatially and the impact on populations (e.g., influence on density). For many species there is an absence of data on population sizes and harvest rates, which makes the inclusion of such information in assessments difficult for many species. However, the Red List would be improved by assessors including, where possible, details on population size, harvest quantities and harvest rates, and the impact on populations in quantitative terms, including but not limited to, impacts on density and associated population growth rates over space and time. This would allow for a more robust assessment of the threat from harvest for use and/or trade, which may not be homogenous across species ranges or over time.

Use and trade data **–** Information on the use and trade of species is recommended, but not mandatory, for assessments. Future analyses of the type presented here would be improved if assessors included information on the use and trade of species when conducting new or updated assessments. More specifically, we recommend that when assessors document threats to species using intentional BRU threat codes (i.e., 5.1.1, 5.2.1, 5.3.1, 5.3.2, 5.4.1 and 5.4.2) they pay particular attention to answering the question "Is international trade a significant

driver of threat?” and coding the assessment accordingly (i.e., as yes, no, or unknown). This is facilitated by an integrity check warning which prompts assessors to answer this question if it is left blank. Having this in place since mid 2021, will hopefully encourage assessors to start using this field as a matter of routine.

6. Supplementary References

46. Scheffers, B., Oliveira, B. F., Lamb, I. & Edwards, D. P. Global wildlife trade across the tree of life. *Science* **366**(6461), 71–76. https://doi.org/10.1126/science.aav5327 (2019).

47. Challender, D.W.S., et al. Mischaracterising wildlife trade and its impacts may mislead policy processes. *Conserv. Lett.* e12832. https://doi.org/10.1111/conl.12832 (2021).

48. Fukushima, C. S., Mammola, S. & Cardoso, P. Global wildlife trade permeates the Tree of Life. *Biol. Conserv.* **247**, 108503. https://doi.org/10.1016/j.biocon.2020.108503 (2020).

49. Frank, E. G. & Wilcove, D. S. Long delays in banning trade in threatened species. *Science* **363**(6248), 686–688. https://doi.org/10.1126/science.aav4013 (2019).

50. Tulloch, V., Grech, A., Jonsen, I., Pirotta, V. & Harcourt, R. Cost-effective mitigation strategies to reduce bycatch threats to cetaceans identified using return-on-investment analysis. *Conserv. Biol.* **34**(1), 168–179. https://doi.org/10.1111/cobi.13418 (2020).

51. Gorobets, A. Wild fauna conservation: IUCN-CITES match is required. *Ecol. Indic.* **112**, 106091. https://doi.org/10.1016/j.ecolind.2020.106091 (2020).

52. Hutchinson, A., Stephens-Griffin, N. & Wyatt, T. (2022). Speciesism and the Wildlife Trade: Who gets Listed, Downlisted and Uplisted in CITES? *Int. J. Crime, Justice Soc. Democr* **11**(2), 191–209. https://doi.org/10.5204/ijcjsd.1945.

53. IUCN. IUCN Red List Categories and Criteria. https://portals.iucn.org/library/sites/library/files/documents/RL-2001-001-2nd.pdf (2012).

54. IUCN. Documentation Standards and Consistency Checks for IUCN Red List Assessments and Species Accounts. https://nc.iucnredlist.org/redlist/resources/files/1530703338-RL_Standards_Consistency.pdf (2013).

55. IUCN. Threat Impact Scoring System. https://nc.iucnredlist.org/redlist/content/attachment_files/Dec_2012_Guidance_on_Threat_Impact_Scoring_Revised.pdf (2012).

56. Guidelines for Using the IUCN Red List Categories and Criteria Version 14. http://cmsdocs.s3.amazonaws.com/RedListGuidelines.pdf (2019).

57. Webb, G.J.W. *Wildlife Conservation: In the Belly of the Beast*. Charles Darwin University Press, Darwin (2013).

58. IUCN. Rules of Procedure for IUCN Red List Assessments 2017–2020 Version 3.0. http://cmsdocs.s3.amazonaws.com/keydocuments/Rules_of_Procedure_for_Red_List_2017-2020.pdf (2016).

59. CITES. Resolution Conf. 12.11 (Rev. CoP18), Standard Nomenclature. https://cites.org/sites/default/files/document/E-Res-12-11-R18.pdf (2019).

60. Mialon, H.M., Klumpp, T. & Williams, M.A. International trade and the survival of mammalian and reptilian species. *Sci. Adv.* 8(1). https://doi.org/10.1126/sciadv.abh3442 (2022).

61. The Global Fungal Red List Initiative. The Global Fungal Red List Initiative. http://iucn.ekoo.se/en/iucn/welcome (2021).

62. CITES. Assessment of Three Bird Species Included in Appendix III (*Crax rubra*, *Meleagris ocellata* and *Penelope purpurascens*) Based on the Criteria of Resolution Conf. 9.24 (Rev. CoP16). https://cites.org/sites/default/files/eng/com/ac/28/E-AC28-22-01.pdf (2015).

63. IUCN. A review of IUCN engagement in the Convention on International Trade in Endangered Species of Wild Fauna and Flora (CITES). IUCN (2011).
