# Supplementary material for: Identifying species likely threatened by international trade on the IUCN Red List can inform CITES trade measures
Source: Nat Ecol Evol. 2023 Jul 6;7(8):1211–20. doi: 10.1038/s41559-023-02115-8 (PMC10545538; doi:10.1038/s41559-023-02115-8)
Supplement: Supplementary file 2 — Reporting Summary [file 41559_2023_2115_MOESM2_ESM.pdf]

nature portfolio

Corresponding author(s): Challender, D.W.S.

Last updated by author(s): Mar 16, 2023

Reporting Summary

Nature Portfolio wishes to improve the reproducibility of the work that we publish. This form provides structure for consistency and transparency in reporting. For further information on Nature Portfolio policies, see our Editorial Policies and the Editorial Policy Checklist.

Statistics

For all statistical analyses, confirm that the following items are present in the figure legend, table legend, main text, or Methods section.

n/a	Confirmed	
☐	☒	The exact sample size (*n*) for each experimental group/condition, given as a discrete number and unit of measurement
☐	☒	A statement on whether measurements were taken from distinct samples or whether the same sample was measured repeatedly
☐	☒	The statistical test(s) used AND whether they are one- or two-sided<br>*Only common tests should be described solely by name; describe more complex techniques in the Methods section.*
☒	☐	A description of all covariates tested
☐	☒	A description of any assumptions or corrections, such as tests of normality and adjustment for multiple comparisons
☐	☒	A full description of the statistical parameters including central tendency (e.g. means) or other basic estimates (e.g. regression coefficient) AND variation (e.g. standard deviation) or associated estimates of uncertainty (e.g. confidence intervals)
☐	☒	For null hypothesis testing, the test statistic (e.g. *F*, *t*, *r*) with confidence intervals, effect sizes, degrees of freedom and *P* value noted<br>*Give P values as exact values whenever suitable.*
☒	☐	For Bayesian analysis, information on the choice of priors and Markov chain Monte Carlo settings
☒	☐	For hierarchical and complex designs, identification of the appropriate level for tests and full reporting of outcomes
☒	☐	Estimates of effect sizes (e.g. Cohen's *d*, Pearson's *r*), indicating how they were calculated

Our web collection on statistics for biologists contains articles on many of the points above.

Software and code

Policy information about availability of computer code

Data collection	R (Version 4.0.3) was used to collect data from the IUCN Red List of Threatened Species and categorize species using automated coding and advanced automating coding approaches described in the article. Code for advanced automated coding of species is available from GitHub (link in article).
Data analysis	Custom code in R (Version 4.0.3) was used to categorize species using automated coding and advanced automating coding as described in the article. This entailed searching for particular keywords and text strings in selected text fields, and assessing the relevance of use-related threat codes, in IUCN Red List assessments. Code for advanced automated coding of species is available from GitHub (link in article). SPSS v.28 was used to estimate Fleiss' Kappa to compare manual, automated and advanced automated coding approaches.

For manuscripts utilizing custom algorithms or software that are central to the research but not yet described in published literature, software must be made available to editors and reviewers. We strongly encourage code deposition in a community repository (e.g. GitHub). See the Nature Portfolio guidelines for submitting code & software for further information.

Data

Policy information about availability of data

All manuscripts must include a data availability statement. This statement should provide the following information, where applicable:

- Accession codes, unique identifiers, or web links for publicly available datasets
- A description of any restrictions on data availability
- For clinical datasets or third party data, please ensure that the statement adheres to our policy

Provide your data availability statement here.

Field-specific reporting

Please select the one below that is the best fit for your research. If you are not sure, read the appropriate sections before making your selection.

☐ Life sciences ☐ Behavioural & social sciences ☒ Ecological, evolutionary & environmental sciences

For a reference copy of the document with all sections, see nature.com/documents/nr-reporting-summary-flat.pdf

Ecological, evolutionary & environmental sciences study design

All studies must disclose on these points even when the disclosure is negative.

Study description	We queried the IUCN Red List of Threatened Species (version 2020-1) to identify species potentially threatened by international trade. Using selection criteria we then categorized 21,745 species as being 'Likely' or 'Unlikely' to be threatened by international trade, or as having 'Insufficient information' to determine the likelihood of this threat based on available information in IUCN Red List assessments. We subsequently determined which of the species we evaluated to be 'Likely' threatened by international trade are, and are not, included in the CITES Appendices, which included aligning taxonomies between the IUCN Red List and CITES. We discuss the results in the context of all threats to species from biological resource use (BRU) on the IUCN Red List, including the scale of threat: local, national, or international.
Research sample	We used data on the 38,245 threatened and Near Threatened species on the IUCN Red List of Threatened Species (version 2020-1) (Methods and Supplementary Methods 2.1-2.3).
Sampling strategy	We queried the IUCN Red List of Threatened Species (version 2020-1) to identify species potentially threatened by international trade using defined criteria (Methods and Supplementary Methods 2.1-2.3).
Data collection	A.J. undertook initial queries on the IUCN Red List of Threatened Species (version 2020-1) and shared the results, which were in .csv files, with D.W.S.C. Details of the queries conducted are in Methods and Supplementary Methods 2.1-2.3. D.W.S.C. cross-referenced query outputs and constructed a MS Excel database including assessment data for all species for subsequent automated and manual coding. K.M. aligned taxonomies between the IUCN Red List of Threatened Species and CITES and stored the results in a MS Excel file. These results were shared with D.W.S.C. for analysis. A.T.P. conducted the automated coding and advanced automated coding and stored the outputs in .csv files that were shared with D.W.S.C. for analysis.
Timing and spatial scale	Data from the IUCN Red List (version 2020-1) were initially downloaded in May 2020 and used for the initial coding of species, which was completed in February 2021. Data were further collected as needed (e.g., for the advanced automated coding) up until, and including, September 2021.
Data exclusions	No data were excluded from the analyses.
Reproducibility	Data and code are available.
Randomization	Not relevant. No randomization needed.
Blinding	Blinding was not relevant to this study. Results relating to the categorization of species were unobtainable until manual coding had been completed independently by D.W.S.C., J.E.R., P.J.C. and K.M. and subsequently centralized, and until K.M. had completed taxonomy alignment between the IUCN Red List and CITES.

Did the study involve field work? ☐ Yes ☒ No

Reporting for specific materials, systems and methods

We require information from authors about some types of materials, experimental systems and methods used in many studies. Here, indicate whether each material, system or method listed is relevant to your study. If you are not sure if a list item applies to your research, read the appropriate section before selecting a response.

Materials & experimental systems

n/a	Involved in the study	
☒	☐	Antibodies
☒	☐	Eukaryotic cell lines
☒	☐	Palaeontology and archaeology
☒	☐	Animals and other organisms
☒	☐	Human research participants
☒	☐	Clinical data
☒	☐	Dual use research of concern

Methods

n/a	Involved in the study	
☒	☐	ChIP-seq
☒	☐	Flow cytometry
☒	☐	MRI-based neuroimaging
